# Arene-, Chlorido-, and Imido-Uranium Bis- and Tris(boryloxide) Complexes

**DOI:** 10.1021/acs.inorgchem.3c04275

**Published:** 2024-04-01

**Authors:** Xuhang Dan, Jingzhen Du, Shuhan Zhang, John A. Seed, Mauro Perfetti, Floriana Tuna, Ashley J. Wooles, Stephen T. Liddle

**Affiliations:** † Department of Chemistry and Centre for Radiochemistry Research, The University of Manchester, Oxford Road, Manchester M13 9PL, United Kingdom; ∥ Department of Chemistry Ugo Schiff, University of Florence, Via della Lastruccia 3, 50019 Sesto Fiorentino, Italy; ⊥ Department of Chemistry and Photon Science Institute, The University of Manchester, Oxford Road, Manchester M13 9PL, United Kingdom

## Abstract

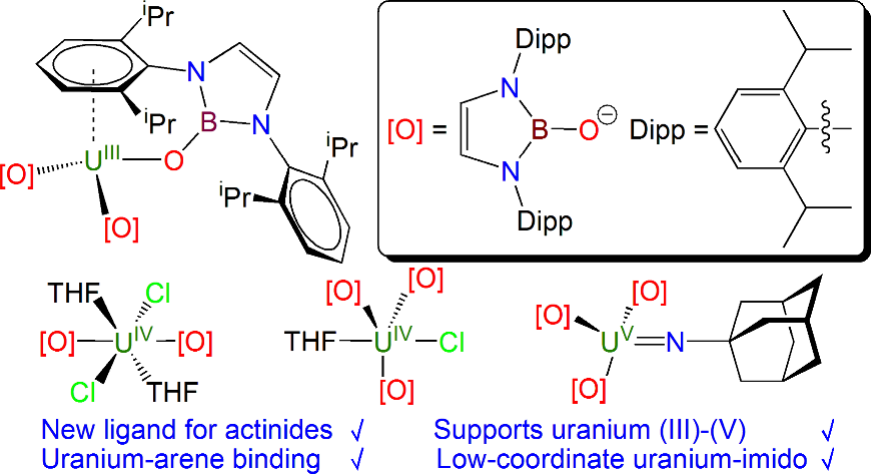

We introduce the boryloxide ligand {(HCNDipp)_2_BO}^−^ (NBO^Dipp^, Dipp = 2,6-di-isopropylphenyl) to actinide chemistry. Protonolysis of [U{N(SiMe_3_)_2_}_3_] with 3 equiv of NBO^Dipp^H produced the uranium(III) tris(boryloxide) complex [U(NBO^Dipp^)_3_] (**1**). In contrast, treatment of UCl_4_ with 3 equiv of NBO^Dipp^K in THF at room temperature or reflux conditions produced only [U(NBO^Dipp^)_2_(Cl)_2_(THF)_2_] (**2**) with 1 equiv of NBO^Dipp^K remaining unreacted. However, refluxing the mixture of **2** and unreacted NBO^Dipp^K in toluene instead of THF afforded the target complex [U(NBO^Dipp^)_3_(Cl)(THF)] (**3**). Two-electron oxidation of **1** with AdN_3_ (Ad = 1-adamantyl) afforded the uranium(V)–imido complex [U(NBO^Dipp^)_3_(NAd)] (**4**). The solid-state structure of **1** reveals a uranium–arene bonding motif, and structural, spectroscopic, and DFT calculations all suggest modest uranium–arene δ-back-bonding with approximately equal donation into the arene π_4_ and π_5_ δ-symmetry π* molecular orbitals. Complex **4** exhibits a short uranium(V)–imido distance, and computational modeling enabled its electronic structure to be compared to related uranium–imido and uranium–oxo complexes, revealing a substantial 5f-orbital crystal field splitting and extensive mixing of 5f |*m*_l_,*m*_*s*_⟩ states and *m*_*j*_ projections. Complexes **1**–**4** have been variously characterized by single-crystal X-ray diffraction, ^1^H NMR, IR, UV/vis/NIR, and EPR spectroscopies, SQUID magnetometry, elemental analysis, and CONDON, F-shell, DFT, NLMO, and QTAIM crystal field and quantum chemical calculations.

## Abstract 2

Through the synthesis of four uranium−boryloxide complexes, we introduce the boryloxide ligand {(HCNDipp)_2_BO}^−^ (NBO^Dipp^, Dipp = 2,6-di-isopropylphenyl) to actinide chemistry. The uranium(III)−arene derivative exhibits two modest, approximately equal uranium−arene δ-back-bonding interactions. The imido complex exhibits a short uranium(V)−imido distance, and computational modeling enabled its electronic structure to be compared to related uranium−imido and uranium−oxo complexes, revealing a substantial total 5f-orbital crystal field splitting and extensive mixing of 5f |*m*_*l*_,*m*_*s*_⟩ states and *m*_*j*_ projections.

## Introduction

It is often stated that ligand–metal complementarity is an essential component of the recipe for controlling the behavior of metal ions in coordination and organometallic complexes. This is certainly the case in synthetic actinide chemistry, where the nature of the ancillary ligands plays a decisive role in stabilizing or destabilizing actinide oxidation states, bonding motifs, reactivity, magnetism, and optical properties.^1−14^ In recent years, we have made extensive use of triamidoamine (Tren) ligands to stabilize a wide range of novel An–ligand multiple bonds,^15−28^ An–metal bonds,^15,29−35^ novel main group moieties,^36−43^ uranyl activation,^44^ small molecule activation,^45−48^ single-molecule magnets (SMMs),^49^ and novel photochemical rearrangements,^50^ and provide insight into fundamental f-block phenomena such as disproportionation, the inverse trans influence, pushing from below,^29,49,51−54^ and NMR covalency studies.^55,56^ However, there are situations where Tren ligands reach their limitations. For example, while high oxidation state uranium(V) and uranium(VI) oxos, imidos, and nitridos and even a neptunium(V)–oxo are now straightforwardly accessible with Tren ancillary ligands,^16,19,22,26−28,44−47,49,51,52,56^ to date, uranium(IV) has proven to be the highest accessible oxidation state when paired with softer elements such as phosphorus, arsenic, antimony, and bismuth, with all attempts to oxidize resulting in uranium(IV) products exclusively being isolated.^17,24,36,40^ Although alkoxides and aryloxides are logical alternatives on hard–soft acid–base (HSAB) grounds, they do not always possess the ideal steric profiles for specific applications.

We have previously utilized the boryloxide ligand {(HCNDipp)_2_BO}^−^ (NBO^Dipp^, Dipp = 2,6-di-isopropylphenyl)^57,58^ to prepare rare earth complexes^59^ including the dysprosium complex [Dy{OB(NDippCH)_2_}_2_(THF)_4_][BPh_4_] that exhibits a notably high SMM relaxation energy barrier.^59^ Having considered the structure of that dysprosium complex, we surmised that assembling three of those NBO^Dipp^ ligands at actinide ions might produce sterically encumbered complexes with one or at most two well-protected reaction pockets at the metal, resulting from a “picket-fence” arrangement of Dipp groups, while also being capable of stabilizing high oxidation state actinide ions on HSAB principles. We also considered that the boryl center could provide an electronic “buffer”, where the oxide can modulate its donor strength to a metal and transfer any excess electron density into the vacant boryl p orbital. Alternatively, if the boryl center is still electron deficient, it could make up the difference with π donation from the two α-nitrogen atoms in the NBO scaffold or not accept any N atom π donation if already electronically saturated. Further motivation stemmed from the fact that there are relatively few f-element boryloxide complexes,^60,61^ which as R_2_BO^–^ species are distinct from the far more prevalent borate derivatives,^62−78^ and they have very different steric profiles from NBO^Dipp^. This is because R_2_BO^–^ ligands tend to have the ligand sterics pointing away from a coordinated metal, whereas with NBO^Dipp^, they point toward the metal. Thus, we considered the NBO^Dipp^ ligand to have significant potential for metal–ligand complementarity.

Here, we report the introduction of the NBO^Dipp^ ligand into actinide chemistry through the synthesis of uranium(III), uranium(IV), and uranium(V) derivatives which showcases the flexibility of the NBO^Dipp^ ligand to stabilize a range of actinide oxidation states. The uranium(III) derivative exhibits a uranium–arene interaction, where structural, spectroscopic, and computational methods consistently suggest uranium–arene δ-back-bonding with approximately equal donation into the π_4_ and π_5_ δ-symmetry π* molecular orbital combinations of the arene. However, the uranium–arene interaction is evidently weak, as demonstrated by straightforward two-electron oxidation to provide an imido complex. NIR spectroscopic and computational studies on the imido enable its contextualization with existing related uranium–imido, uranium–nitrido, and uranium–oxo derivatives. These new complexes thus represent a new family of synthetic precursors for further elaboration.

## Results and Discussion

### Synthesis

**Scheme 1:**
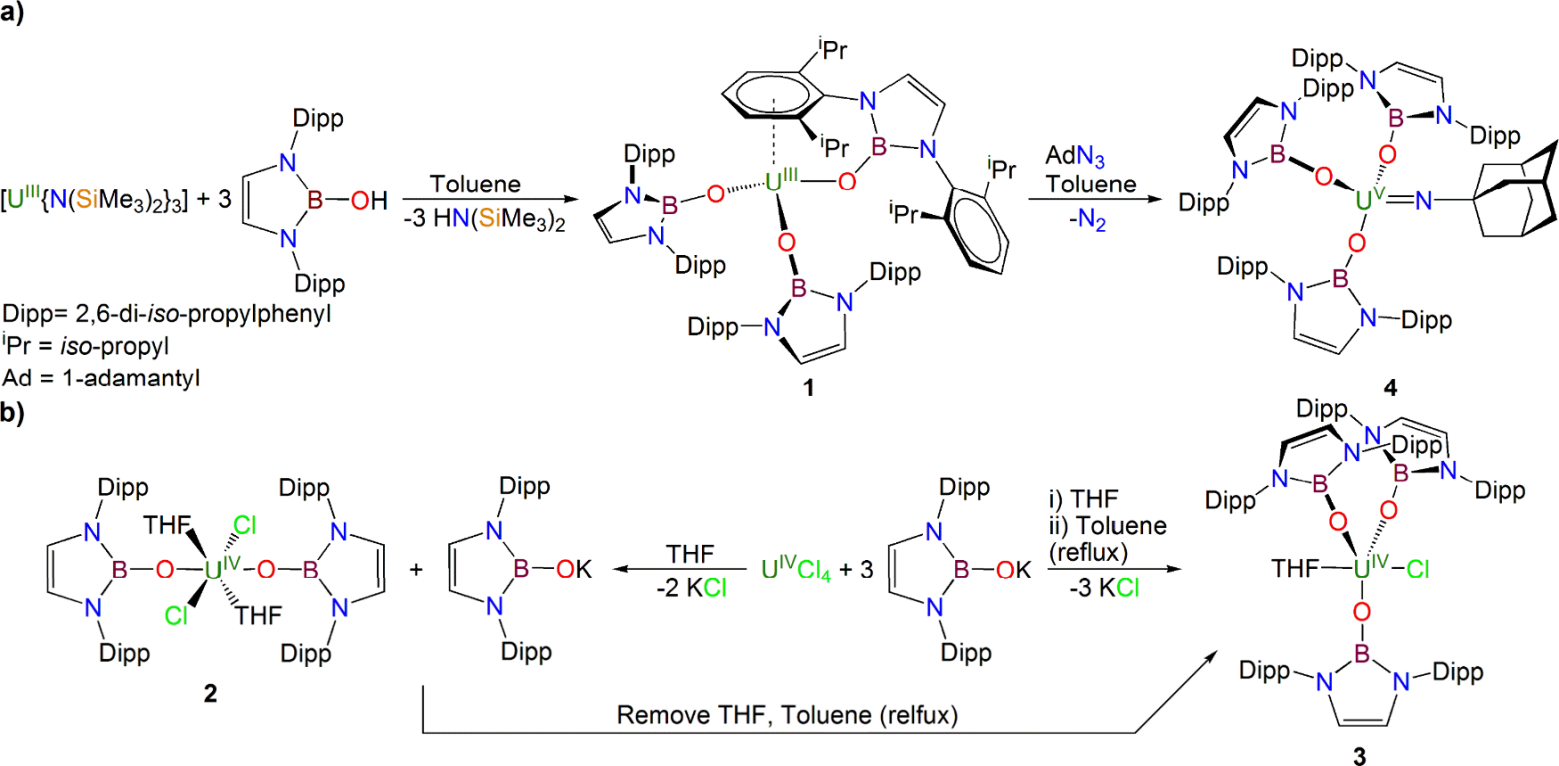
Synthesis of Complexes **1**–**4** (a) Reaction of [U{N(SiMe_3_)_2_}_3_] with 3 equiv of NBO^Dipp^H affords **1** with elimination of 3 equiv of amine, and subsequent treatment of **1** with AdN_3_ produces **4** with elimination of N_2_. (b) Reaction of UCl_4_ with 3 equiv of NBO^Dipp^K in THF produces **2**, 2 equiv of KCl, and 1 equiv of unreacted NBO^Dipp^K, and reflux in toluene after THF affords **3** and 3 equiv of KCl.

Treatment of [U{N(SiMe_3_)_2_}_3_]^79,80^ with 3 equiv of NBO^Dipp^H^57,58^ results, after a 2 day stir and workup, in the uranium(III)–tris(boryloxide) complex [U(NBO^Dipp^)_3_] (**1**) as an analytically pure dark purple solid in 51% yield, Scheme 1a; the HN(SiMe_3_)_2_ byproduct is conveniently removed, either when the reaction solvent is removed in vacuo or when the resulting solid is washed with pentane. Although the reaction is evidently driven thermodynamically by the formation of U–O bonds and the respective p*K*_a_ values of the protic components of the reaction, it is clearly kinetically sluggish. This likely reflects the kinetic barrier to installing three sterically demanding NBO^Dipp^ ligands at a single metal center, even one as large as uranium(III) (Shannon uranium(III) six-coordinate ionic radius = 1.025 Å).^81^ Support for this suggestion can be found in the issues encountered in the synthesis of the chlorido analogs (see below).

Compound **1** constitutes a potentially useful starting material, where the redox chemistry of uranium(III) may be utilized (see below for an example), and seeking to extend the range of uranium–NBO^Dipp^ synthetic precursors, we targeted a uranium(IV)–chlorido derivative for applications in salt elimination chemistry, Scheme 1b. Accordingly, we treated UCl_4_ with 3 equiv of NBO^Dipp^K in THF, and the green solution turned brown during a 16 h stir. After workup, we isolated yellow [U(NBO^Dipp^)_2_(Cl)_2_(THF)_2_] (**2**) in 80% crystalline yield. The third equivalent of unreacted NBO^Dipp^K was found to be in the mother liquor by ^1^H NMR spectroscopy. The fact that only 2 equiv of NBO^Dipp^ substituted onto uranium by salt elimination is consistent with the slow substitution by protonolysis that produces **1**. Repeating the reaction but refluxing in THF made no difference to the reaction outcome. However, when first stirring the 3:1 NBO^Dipp^K:UCl_4_ mixture in THF, then replacing the THF with toluene, and refluxing, a dark green solid was isolated after workup. Recrystallization from toluene afforded [U(NBO^Dipp^)_3_(Cl)(THF)] (**3**) as yellow-green crystals in 71% yield. We suggest that in THF the coordination sphere of uranium is permanently saturated with THF molecules, blocking installation of the third NBO^Dipp^ ligand but that in toluene any loss of THF is not compensated by the bulk solvent, thus opening up the coordination sphere at uranium to enable salt elimination and installation of the third NBO^Dipp^ ligand at uranium to occur. Alternatively, or in addition to, the partial solubility of KCl in THF may establish an equilibrium that is driven to products by the insolubility of KCl in toluene.

Experiments to derivatize **2** and **3** are ongoing and will be reported in due course. However, in a preliminary demonstration of the utility of **1** as a useful synthetic precursor the brown uranium(V)–imido complex [U(NBO^Dipp^)_3_(NAd)] (**4**, Ad = 1-adamantyl) was prepared in a straightforward and essentially quantitative two-electron oxidation of **1** with AdN_3_ with concomitant elimination of N_2_ (65% crystalline yield), Scheme 1a. Thus, although the coordinated arene in **1** may be regarded as providing steric protection of the uranium ion, it is labile enough to not impede further reactivity.

### Solid-State Structures

**Figure 1:**
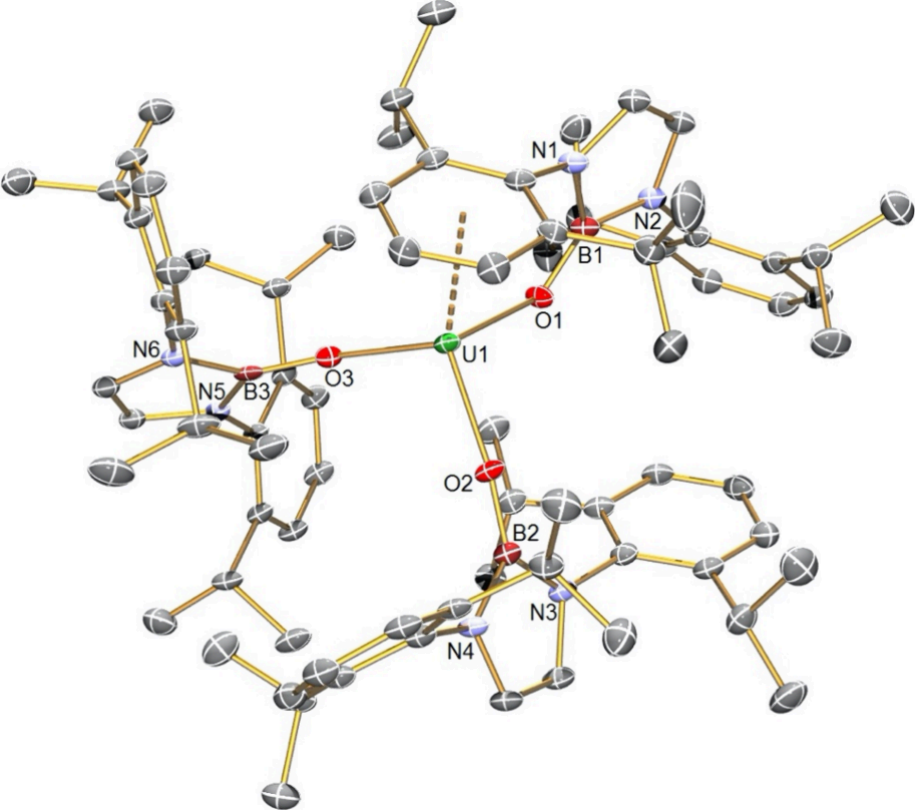
Molecular structure of **1** at 100 K with selected atom labels. Displacement ellipsoids are set at 40%, and hydrogen atoms and disordered components are omitted for clarity.

**Figure 2:**
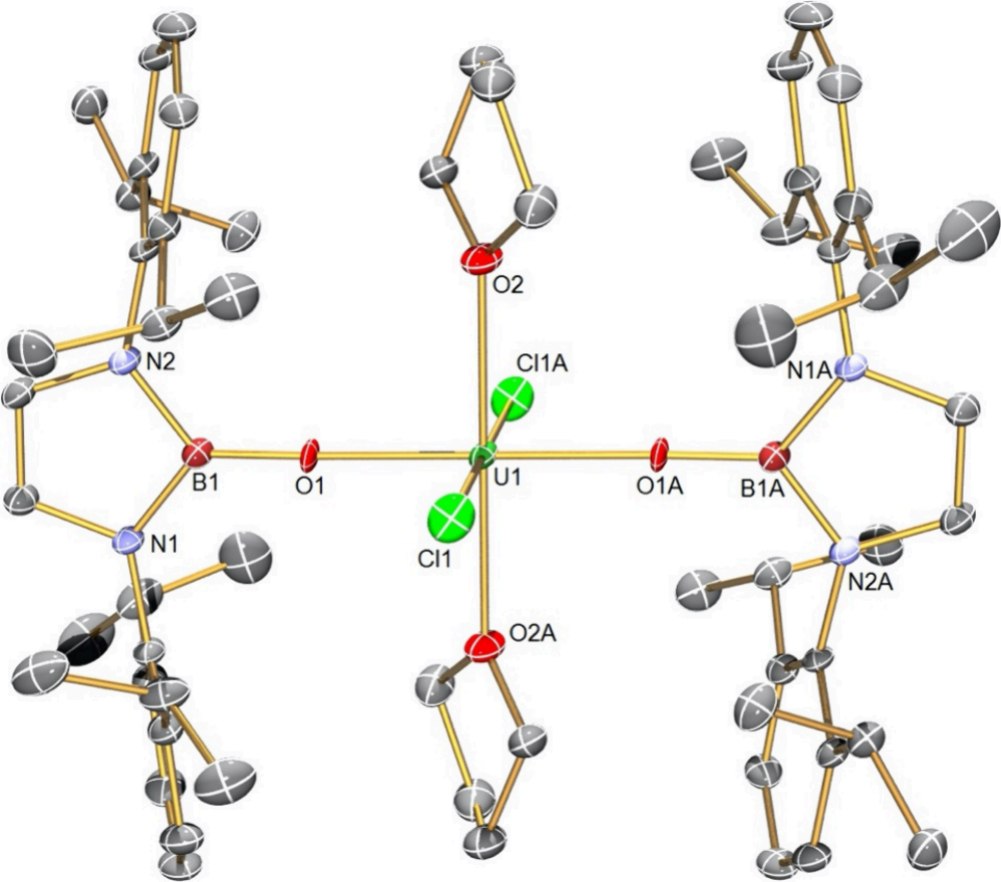
Molecular structure of **2** at 120 K with selected atom labels. Displacement ellipsoids are set at 40%, and hydrogen atoms and disordered components are omitted for clarity.

**Figure 3:**
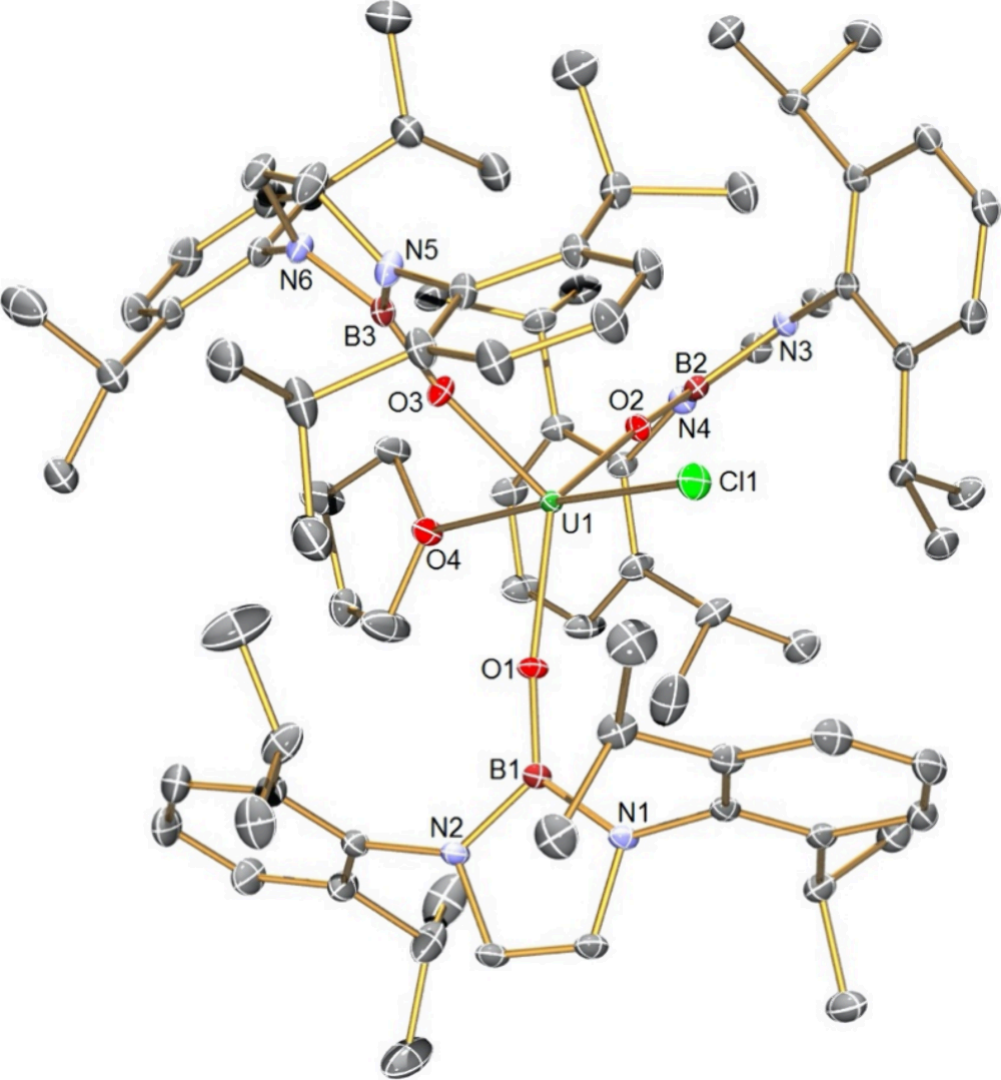
Molecular structure of **3** at 150 K with selected atom labels. Displacement ellipsoids are set at 40%, and hydrogen atoms and disordered components are omitted for clarity.

**Figure 4:**
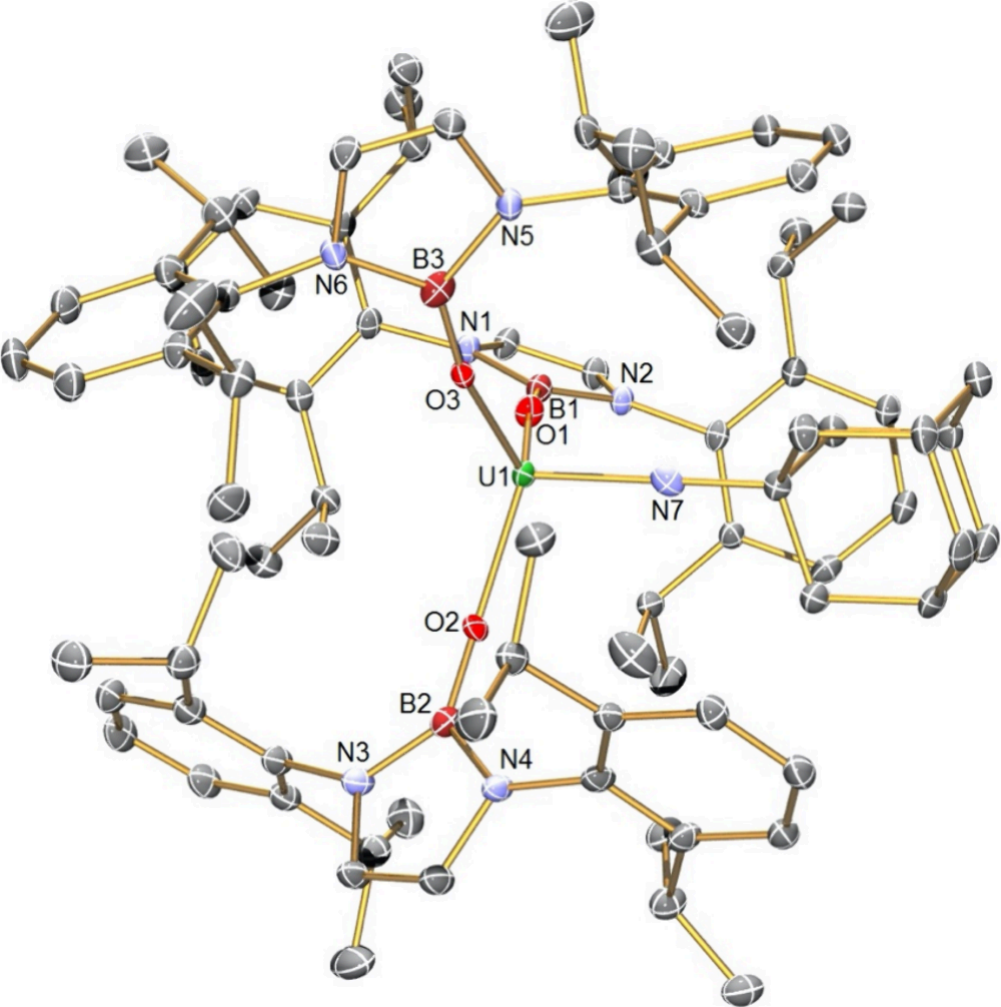
Molecular structure of **4** at 120 K with selected atom labels. Displacement ellipsoids are set at 30%, and hydrogen atoms and disordered components are omitted for clarity.

To confirm the formulations of **1**–**4**, their solid-state structures were determined by single-crystal X-ray diffraction, Figures 1–4 and Table S1.

Complex **1**, Figure 1, crystallizes with two of NBO^Dipp^ ligands coordinated in terminal monodentate modes with the third NBO^Dipp^ ligand coordinated as an η^6^-κ^1^-chelate, coordinated through one of the Dipp rings as well as the boryloxide O atom. Thus, counting the centroid of the arene ring as a coordination point, **1** adopts a distorted tetrahedral coordination geometry at uranium with a piano-stool geometry defined by the η^6^-arene and the three boryloxide O-donor centers. The three U–O distances are 2.217(3), 2.183(3), and 2.173(3) Å, similar to other uranium–boryloxide distances,^60,61^ with the longer U–O distance being associated with the chelating NBO^Dipp^ ligand, reflecting the bent U–O–B angle of 127.6(3)° in the former compared to the latter two U–O–B angles of 162.1(3)° and 162.4(3)° which are much closer to being linear. Although care must be taken when invoking π donation as a function of bond angles,^48,82,83^ the more linear U–O–B coordination linkages do, in principle, offer the possibility of stronger donation to uranium and hence shorter U–O bonds compared to the bent U–O–B linkage. The U–C distances span the range 2.913(4)–2.999(4) Å (average 2.970 Å) with a U–C_centroid_ distance of 2.616 Å, which falls in the middle to upper range of uranium(III)–arene distances,^84^ likely due to the strain of the chelate ring, which is evident in the slightly bent C_centroid_–C_ipso_–N_NBO_ angle of 168.2°. The coordinated arene ring is essentially planar (maximum rms deviations from the arene plane from −0.016 to 0.026 Å), but its C–C distances span the range 1.382(6)–1.421(6) Å (average 1.406 Å), which suggests some transfer of electron density into its π* orbitals because the average C–C distance in the other Dipp rings in **1** is ∼1.396 Å, though the difference is marginal.

Complex **2**, Figure 2, exhibits a pseudo-octahedral uranium(IV) ion situated on a crystallographic center of inversion, coordinated to two NBO^Dipp^, two chloride, and two THF molecules in an all-trans arrangement. The U–O_NBO_ distances are 2.127(2) Å, which is ∼0.05 Å shorter than the terminal U–O distances in **1**; this is around one-half the difference in the ionic radii of six-coordinate uranium(III) and uranium(IV) (1.025 vs 0.89 Å, respectively),^81^ but this likely reflects the very different formal coordination numbers and geometries of **1** vs **2**. The U–Cl and U–O_THF_ distances are unremarkable.

The structure of **3**, Figure 3, reveals a pseudo-trigonal bipyramidal geometry at uranium with the three NBO^Dipp^ ligands positioned in the equatorial plane and the chloride and THF ligands occupying the axial sites. The U–O_NBO_ distances span the range 2.124(2)–2.134(2) Å (average 2.130 Å) and by the 3σ criterion are statistically indistinguishable from the corresponding U–O_NBO_ distances in **2**. The U–Cl and U–O_THF_ distances in **3** (2.568(7) and 2.442(2) Å) are unremarkable, though we note that they are shorter and longer, respectively, than the corresponding distances in **2** (U–Cl = 2.648(2) Å; U–O_THF_ = 2.424(2) Å); acknowledging the different uranium coordination numbers of **2** and **3**, this most likely reflects the trans-influence effects of Cl vs THF in **3** and Cl vs Cl and THF vs THF ligand arrangements in **2**.

Complex **4**, Figure 4, contains a uranium(V) ion that is four coordinate, adopting a distorted tetrahedral geometry. The U–O distances span the range 2.129(5)–2.153(5) Å (average 2.140 Å) and by the 3σ criterion are indistinguishable from the corresponding U–O_NBO_ distances in **2** and **3**. This is likely due to the size decrease on going from uranium(IV) to uranium(V) being offset by the sterically demanding NBO^Dipp^ not being able to approach any closer due to interligand clashing, and also the presence of the strongly donating imido ligand. The U–N distance in **4** is short at 1.911(7) Å, which can be compared to U–N_imido_ distances of 1.945(2), 1.910(6), and 1.967(12) Å in [U(NAd){N(SiMe_3_)_2_}_3_],^85^ [U(NSiMe_3_){N(SiMe_3_)_2_}_3_],^85,86^ and [U(NAd)(Tren^TIPS^)] (Tren^TIPS^ = {N(CH_2_CH_2_NSi^i^Pr_3_)_3_}^3–^),^27^ respectively. The N–U–O angles in **4** span the range 99.7(2)–104.1(2)° (average 102.07°), between the ideal angles for trigonal monopyramidal (90°) and tetrahedral (109.5°), closer to the latter as found in [U(NAd){N(SiMe_3_)_2_}_3_]^85^ and [U(NSiMe_3_){N(SiMe_3_)_2_}_3_],^86^ but in contrast to [U(O){N(SiMe_3_)_2_}_3_]^87^ which is closer to the former.

### NMR and IR Spectroscopies

^1^H NMR and IR spectroscopic data for **1**–**4** can be found in the Supporting Information (Figures S1–S8). Despite the different coordination modes of the NBO^Dipp^ ligands in the solid-state structure of **1**, the ^1^H NMR spectrum of **1** spans the range from −2 to 10 ppm and indicates a symmetrical species in solution on the NMR time scale with 3-fold symmetry; this was not probed by variable-temperature experiments due to precipitation of **1**. However, two ^i^Pr-Me resonances and hence environments are observed, reflecting 12 Me groups that point toward the uranium ion and 12 that point away due to restricted rotation of the Dipp-^i^Pr groups. The ATR-IR spectrum of **1** is unremarkable, as expected.

The ^1^H NMR spectra of **2** and **3** span the ranges from −72 to 46 and from −20 to 13 ppm, which for the former is qualitatively an unusually large range for uranium(IV), but this likely reflects the varied coordination environment in that complex; the range for **3** is unexceptional for uranium(IV). The IR data for **2** and **3** are unremarkable but are consistent with their formulations.

The ^1^H NMR spectrum of **4** spans the range from −1 to 17 ppm, which is qualitatively as expected for a uranium(V) complex. The IR spectrum of **4** is as expected, and we note that there are absorptions around 1056–972 and 707–648 cm^–1^ consistent with the prediction of U=N stretches at 1000 and 663 cm^–1^ from an analytical frequencies calculation; those vibrations are best described as AdN=U bond stretches where the entire AdN unit moves together and at lower energy stretches where the U and Ad groups stretch out and in together about the N center.

### SQUID Magnetometry

**Figure 5:**
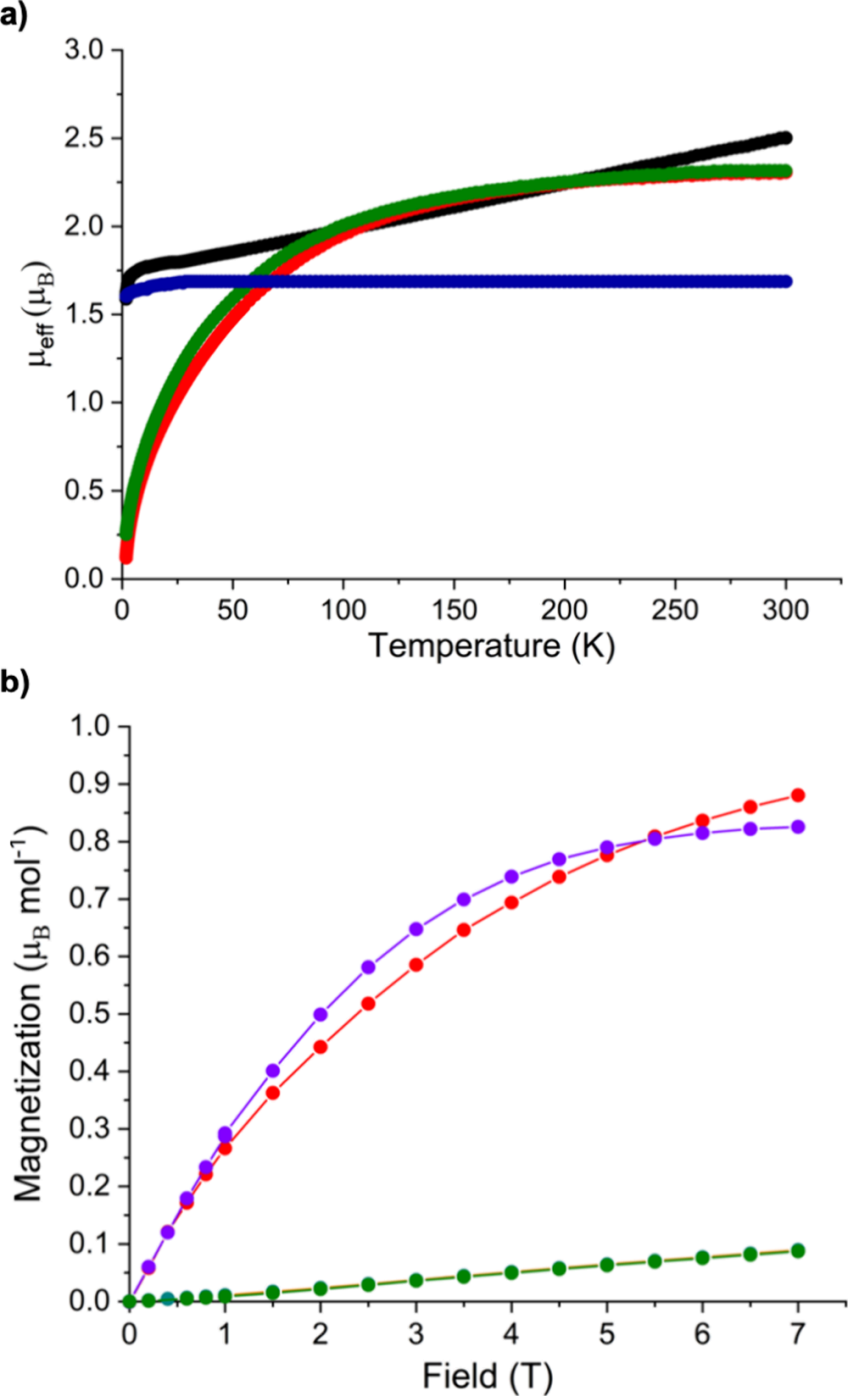
SQUID magnetometry data for **1**–**4**. (a) Variable-temperature μ_eff_ vs *T* data: **1** (black circles), **2** (red circles), **3** (green circles), and **4** (blue circles). (b) *M* vs *H* data at 2 K: **1** (purple trace), **2** (orange trace), **3** (green trace), and **4** (red trace). Note: the data for **2** and **3** are virtually identical. Lines are a guide to the eye only.

Variable-temperature SQUID magnetometry data for **1**–**4** can be found in the Supporting Information (Figures S9–S16) The uranium(III) formulation of **1** is confirmed by variable-temperature SQUID magnetometry of powdered **1**, Figure 5, where the effective magnetic moment is 2.50 μ_B_ at 300 K, and this decreases smoothly to ∼30 K, at which point there is a more rapid decrease, reaching 1.59 μ_B_ at 1.8 K; the high-temperature effective magnetic moment of **1** is low for uranium(III), but this has been observed for uranium(III)–aryloxides,^88,89^ though otherwise this behavior is typical of uranium(III).^9,88,90^ Magnetization vs field experiments at 2 K are approaching but do not reach saturation at the highest available field (∼0.9 μ_B_, 7 T), which is consistent with a Kramers uranium(III) ion.^91^

The variable-temperature SQUID magnetometry results of **2** and **3**, Figure 5, reveal effective magnetic moments of 2.31 and 2.31 μ_B_ at 300 K, respectively, and these values decrease smoothly over the temperature range reaching 0.12 and 0.25 μ_B_ at 1.8 K and tending to zero, which is typical of magnetic singlet uranium(IV) ions subject to modest temperature-independent paramagnetism.^9,88^ However, we do note that the 300 K μ_eff_ values for **2** and **3** are lower than the theoretical Russell–Saunders magnetic moment of 3.58 μ_B_ for ^3^H_4_ ions, but this is known for uranium–aryloxides.^88,92^ Nevertheless, at 2 K the magnetization vs field data for **2** and **3** are both still rising with no sign of approaching saturation up to the highest available field (∼0.09 μ_B_ for **2** and **3**, 7 T), which confirms the uranium(IV) formulations of **2** and **3**.^93^

The variable-temperature SQUID magnetometry data on **4**, Figure 5, reveal an almost constant effective magnetic moment of 1.69 μ_B_ across the temperature range measured, except for a small, decisive drop below 10 K to a final value of 1.60 μ_B_ at 1.8 K, likely resulting from depopulation of some low-lying states at very low temperature. This is similar to [U(NAd)(Tren^TIPS^)]^27^ and [U(O)(Tren^TIPS^)],^49^ but distinct from [U(NAd){N(SiMe_3_)_2_}_3_]^85^ and [U(NSiMe_3_){N(SiMe_3_)_2_}_3_]^85,86^ which exhibit effective magnetic moments of 2.47 and 2.12 μ_B_. The flat μ_eff_ vs *T* plot for **4** suggests that at high temperature either only the ground state is thermally populated or any excited states have similar effective magnetic moments. The magnetization vs field data for **4** at 2 K are approaching saturation, suggesting a well-isolated electronic ground state, though saturation is not quite achieved up to the highest available field strength available (∼0.8 μ_B_, 7 T). Based on our prior analysis of the electronic structure of uranium nitrido complexes as a guide^22^ and noting that precise values of magnetization data will vary depending on the ligand field and the *g* values, magnetization data of ∼0.9 μ_B_ imply a *m*_*j*_ ground state of 5/2 for **4** rather than 3/2, which would have a lower magnetization value of ∼0.5 μ_B_.

### EPR Spectroscopy

**Figure 6:**
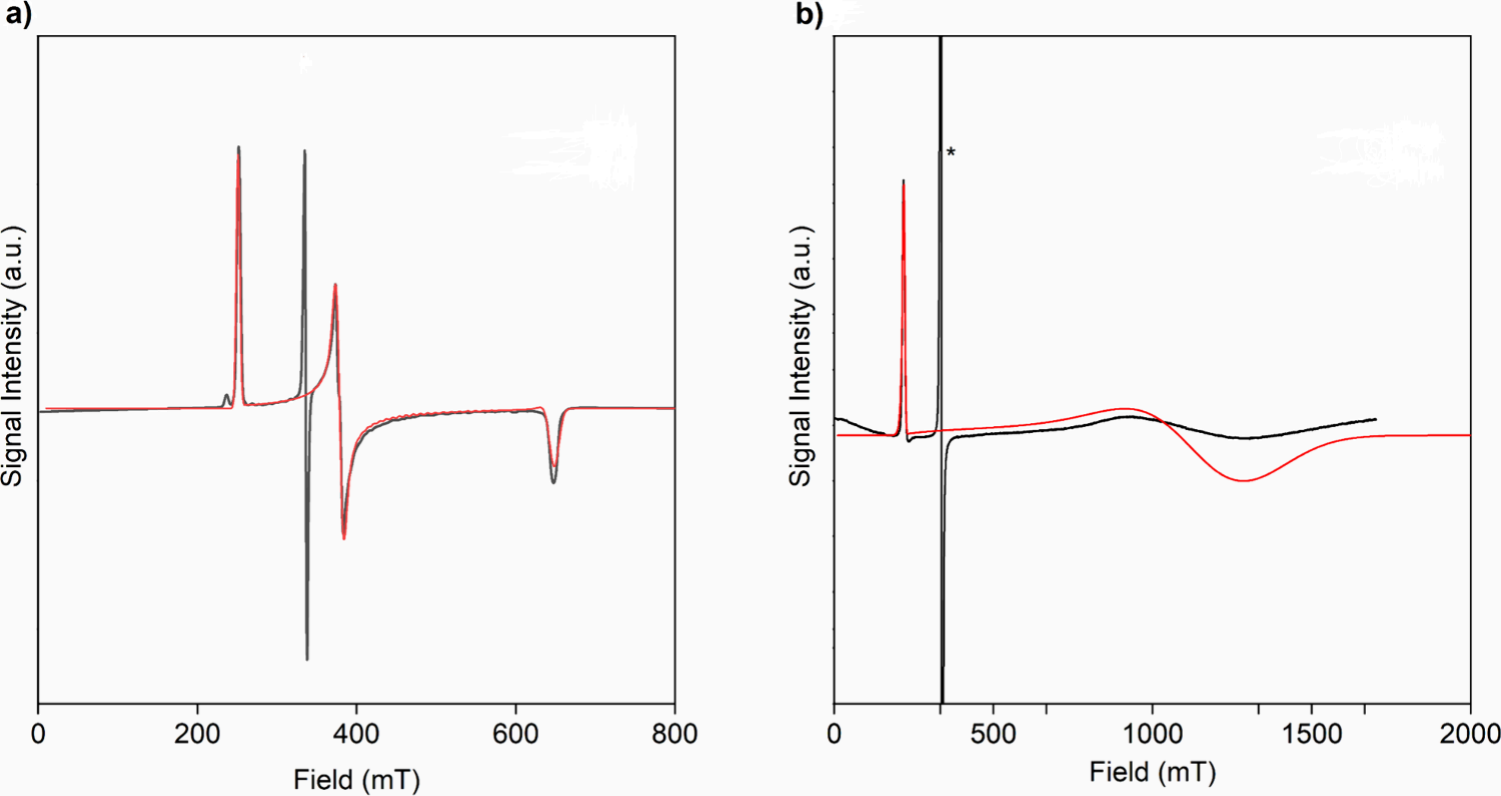
EPR X-band (∼9.4 GHz) spectra of powdered samples of **1** and **4** at 5 K. Black lines are experimental data, and red lines represent simulated data. (a) **1**: rhombic-type spectrum with *g* values of *g*_*x*_ = 2.680, *g*_*y*_ = 1.776, and *g*_*z*_ = 1.039 and a radical feature at *g* = 2.002 attributed to a minor organic radical impurity. (b) **4**: axial spectrum with *g*_*x*_ = *g*_*y*_ = 0.564 and *g*_*z*_ = 3.045. The sharp feature at *g* = 2.002 (marked with an asterisk (*)) is associated with a small portion of an organic radical impurity in the sample of **4**.

The X-band EPR spectrum of powdered **1**, Figure 6a, is consistent with the rhombic formulation anticipated from arene coordination to uranium(III) without a strong axial ligand,^89^ exhibiting anisotropic *g* values of *g*_*x*_ = 2.680, *g*_*y*_ = 1.776, and *g*_*z*_ = 1.039. In all samples examined there is also an isotropic feature at 2.002. We note that the *g* value for benzenide radical anions is also 2.002,^94^ and given the relative absorption intensities, the feature at *g* = 2.002 is assigned as a minor radical impurity. From μ_eff_ = 1/2[(*g*_*x*_^2^ + *g*_*y*_^2^ + *g*_*z*_^2^)^1/2^], the anisotropic EPR *g* values suggest that the magnetic moment of **1** at 5 K should be 1.68 μ_eff_, which is in good agreement with the measured effective magnetic moment at 5 K (1.73 μ_B_).

The X-band EPR spectrum of powdered **4**, Figure 6b, presents an axial-type spectrum with *g* values of *g*_*x*_ = *g*_*y*_ = 0.564 and *g*_*z*_ = 3.045. A *g* = 2.002 feature is also observed but is assigned as a minor organic radical impurity, likely of benzenide origin.^94^ Using μ_eff_ = 1/2[(*g*_*x*_^2^ + *g*_*y*_^2^ + *g*_*z*_^2^)^1/2^], the anisotropic EPR *g* values suggest that the magnetic moment of **4** at 5 K should be 1.57 μ_eff_, which is in good agreement with the measured effective magnetic moment at 5 K (1.63 μ_B_). In *C*_3*v*_ symmetry, a pure *m*_*j*_ = 3/2 state would be EPR silent (*g*_*x*_ = *g*_*y*_ = 0), as found for [U(O)(Tren^TIPS^)],^49^ because the *J* = ±1 EPR selection rule cannot be met; however, with changes to the geometry and ligand field and with *m*_*j*_ mixing, that selection rule can be met and *g* values of *g*_*x*_ = *g*_*y*_ = 0.1–0.5 and *g*_*z*_ = ∼2.4 would be anticipated. For *m*_*j*_ = 5/2, that is typically mixed with *m*_*j*_ = 7/2, *g* values of *g*_*x*_ = *g*_*y*_ = ∼0.3 and *g*_*z*_ = ∼3.7 are usually found. Thus, the observed *g*_*z*_ values for **4** can be compared to those of [U(NAd){N(SiMe_3_)_2_}_3_]^85^ (*g*_*z*_ = 3.60), [U(NSiMe_3_){N(SiMe_3_)_2_}_3_]^85^ (*g*_*z*_ = 2.50), and [U(O){N(SiMe_3_)_2_}_3_]^85,87^ (*g*_*z*_ = 2.17), suggesting that the ground state of **4** is *m*_*j*_ = 5/2, which is also consistent with the magnetometry data.

### UV/vis/NIR Spectroscopy

**Figure 7:**
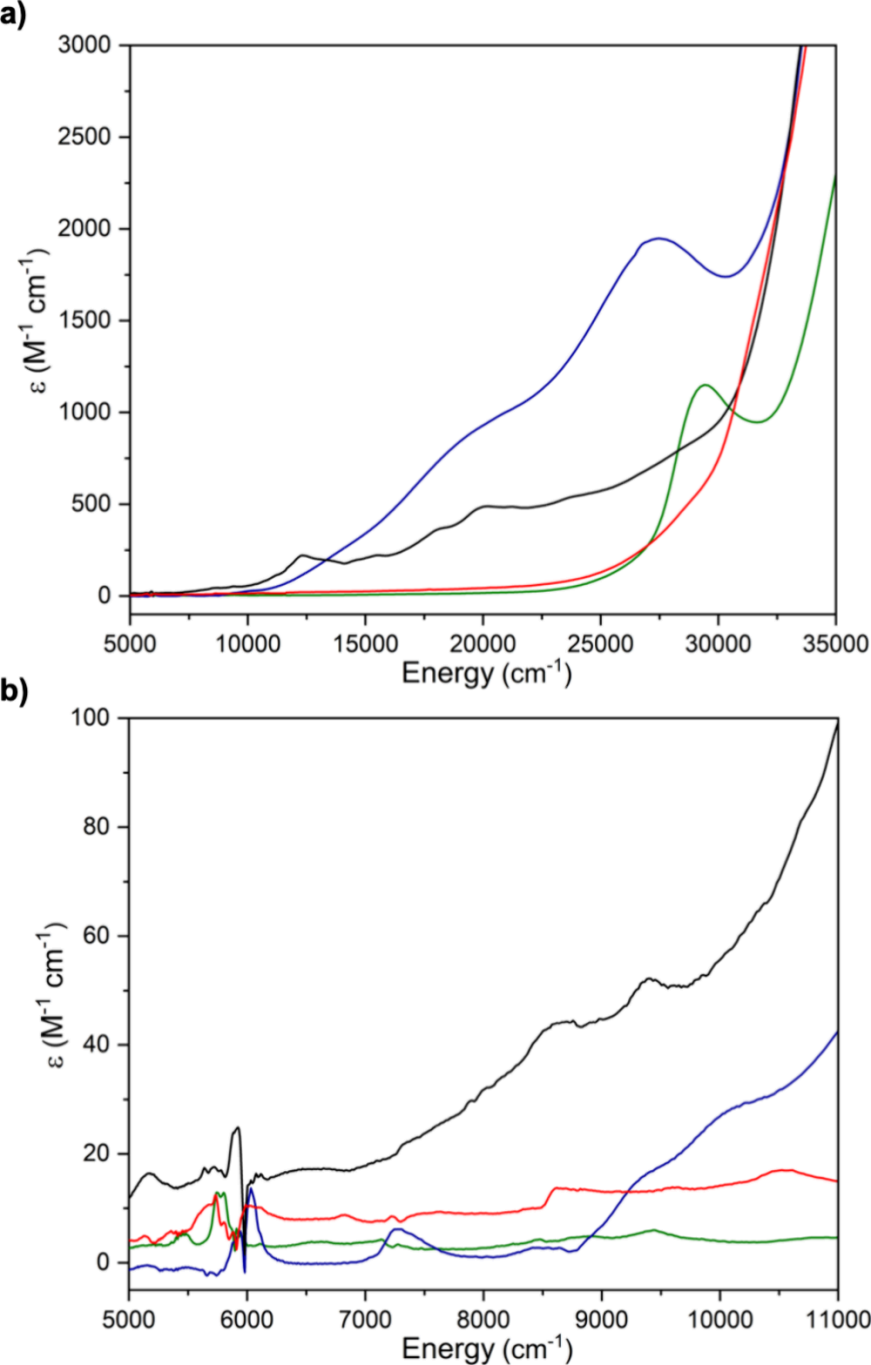
UV/vis/NIR spectra of **1** (black line; 1 mM in toluene), **2** (red line; 1 mM in THF), **3** (green line; 1 mM in THF), and **4** (blue line; 1 mM in toluene). (a) Spectra in the range from 5000 to 35000 cm^–1^. (b) Spectra in the range from 5000 to 11 000 cm^–1^.

UV/vis/NIR spectra for **1**–**4** were collected (see Supporting Information, Figures S17–S20). All complexes exhibit absorptions in the range from 25 000 to 30 000 cm^–1^, which correspond to π–π* transitions of the NBO^Dipp^ ligands. The UV/vis/NIR spectrum of **1**, Figure 7, exhibits absorptions in the range from 5500 to ∼26 000 cm^–1^, which are assigned as f–f transitions due to their approximate intensities (ε = ∼250–800 M^–1^ cm^–1^, these values should be regarded as upper bounds rather than intrinsic molar absorptivities).^9^ Above 26 000 cm^–1^, the spectrum is dominated by charge transfer bands. It is notable that there are not any absorptions in the region from 15 000 to 22 000 cm^–1^ with large enough intensities (∼1500 M^–1^ cm^–1^) to be assigned as f–d transitions. This suggests little stabilization of the 6d orbitals by the ligand field of **1** and that the f–d absorptions of **1** reside under the charge transfer bands at higher energy.

The UV/vis/NIR spectra of **2** and **3**, Figure 7, exhibit weak absorptions in the NIR region that are characteristic of uranium(IV) ions,^9^ and then, charge transfer bands tail into the visible region from the UV zone.

**Figure 8:**
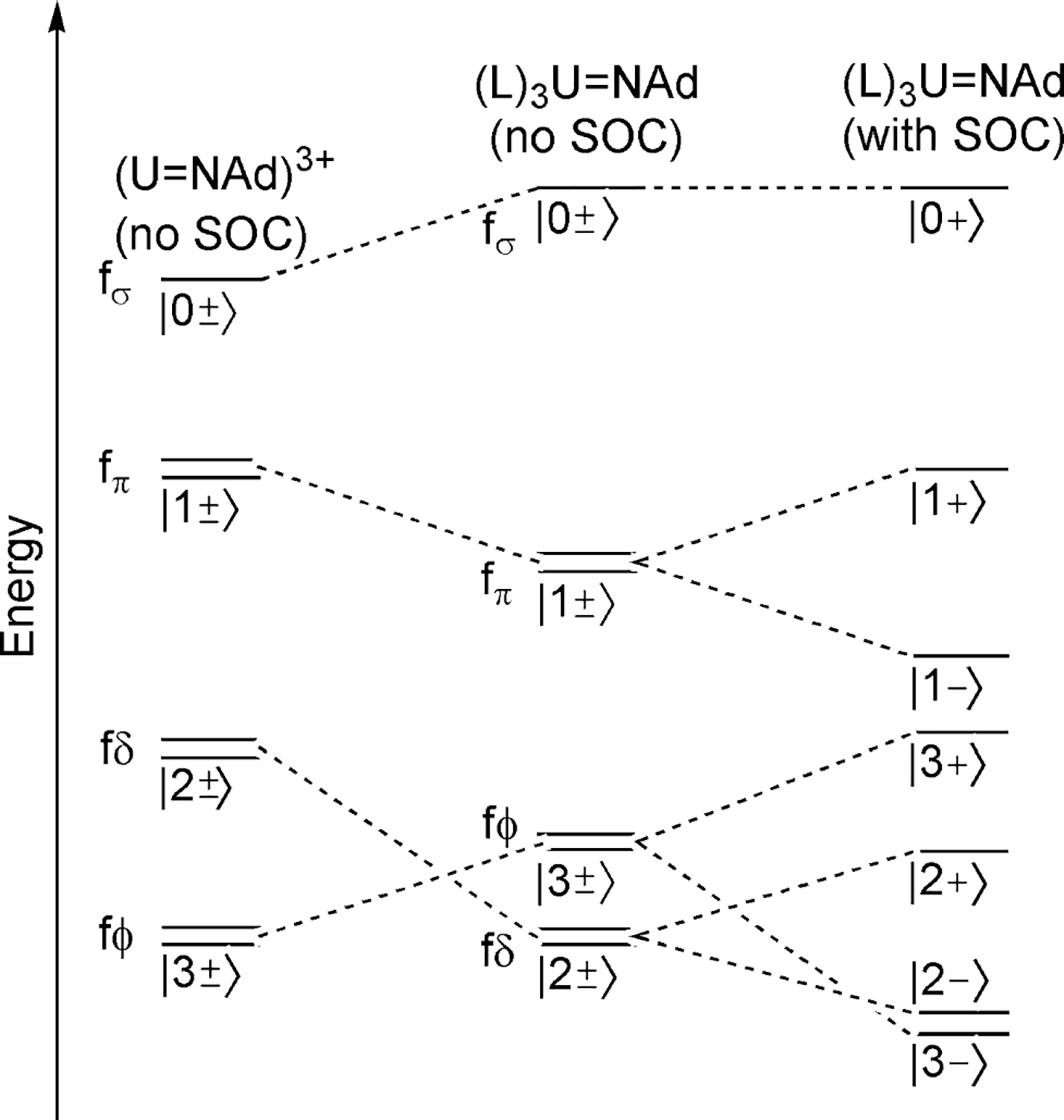
Qualitative zeroth-order splitting of the 5f^1^ states of **4**. Horizontal lines correspond to Kramers doublets, where only *m*_*l*_ ≥ 0 is shown and the states are shown as |*m*_*l*_,*m*_*s*_⟩ (*m*_*s*_ = +1/2 (+) and −1/2 (−) for brevity.

The UV/vis/NIR spectrum of **4**, Figure 7, exhibits absorptions in the NIR region that are characteristic of ^2^F_5/2_ to ^2^F_7/2_ intraconfigurational transitions of the uranium(V) ion in pseudo *C*_3*v*_ symmetry.^9^ To provide a framework for visualizing the following discussion on the characteristic uranium(V) absorptions observed in the NIR region of **4**, Figure 7, we use the approach of Eisenstein and Pryce,^95,96^ previously used by us to describe the electronic structure of uranium nitrido complexes,^22^ and Hayton and Lukens to describe the electronic structure of nitrido and imido complexes.^85,97^ In brief, using |*m*_*l*_,*m*_*s*_⟩ notation, the U–N_imido_ σ bond will derive from a N sp orbital combined with |0,±1/2⟩ (5f_σ_, two electrons) and the two π bonds from N p orbitals with |1, ±1/2⟩ (5f_π_, four electrons). The |2,±1/2⟩ (5f_δ_) would be nonbonding, and then, the |3,±1/2⟩ (5f_ϕ_) pair of orbitals will contain the 5f electron of uranium(V).^22^ There will then be the π* (*m*_*l*_ = ±1) and σ* (*m*_*l*_ = 0) orbital combinations.^22^ Using a zeroth-order 5f-splitting approach, where the energies of states vary but mixing of states is neglected, energetically these would be ordered *m*_*l*_ = ±3 < *m*_*l*_ = ±2 < *m*_*l*_ = ±1 < *m*_*l*_ = 0 for the (U=NAd)^3+^ fragment, Figure 8 (note only *m*_*l*_ ≥ 0 are shown for clarity). The crystal field (CF) and spin–orbit coupling (SOC) will modify the energies of the resulting individual Kramers doublet states, where it is assumed that the magnitudes of splitting are ordered imido-CF > SOC > NBO^Dipp^-CF.^22,85,93^ Now considering the NBO^Dipp^-CF, the presence of equatorial or near-equatorial ligands will modulate the energies of the *m*_*l*_ = ±3 and ±2 orbitals, producing an ordering of *m*_*l*_ = ±2 < *m*_*l*_ = ±3 < *m*_*l*_ = ±1 < *m*_*l*_ = 0, Figure 8. This is because the NBO^Dipp^ ligands will variously engage in σ- and π-antibonding interactions with the *m*_*l*_ = ±3 and ±2 orbitals, where the precise strength of those interactions will depend on how equatorial or pseudotetrahedral the position of those ligands is. The action of SOC will then further split the states as shown in Figure 8. The result is that the ground state will be |3,–1/2⟩ (*m*_*j*_ = 5/2) in pseudo-tetrahedral complexes or |2,–1/2⟩ (*m*_*j*_ = 3/2) in trigonal monopyramidal complexes. The baseline *g* values for |3,–1/2⟩ and |2,±1/2⟩ are 4 and 2, and then, they will vary depending on the exact CF and SOC. Hence, the EPR data for **4** are consistent with a |3,–1/2⟩ (*m*_*j*_ = 5/2) 5f_ϕ_ ground state, which is the same as that of [U(NAd){N(SiMe_3_)_2_}_3_]^85^ but in contrast to the |2,–1/2⟩ (*m*_*j*_ = 3/2) 5f_δ_ ground state of [U(O){N(SiMe_3_)_2_}_3_].^85,87^

The NIR region of **4** exhibits absorptions approximately at 5945, 6031, 7042, 7267, 8666, 9328, 10 121, and ∼17 500 cm^–1^, and these data are similar to those obtained for [U(NAd){N(SiMe_3_)_2_}_3_] (6245, 7202, 7568, 9144, 9973, and 15 434 cm^–1^)^85^ and [U(NSiMe_3_){N(SiMe_3_)_2_}_3_] (6097, 7258, 7467, 8734, 9414, and 16 955 cm^–1^).^85^ We note, as was also found for [U(NR){N(SiMe_3_)_2_}_3_] (R = Ad, SiMe_3_)^85^ and the CF computational analysis that follows, that there are too many NIR absorptions for **4** on the basis of the zeroth-order model. Since there would be predicted to be only four transitions above 5000 cm^–1^,^22,85^ a likely explanation is vibronic coupling. Certainly, the absorption at ∼17 500 cm^–1^ is very broad, likely from unresolved vibronic coupling—recall the U=NAd group stretching frequencies of ∼970 and ∼650 cm^–1^—and that this absorption is a shoulder on the side of a more intense feature. That this feature is so intense likely stems from intensity stealing (5f_σ_ and N sp orbital mixing)^98^ of this U–N σ* orbital. Similar features were also found with [U(NAd){N(SiMe_3_)_2_}_3_], [U(NSiMe_3_){N(SiMe_3_)_2_}_3_], and [U(O){N(SiMe_3_)_2_}_3_].^85^ The absorptions in the approximate range 7042–9328 cm^–1^ also appear to exhibit vibronic coupling, though this is not obviously resolved, but we note that the approximate separations of the three lowest lying absorptions are similar to the most energetic U–NAd stretching mode. Assuming that the peaks at ∼7042 and ∼7267 cm^–1^ are a split feature and likewise for the absorptions at ∼8666 and ∼9328 cm^–1^, then they are averaged (to 7155 and 8997 cm^–1^, respectively). Using the zeroth-order splitting, Figure 8, and discarding the lowest two absorptions from the analysis, the absorptions can be initially assigned as 7155 (5f_ϕ_, |3,+1/2⟩), 8997 (5f_π_, |1,–1/2⟩), 10 121 (5f_π_, |1,+1/2⟩), and 17 500 (5f_σ_, |0,+1/2⟩) cm^–1^, Table 1. The |2,+1/2⟩ (5f_δ_) and |2,–1/2⟩ (5f_δ_) states fall below the measurement window, and the |3,–1/2⟩ (5f_ϕ_) ground state is known from the EPR and magnetic data.

**Table 1:** Calculated Energies and Principal Components of the 5f States for Complex **4** Derived from CONDON 3.0 and F Shell^a^

	absorption energy (cm^–1^)					
zeroth-order assignment	observed	averaged	calculated	|*m*_*l*_,*m*_*s*_⟩ composition (%)	parent orbital	Russell–Saunders state and *m*_*j*_ projection
|0,+1/2⟩	17 500	17 500	17 293	35 |0,±1/2⟩	5f_σ_	55 ^2^F_7/2_ |±1/2⟩
				29 |±1,∓1/2⟩	5f_π_	22 ^2^F_7/2_ |±7/2⟩
				22 |±3,±1/2⟩	5f_ϕ_	9 ^2^F_5/2_ |±1/2⟩
				7 |∓3,±1/2⟩	5f_ϕ_	7 ^2^F_7/2_ |∓5/2⟩
				7 |∓2,∓1/2⟩	5f_δ_	7 ^2^F_5/2_ |∓5/2⟩
|1,+1/2⟩	10 121	10 121	10 489	61 |±1,±1/2⟩	5f_π_	86 ^2^F_7/2_ |±3/2⟩
				25 |±2,∓1/2⟩	5f_δ_	12 ^2^F_5/2_ |∓3/2⟩
				9 |∓2,±1/2⟩	5f_δ_	
				3 |∓1,∓1/2⟩	5f_π_	
|1,–1/2⟩	9328	8997	9392	34 |±1,∓1/2⟩	5f_π_	44 ^2^F_5/2_ |±1/2⟩
	8666			30 |0,±1/2⟩	5f_σ_	20 ^2^F_7/2_ |∓5/2⟩
				17 |∓2,∓1/2⟩	5f_δ_	20 ^2^F_7/2_ |±1/2⟩
				10 |±3,±1/2⟩	5f_ϕ_	10 ^2^F_7/2_ |±7/2⟩
				3 |∓3,±1/2⟩	5f_ϕ_	
|3,+1/2⟩	7267	7155	7525	33 |±2,±1/2⟩	5f_δ_	39 ^2^F_7/2_ |±5/2⟩
	7042			27 |∓3,∓1/2⟩	5f_ϕ_	27 ^2^F_7/2_ |∓7/2⟩
				17 |∓1,±1/2⟩	5f_π_	17 ^2^F_5/2_ |∓1/2⟩
				16 |0,∓1/2⟩	5f_σ_	16 ^2^F_7/2_ |∓1/2⟩
				6 |±3,∓1/2⟩	5f_ϕ_	
|2,+1/2⟩	b		3273	27 |±2,±1/2⟩	5f_δ_	36 ^2^F_5/2_ |∓1/2⟩
				24 |∓1,±1/2⟩	5f_π_	30 ^2^F_7/2_ |±5/2⟩
				19 |0,∓1/2⟩	5f_σ_	19 ^2^F_7/2_ |∓7/2⟩
				19 |∓3,∓1/2⟩	5f_ϕ_	8 ^2^F_5/2_ |±5/2⟩
				11 |±3,∓1/2⟩	5f_ϕ_	7 ^2^F_7/2_ |∓1/2⟩
|2,–1/2⟩	b		69	52 |±2,∓1/2⟩	5f_δ_	73 ^2^F_5/2_ |±3/2⟩
				21 |±1,±1/2⟩	5f_π_	14 ^2^F_7/2_ |∓3/2⟩
				14 |∓1,∓1/2⟩	5f_π_	13 ^2^F_5/2_ |∓3/2⟩
				13 |∓2,±1/2⟩	5f_δ_	
|3,–1/2⟩	b		0	64 |±3,∓1/2⟩	5f_ϕ_	75 ^2^F_5/2_ |±5/2⟩
				11 |±2,±1/2⟩	5f_δ_	17 ^2^F_5/2_ |∓1/2⟩
				10 |∓1,±1/2⟩	5f_π_	6 ^2^F_7/2_ |∓7/2⟩
				7 |0,∓1/2⟩	5f_σ_	
				6 |∓3,∓1/2⟩	5f_ϕ_	

a Magnetic data and energies were fitted simultaneously using CONDON 3.0 in *C*_3*v*_ symmetry, and F-shell was used to determine the composition of the states. F-shell outputs the composition as |*L*, *S*, *J*, *m*_*j*_⟩, and these were converted into the |*m*_*l*_, *m*_*s*_⟩ basis using Clebsch–Gordan coefficients.

b Outside the measurement window.

While the zeroth-order analysis is intuitive and provides a useful initial electronic structure description, it neglects symmetry-allowed mixing of the states. In order to probe this aspect, magnetic data and energies were fitted simultaneously using CONDON 3.0^99,100^ in *C*_3*v*_ symmetry (see Figure S21 for magnetic data fits), and F-shell^101^ was used to determine the full composition of the states (see the SI for details about the modeling procedure). F-shell outputs the compositions in the |*L*, *S*, *J*, *m*_*j*_⟩ basis (coupled basis); therefore, these were converted into the |*m*_*l*_, *m*_*s*_⟩ basis (uncoupled basis) using Clebsch–Gordan coefficients^102^ as reported in Table 1. The fit produced the crystal field parameters (expressed in Wybourne notation) *B*_2_^0^ = 5655, *B*_4_^0^ = 14 932, *B*_6_^0^ = 6791, *B*_4_^3^ = −16 885, *B*_6_^3^ = −6148, and *B*_6_^6^ = 10 887 cm^–1^ and a spin–orbit coupling value of ζ = 1761 cm^–1^_._ In general, the experimental magnetic data and NIR energies are modeled well, especially considering that a single ζ value is used in the calculations, but in reality, this and the associated orbital angular momentum would vary across different orbitals depending on their nature; for example, orbital mixing and hence reduced ζ and orbital angular momentum would be ordered 5f_σ_ > 5f_π_ ≫ 5f_ϕ_ ≈ 5f_δ_.

Since in **4** the SOC and CF parameters are comparable in strength, it would be predicted that there would be substantial mixing of the *J* = 7/2 and *J* = 5/2 manifolds. Inspection of Table 1 reveals that when mixing of states is modeled, the initial zeroth-order model is largely maintained. Indeed, the largest component of all states is the one predicted from the zeroth order except for the doublet at 7525 cm^–1^, which is composed by similar amounts of |2,+1/2⟩ and |3,–1/2⟩. It is noticeable that the calculated extent of mixing of states in **4** appears to be significantly more than that reported for [U(NR){N(SiMe_3_)_2_}_3_] (R = Ad, SiMe_3_),^85^ which we suggest stems from a larger crystal field for **4** resulting in more mixing overall. Hence, it would seem that the NBO^Dipp^ ligand brings more crystal field splitting than the bis(trimethylsilyl)amide, consistent with their O vs N donor natures. Therefore, Table 1 also lists each component as its Russell–Saunders state and *m*_*j*_ projection, showing that they are indeed significantly mixed, and largely the calculated *m*_*j*_ ordering (|±1/2⟩ > |±3/2⟩ > |∓1/2⟩ > |±5/2⟩ > |∓1/2⟩ > |±3/2⟩ > |±5/2⟩) for **4** is essentially the same as that calculated for [U(NAd){N(SiMe_3_)_2_}_3_] and [U(N)(Tren^TIPS^)]^−^ (|±1/2⟩ > |±3/2⟩ > |±5/2⟩ > |±7/2⟩ > |±1/2⟩ > |±3/2⟩ > |±5/2⟩);^22,85^ again, where there are differences (the third and fourth absorptions), the second largest component of each *m*_*j*_ projection is the same as that for [U(NAd){N(SiMe_3_)_2_}_3_] and [U(N)(Tren^TIPS^)]^−^. This emphasizes that although **4** has pseudo-*C*_3*v*_ symmetry (and is in reality lower symmetry), the strength of the imido ligand confers substantial effective axial symmetry, as found for [U(E){N(SiMe_3_)_2_}_3_]^−^ (E = NSiMe_3_, O).^93^

Although it should be acknowledged that there is some uncertainty in the precise CF splittings of **4** (∼17 500 cm^–1^), [U(NAd){N(SiMe_3_)_2_}_3_] (15 434 cm^–1^), [U(NSiMe_3_){N(SiMe_3_)_2_}_3_] (16 955 cm^–1^), and [U(O){N(SiMe_3_)_2_}_3_] (20 262 cm^–1^),^22,85^ due to the broadness and shoulder character of their U–E_σ*_ (E = NR, O) absorptions, it is instructive to consider them in qualitative terms. The larger CF splitting of **4** vs [U(NAd){N(SiMe_3_)_2_}_3_] can be primarily rationalized on the basis that the U=NAd distance in **4** is ∼0.04 Å shorter than that in the latter,^85^ and then, secondarily, the NBO^Dipp^ ligands also contribute to a larger CF. For **4** vs [U(NSiMe_3_){N(SiMe_3_)_2_}_3_], the U=NR distances are essentially the same,^86^ so even though the U=NSiMe_3_ σ* antibonding state should be higher in energy than U=NAd on a like for like basis (because the silylimido is less strongly donating than the alkylimido so will have slightly more stabilized bonding orbitals), the similar U=NR distances coupled to the NBO^Dipp^ ligands in **4** seemingly result in a slightly larger net CF for **4** compared to [U(NSiMe_3_){N(SiMe_3_)_2_}_3_]. Since the U=E (E = O, NR) distance is the principal driver for the energy of the UE_σ*_ state, **4** has a smaller CF splitting than [U(O){N(SiMe_3_)_2_}_3_] because the latter has a U=E distance that is ∼0.09 Å shorter^87^ than that in **4**, and this seems to outweigh any secondary NBO^Dipp^ vs amide CF effects.

### Computational Characterization

In order to further understand complexes **1**–**4**, we performed GGA LDA BP86 DFT calculations on the whole structures (Tables S2–S5). The gas-phase geometry-optimized structures (Table S6) match the experimental solid structures well overall, with U–O_NBO_, U–O_THF_, U–Cl, B–O, and B–N distances computed to within 0.03 Å and 5°. Computed bond orders, charges, spin densities, natural localized molecular orbital (NLMO), and quantum theory of atoms in molecules (QTAIM) data are compiled in Tables S6–S8. We note a discrepancy between experimental and computed U–C_arene_ distances in **1** (Δ = 0.13 Å), which likely arises from this being a relatively “soft” interaction and that the calculations are free of solid-state effects in the experimental structure of **1**. Geometry optimization using the hybrid PBE0 functional only marginally improved the discrepancy of the U–C_arene_ distance (Δ = 0.09 Å), so we examined the crystallographic coordinates with heavy atoms frozen and H atom positions geometry optimized and found only minor changes to the electronic structure of **1**. Thus, to provide an internally consistent set of data, we focus on the geometry-optimized BP86 data here. We conclude that the BP86 data provide qualitatively reliable models of the electronic structures of these two complexes since prior work has shown good bond order and NBO/NLMO analysis agreement between BP86 and experimentally (NMR) benchmarked hybrid B3LYP chemical shift anisotropy calculations.^55,56,103^

**Figure 9:**
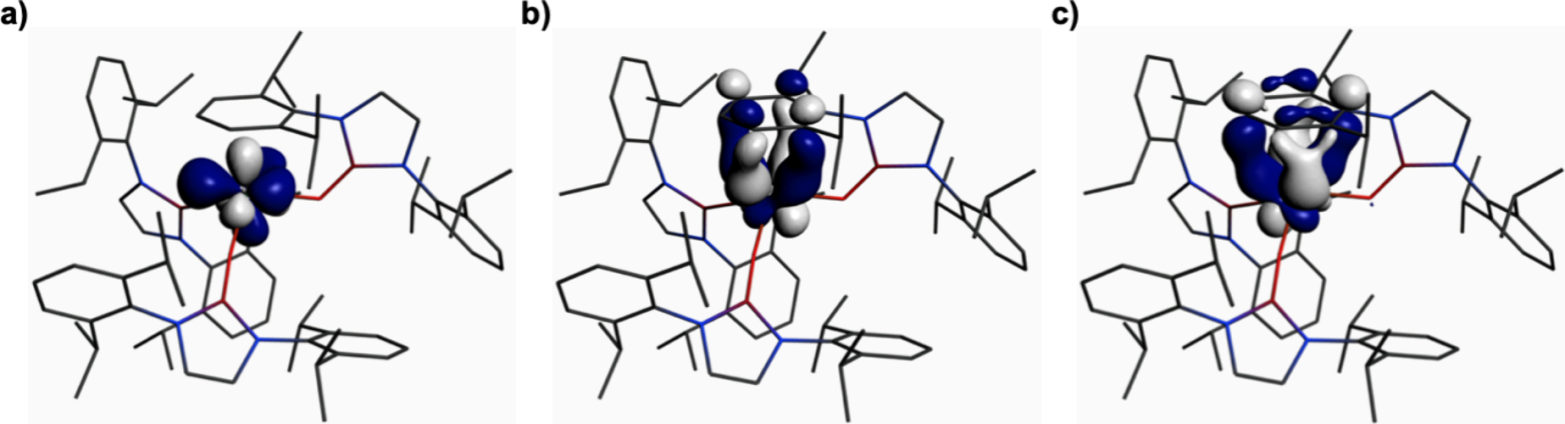
Selected frontier Kohn–Sham molecular orbitals of **1**: (a) HOMO (5f, 376a, −2.942 eV), (b) HOMO–1 (δ bond, 375a, −3.226 eV), and (c) HOMO–2 (δ bond, 374a, −3.257 eV). Hydrogen atoms are omitted for clarity.

The computed uranium MDC_q_ charges for **1**–**4**, Table S6, are 2.52, 2.24, 2.40, and 3.01 and are as anticipated for uranium ions in +3, +4, +4, and +5 oxidation states, respectively. The corresponding MDC_m_ spin densities of −2.51, −2.24, −2.25, and −1.31 are consistent for **2**, **3**, and **4** in terms of their formal f^*n*^ counts (*n* = 2, 2, and 1, respectively) augmented by net donation of electron density from the ligands. However, the spin density for **1** is less than the anticipated value of 3 for a 5f^3^ ion, suggesting that the uranium ion in **1** is a net exporter of electron density. The arene bonded to uranium has a computed net MDC_q_ charge of −0.94 (C_6_ ring, 3 × H, 2 × C, 1 × N atoms), and the C_6_ unit has a total net MDC_m_ spin density of 0.73. Confirmation of uranium–arene interactions is obtained by visualization of the α-spin frontier Kohn–Sham molecular orbitals (KSMOs) of **1**, Figure 9. Specifically, although the HOMO is a singly occupied electron of largely 5f character (5f/6d/7s, 83.93/1.40/9.67%), HOMOs−1 and −2 while both being principally of 5f character evidence modest carbon 2p-orbital contributions from δ-bonding interactions (U 5f/6d:C 2p HOMO−1, 70.08/5.04:18.06%; HOMO−2, 64.76/5.39:20.35%). The presence of two distinct but similarly composed and quasi-degenerate δ bonds is significant because it suggests almost equal population of both the π_4_ and the π_5_ δ-symmetry π* molecular orbitals of the arene; hence, no Jahn–Teller distortion of the arene,^104−106^ which would induce ring-puckering,^107,108^ occurs (recall the planar nature of the coordinated arene in the solid-state structure above). The presence of a U–arene interaction in **1** likely reflects its low-coordinate and electron-rich 5f^3^ formulation compared to **2**–**4**, which have higher coordination numbers and are 5f^2^ or 5f^1^. The KSMOs of **2** and **3** are unremarkable (Figures S22 and S23). Visualization of the α-spin KSMOs of **4** (Figure S24) reveals that the HOMO is an essentially pure 5f orbital (5f/6d, 93.66/5.48), HOMOs−4 and −5 are the U=N π bonds, and HOMO−21 is the U=N σ bond. However, to obtain a more intuitive bonding description, we focus on NLMO analysis (see later).

The U–O_NBO_ Nalewajski–Mrozek bond orders average 1.46, 1.52, 1.49, and 1.38 for **1**, **2**, **3**, and **4**, Table S6. This shows that the NBO^Dipp^ ligands are strong donors and reflects the increasing formal uranium charges on moving from trivalent **1** to tetravalent **2** and **3**. The fall in U–O_NBO_ bond order in **4** is likely due to the presence of the strong imido donor ligand. Reflecting that the U–arene δ-symmetry bonding interactions exhibit only modest carbon contributions, the U–C Nalewajski–Mrozek bond orders average 0.31, which is certainly consistent with those interactions being weak given the apparent loss of the uranium–arene interaction in solution as evidenced by ^1^H NMR spectroscopy. Although the changes are small, Table S6, the variation of the B–O and B–N Nalewajski–Mrozek bond orders generally confirms that when the U–O_NBO_ bond order increases/decreases, the B–O and B–N bond orders decrease/increase and increase/decrease, respectively. This reflects the aforementioned electronic buffering role of the boryl as the oxide donates less and more to electron-rich and -poor metals, respectively. The U–N_imido_ Nalewajski–Mrozek bond order is 2.80, reflecting that the imido ligand is a triple-bond donor in the KSMO scheme.

**Figure 10:**
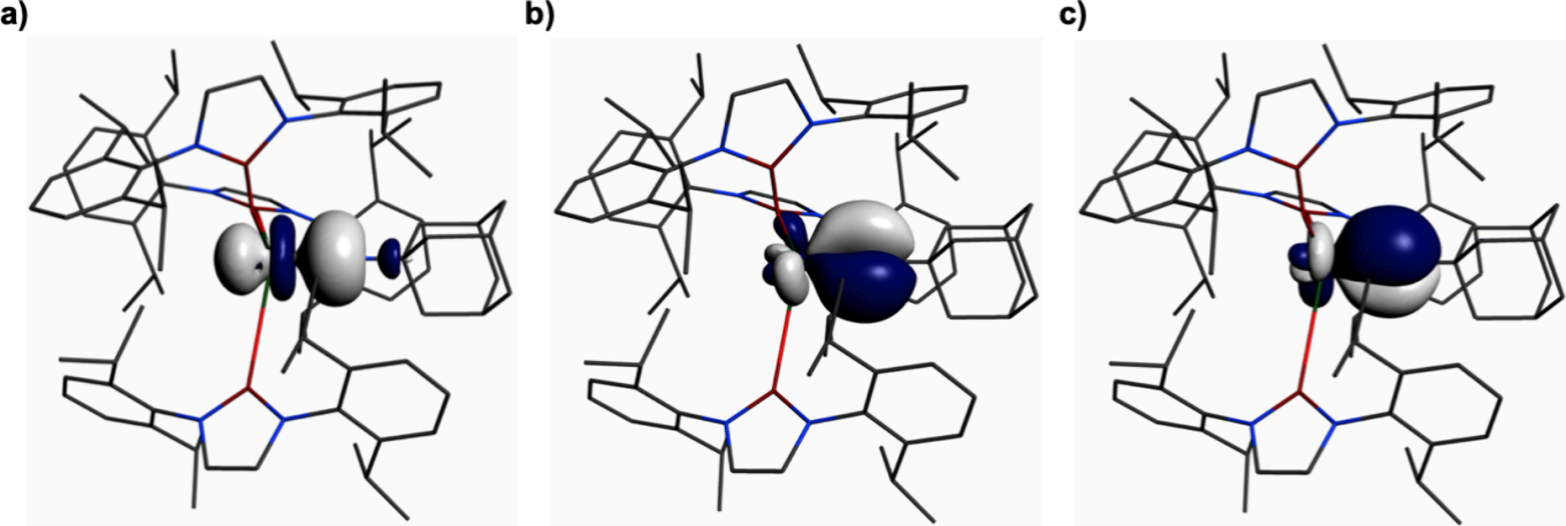
Selected natural localized molecular orbitals of **4**: (a) U=NAd σ bond, (b) U=NAd π bond, and (c) U=NAd π bond. Hydrogen atoms are omitted for clarity.

Complexes **1**–**4** provide an opportunity to examine the variation of the U–O_NBO_ bond as a function of the uranium oxidation state, and NLMO data are compiled in Table S7. The NLMO analysis reveals rather polar U–O_NBO_ bonds, and although the entire range of uranium contributions is only 3–8%, it can be generally noted that the uranium contributions increase with increasing oxidation state and the uranium 5f character tends to dominate over 6d contributions (5f:6d ≈ 2:1 for σ bonds and ∼3:1 for π bonds). Complex **2** exhibits the largest uranium contributions to the U–O_NBO_ bonds, likely reflecting the presence of two neutral THF donors. This can also be seen in the U–Cl bonds, where the uranium contributes more to the U–Cl bonds in **2** than **3**. NLMO analysis of **4**, Figure 10, reveals a U=N σ bond that is 11% uranium and 88% N character. The uranium component is composed of 1/3/38/59% 7s/7p/6d/5f character, and the nitrogen is 65:35 2s:2p character. The U=N π bonds are essentially the same and are composed of 22% and 77% uranium and nitrogen contributions, respectively. The uranium component is 0/0/13/87% 7s/7p/6d/5f character, and the nitrogen components are 100% 2p character.

To gain a topological- rather than orbital-based perspective, we examined the 3,–1 bond critical point QTAIM data for **1**–**4**, Table S8. As expected, the data reveal slightly polar–covalent B–O and B–N bonds (ρ_av_ = 0.21 and 0.19 e per Bohr^3^; *H*_av_ = −0.15 and −0.16 hartree per Bohr^3^, respectively), whereas the U–O_NBO_ bonds are borderline ionic (ρ_av_ = 0.10 e per Bohr^3^) and much weaker in terms of energy (*H*_av_ = −0.04 hartree per Bohr^3^). In contrast, the U–N_imido_ bond is a much stronger interaction, with ρ and *H* values of 0.19 e per Bohr^3^ and −0.14 hartree per Bohr^3^, respectively, and being close to zero, the ε value of 0.05 confirms a U–N triple bond.^109^ Overall, these values are similar to data previously calculated for [U(NSiMe_3_){N(SiMe_3_)_2_}_3_]^27^ but indicate a slightly stronger U=N bond than that calculated for [U(NR)(Tren^TIPS^)] (R = SiMe_3_, Ad)^27^ but, as expected, a weaker U=N bond than the oxo and nitrido bonds in [U(O){N(SiMe_3_)_2_}_3_],^87^ [U(O)(Tren^TIPS^)],^49^ [U(N)(Tren^TIPS^)]^*n*^ (*n* = 0, −1),^27,28^ and [Np(O)(Tren^TIPS^)].^16^ Lastly, the QTAIM data for the U–arene interactions in **1** reveal ρ_av_ and *H*_av_ values of 0.03 e per Bohr^3^ and −0.01 hartree per Bohr^3^, respectively, and, hence, in agreement with the analysis earlier, rather weak uranium–arene interactions. The uranium–arene ε_av_ value of 0.49 reflects the asymmetry of the individual orbital coefficient bonding interactions that construct the uranium–arene δ bonds.

## Conclusions

In conclusion, furthering the application of the boryloxide NBO^Dipp^ ligand in f-element chemistry, we have introduced this ligand to uranium chemistry. This has furnished a uranium(III)–tris(boryloxide) complex that features a uranium–arene interaction, two chlorido derivatives, and a uranium(V)–imido complex. Although the preparation of these complexes is thermodynamically favorable, it is notable that there is kinetic hindrance installing three sterically demanding NBO^Dipp^ ligands at a single metal center, even one as large as uranium(III). The coordinated arene may provide a useful protecting-group role but is evidently flexible enough to not impede reactivity as evidenced by preliminary work that has accessed a uranium(V)–imido complex by a two-electron oxidation strategy.

DFT studies qualitatively evidence modest uranium(III)–arene δ bonding, which appears to be approximately equal donation into the π_4_ and π_5_ δ-symmetry π* orbitals of the arene; this accounts for an apparent lack of Jahn–Teller distortion of the arene despite the fact it is shown experimentally and computationally to be carrying excess open-shell spin density. NIR and computational analysis of the imido complex shows that it has a similar CF to [U(NSiMe_3_){N(SiMe_3_)_2_}_3_], a larger CF than [U(NAd){N(SiMe_3_)_2_}_3_], but, as expected, a weaker CF than [U(O){N(SiMe_3_)_2_}_3_]. However, replacing amides with the NBO^Dipp^ ligand results in much more mixing of the |*m*_*l*_,*m*_*s*_⟩ states and Russell–Saunders *m*_*j*_ projections, but overall, it is the case that their orderings approximate well to a basic zeroth-order model that neglects mixing of states.

The synthesis of uranium(III), uranium(IV), and uranium(V) complexes supported by a common NBO^Dipp^ ligand demonstrates the versatility of this boryloxide ligand over several oxidation states of uranium and has provided an opportunity to examine the U–NBO^Dipp^ interactions over three uranium oxidation states. This versatility can in part be ascribed to the “buffer” nature of the boryl enabling the NBO^Dipp^ ligand to vary its electron donation as required. Hence, the NBO^Dipp^ ligand promises to stabilize complementary or new bonding motifs in actinide chemistry compared to existing Tren, amide, and aryloxide ligand classes and also suggests potential for further elaboration in lanthanide chemistry. Certainly, the NBO ligand class demonstrates the importance of ligand–metal cooperativity to bring new vistas to f-element chemistry, and work in that regard is underway in our laboratory.

## Supporting Information Available

Experimental and computational procedures; crystallographic details for **1**–**4**; NMR, IR, and UV/vis/NIR spectroscopic details for **1**–**4**; SQUID magnetometric characterization data for **1**–**4**; computational data for **1**–**4** (PDF)
