# Supplementary material for: Arene-, Chlorido-, and Imido-Uranium Bis- and Tris(boryloxide) Complexes
Source: Inorg Chem. 2024 Apr 1;63(21):9588–601. doi: 10.1021/acs.inorgchem.3c04275 (PMC11134490; doi:10.1021/acs.inorgchem.3c04275)
Supplement: Supplementary file 1 — ic3c04275_si_001.pdf [file ic3c04275_si_001.pdf]

Arene-, Chlorido-, and Imido-Uranium *Bis* and *Tris*(boryloxide) Complexes

Xuhang Dan†,[1] Jingzhen Du†,[1,2] Shuhan Zhang†,[1] John A. Seed,[1] Mauro Perfetti,[3] Floriana Tuna,[4] Ashley J. Wooles,[1] Stephen T. Liddle[1]*

[1] Department of Chemistry and Centre for Radiochemistry Research, The University of Manchester, Oxford Road, Manchester, M13 9PL, UK.

[2] Current Address: College of Chemistry, Zhengzhou University, Zhengzhou, 450001, China.

[3] Department of Chemistry Ugo Schiff, University of Florence, Via della Lastruccia 3, 50019, Sesto Fiorentino, Italy.

[4] Department of Chemistry and Photon Science Institute, The University of Manchester, Oxford Road, Manchester, M13 9PL, UK.

† These authors contributed equally to this work.

*Email: steve.liddle@manchester.ac.uk

Experimental Details

General

All manipulations were carried out using Schlenk techniques, or an MBraun UniLab glovebox, under an atmosphere of dry dinitrogen. Solvents were dried by passage through activated alumina towers and degassed before use. All solvents were stored over potassium mirrors except for ethers and

dichloromethane, which were stored over activated 4 Å sieves. Deuterated solvents were distilled from potassium, degassed by three freeze-pump-thaw cycles and stored under dinitrogen. Glassware was silylated with HMDS under reduced pressure. $[U\{N(SiMe_3)_2\}_3]$,[1,2] UCl_4,[3] $HOB(NDippCH)_2$ ($NBO^{Dipp}H$, Dipp = 2,6-di-*iso*-propylphenyl) and $NBO^{Dipp}K$,[4,5] were prepared according to published procedures. All other reagents were used directly from commercial sources without further purification.

Single crystals were examined variously on either a Rigaku XtalLAB Synergy-S diffractometer equipped with a Hypix 6000HE photon counting pixel array detector with mirror-monochromated Cu Kα radiation (λ= 1.5418 Å) or a Rigaku FR-X diffractometer, equipped with a HyPix 6000HE photon counting pixel array detector with mirror-monochromated Cu Kα (λ = 1.5418 Å) radiation. Intensities were integrated from a sphere of data recorded on narrow (0.5° or 1.0°) frames by ω rotation. Cell parameters were refined from the observed positions of all strong reflections in each data set. Gaussian grid face-indexed absorption corrections with a beam profile correction were applied. The structures were solved either by dual methods using SHELXT[6] and all non-hydrogen atoms were refined by full-matrix least-squares on all unique F^2 values with anisotropic displacement parameters with exceptions noted in the respective cif files. Hydrogen atoms were refined with constrained geometries and riding thermal parameters; $U_{iso(H)}$ was set at 1.2 (1.5 for methyl group) times U_{eq} of the parent atom. The largest features in final difference syntheses were close to heavy atoms and were of no chemical significance. CryAlisPro was used for control and integration,[7] and SHELXL and Olex2 were employed for structure refinement.[8,9] ORTEP-3 and POV-Ray were employed for molecular graphics.[10,11] ^1H spectra were recorded on a Bruker 400 spectrometer operating at 400.1 MHz; chemical shifts are quoted in ppm and are relative to tetramethylsilane (^1H). Samples were prepared in the glovebox and placed in J. Young PTFE 5mm screw-topped borosilicate NMR tubes. FTIR spectra were recorded on a Bruker Alpha spectrometer with a Platinum-ATR module in a glovebox. UV/Vis/NIR spectra were recorded on a Perkin Elmer Lambda 1050 spectrometer where

data were collected in 1cm path length cuvettes and were run versus the appropriate reference solvent. Variable-temperature magnetic moment data were recorded in an applied direct current (DC) field of 0.1 T on a Quantum Design MPMS3 superconducting quantum interference device magnetometer using doubly recrystallized powdered samples. Samples were carefully checked for purity and data reproducibility between several independently prepared batches for each compound examined. Samples were ground and immobilized in eicosane in a sealed 5 cm length of borosilicate glass NMR tube mounted in the center of a drinking straw, with the straw fixed to the end of an MPMS 3 sample rod. Care was taken to ensure complete thermalization of the sample before each data point was measured. Diamagnetic corrections were applied using tabulated Pascal constants and measurements were corrected for the effect of the blank sample holders (flame sealed Wilmad NMR tube and straw) and eicosane matrix. EPR spectra were measured using an X-band (~9.4 GHz) Bruker EMX Plus spectrometer equipped with an ER4118SMS5 resonator at 5 K. Powdered samples were loaded into 3.9 mm (OD) quartz tubes in a glovebox under dinitrogen, and the tubes were flame sealed under vacuum prior to experiments. Field corrections were applied to the raw data using Bruker strong pitch (g = 2.0028) as a reference. Elemental microanalyses were carried out by Mr Martin Jennings and Mrs Anne Davies at the Micro Analytical Laboratory, Department of Chemistry, University of Manchester.

Synthesis of [U(NBO^{Dipp})$_3$] (1)

Toluene (20 mL) was added to a mixture of NBO^{Dipp}H (1.22 g, 3.0 mmol) and [U{N($SiMe_3$)$_2$}$_3$] (0.72 g, 1.0 mmol) and the resultant mixture stirred for two days. Volatiles were removed *in vacuo* to afford a dark purple residue, which was washed with pentane until the washings were colorless (~ 80 mL) to yield **1** as an analytically pure dark purple solid. Single crystals suitable for X-ray diffraction were grown from a saturated solution of toluene stored at –30 °C overnight. Yield: 0.75 g, 51%. Anal. Calcd for $C_{78}H_{108}B_3N_6O_3U$.0.5toluene: C, 65.51; H, 7.56; N, 5.62%. Found: C, 65.23; H, 7.63; N, 5.65%. ^{1}H NMR (C_7D_8, 298K): δ 9.19 (br, 6H, C*H*=C*H*), 6.00 (br, 12H, *meta*-Ar-*H*), 3.01 (br, 12H,

CH(CH$_3$)$_2$), 1.51 (s, 36H, CH(CH$^a_3$)(CH$^b_3$)), –0.52 (br, 6H, *para*-Ar-*H*), –1.30 (br, 36H, CH(CH$^a_3$)(CH$^b_3$)) ppm. FTIR *v*/cm^{-1}: 2960 (m), 2925 (w), 2866 (w), 1443 (m), 1374 (s), 1273 (w), 1256 (w), 1117 (w), 1073 (w), 974 (w), 881 (w), 804 (m), 763 (s), 747 (w), 728 (w), 707 (w), 687 (w), 648 (s), 601 (w), 492 (w), 418 (w).

Synthesis of [U(NBO^{Dipp})$_2$(Cl)$_2$(THF)$_2$] (2)

NBO^{Dipp}K (1.33 g, 3.0 mmol) in THF (30 mL) was added dropwise to a cold (–78 °C) solution of UCl$_4$ (0.38 g, 1.0 mmol) in THF (30 mL) over five minutes. The green solution was allowed to warm to room temperature before being stirred for 16 hours, after which there was a color change to brown. Volatiles were removed *in vacuo* to afford a brown residue, which was washed with pentane (2 × 30 mL) yielding a light green solid. Soluble residues were then extracted into toluene (40 mL) and filtered, and volatiles were removed *in vacuo* to yield **2** as an analytically light green solid. Single crystals suitable for X-ray diffraction were grown from a saturated solution of pentane stored at –30 °C overnight. Yield: 0.51 g, 80%. Anal. Calcd for $C_{60}H_{88}B_2Cl_2N_4O_4U$: C, 57.20; H, 7.04; N, 4.45%. Found: C, 57.10; H, 7.16; N, 4.34%. ^{1}H NMR (C$_6$D$_6$, 298 K) δ 45.23 (s, 8H, CH(CH$_3$)$_2$), 38.50 (s, 4H, CH=CH), 12.46 (s, 24H, CH(CH$^a_3$)(CH$^b_3$)), 5.74 (d, ^3J = 10.7 Hz, 8H, *meta*-Ar-*H*), 3.42 (m, 4H, *para*-Ar-*H*), –3.50 (s, 24H, CH(CH$^a_3$)(CH$^b_3$)), –29.48 (s, 8H, THF), –71.13 (s, 8H, THF) ppm. FTIR *v*/cm^{-1}: 3069 (w), 2962 (m), 2866 (m), 1457 (m), 1442 (m), 1411 (w), 1368 (s), 1349 (w), 1300 (s), 1276 (m), 1254 (w), 1190 (m), 1162 (w), 1120 (m), 1100 (w), 1077 (w), 1061 (m), 10467 (w), 1006 (m), 975 (m), 932 (w), 917 (w), 895 (w), 881 (m), 834 (m), 805 (s), 777 (s), 752 (s), 708 (s), 647 (s), 592 (w), 576 (w), 536 (w), 491 (w), 448 (w), 436 (w), 442 (w).

Synthesis of [U(NBO^{Dipp})$_3$(Cl)(THF)] (3)

NBO^{Dipp}K (6.65 g, 15.0 mmol) in THF (50 mL) was added dropwise to a cold (–78 °C) solution of UCl$_4$ (1.90 g, 5.0 mmol) in THF (50 mL) over five minutes. The green solution was allowed to warm to room temperature then stirred for 16 hours, after which there was a color change to brown. Volatiles

were removed *in vacuo* to afford a brown residue. Toluene (80 mL) was added, and the resultant brown solution refluxed at 110 °C for 16 hours. After cooling, volatiles were removed *in vacuo* to afford a dark brown residue, which was washed with pentane (100 mL) yielding a green solid. Soluble residues were then extracted into toluene (40 mL) and filtered, and volatiles were removed *in vacuo* to yield **3** as an analytically pure green/yellow solid. Single crystals suitable for X-ray diffraction were grown from a saturated solution of pentane stored at –30 °C overnight. Yield: 5.54 g, 71%. Anal. Calcd for $C_{82}H_{116}B_3ClN_6O_4U$: C, 63.31; H, 7.52; N, 5.4%. Found: C, 63.46; H, 7.65; N, 5.19%. ^1H NMR (C_6D_6, 298 K) δ 12.46 (s, 6H, CH=C*H*), 9.15 (s, 12H, C*H*(CH_3)$_2$), 4.34 (d, ^3J= 7.8 Hz, 12H, *meta*-Ar-*H*), 4.12 (t, ^3J= 7.8 Hz, 6H, *para*-Ar-*H*), 2.72 (d, ^3J= 6.6 Hz, 36H, CH(CH^a_3)(CH^b_3)), –2.79 (s, 36H, CH(CH^a_3)(CH^b_3)), –13.35 (s, 4H, THF), –19.30 (s, 4H, THF) ppm. FTIR *v*/cm^{-1}: 2962 (m), 2868 (m), 1576 (w), 1458 (m), 1383 (s), 1353 (s), 1292 (w), 1275 (m), 1255 (m), 1226 (w), 1192 (w), 1159 (w), 1116 (m), 1076 (m), 1057 (w), 1013 (w), 972 (m), 934 (w), 891 (m), 859 (m), 804 (w), 767 (w), 747 (s), 708 (s), 648 (s), 595 (w), 580 (w), 551 (w), 530 (w), 491 (w), 453 (w), 429 (w), 410 (w).

Synthesis of [U(NBO^{Dipp})$_3$(NAd)] (4)

At –78 °C, toluene (20 mL) was added to a mixture of **1** (1.45 g, 1.0 mmol) and AdN_3 (0.12 g, 1.0 mmol), resulting in immediate gas evolution and formation of a dark purple suspension. The resultant mixture was stirred for two days. Volatiles were removed *in vacuo* to afford a dark brown residue, which was washed with pentane (3 × 10 mL) yielding **4** as an analytically pure dark brown solid. Single crystals suitable for X-ray diffraction were grown from a saturated solution of toluene stored at –30 °C overnight. Yield: 1.04 g, 65%. Anal. Calcd for $C_{88}H_{123}B_3N_7O_3U$: C, 66.17; H, 7.76; N, 6.14%. Found: C, 66.65; H, 8.20; N, 5.80%. ^1H NMR (C_7D_8, 298 K) δ 16.26 (s, 6H), 14.91 (s, 12H, *meta*-Ar-*H*), 9.28 (s, 6H), 9.14 (s, 6H), 8.60 (s, 12H, C*H*(CH_3)$_2$), 6.98 (s, 36H, CH(CH^a_3)(CH^b_3)), 3.12 (s, 3H, Ad-*H*), 3.12 (s, 6H, C*H*=C*H*), –0.27 (s, 36H, CH(CH^a_3)(CH^b_3)) ppm. FTIR *v*/cm^{-1}: 2959 (w), 2927 (w), 2898 (w), 2870 (w), 2845 (w), 1452 (m), 1420 (w), 1381 (m), 1342 (s), 1307 (w), 1289

(w), 1276 (w), 1255 (w), 1225 (w), 1187 (w), 1147 (w), 1114 (w), 1074 (w), 1056 (w), 972 (w), 936 (w), 882 (w), 807 (m), 744 (s), 707 (m), 648 (m), 593 (w), 532 (w), 498 (w), 469 (w), 425 (w).

Computational Details

General

Geometry optimizations for **1-4** were performed using coordinates derived from their crystal structures as the starting points. No constraints were imposed on the structures during the geometry optimizations.

DFT Calculations

The calculations were performed using the Amsterdam Density Functional (ADF) suite version 2017 with standard convergence criteria.[12,13] The DFT geometry optimizations employed Slater type orbital (STO) triple-ζ-plus polarization all-electron basis sets (from the Dirac and ZORA/TZP database of the ADF suite). Scalar relativistic approaches (spin-orbit neglected) were used within the ZORA Hamiltonian[14-16] for the inclusion of relativistic effects and the local density approximation (LDA) with the correlation potential due to Vosko *et al* was used in all of the calculations.[17] Generalized gradient approximation (GGA) corrections were performed using the functionals of Becke and Perdew.[18,19] Dispersion effects were explicitly treated for **3** and **4** with Grimme's D3 with BJ damping corrections,[20] but not for **1** and **2** as this led to poorer agreement of experimental and computational uranium-ligand bond lengths. Analytical frequency calculations were carried out within the ADF program. Natural Bond Order (NBO) and Natural Localized Molecular Orbital (NLMO) analyses were carried out with NBO 6.0.19.[21] The Quantum Theory of Atoms in Molecules analysis[22,23] was carried out within the ADF program. We quote Nalewajski-Mrozek bond orders since they reproduce expected bond multiplicities reliably in polar heavy atom structures whereas Mayer bond orders for polar bonds often do not conform with chemical intuition.[24] The ADF-GUI (ADFview) was used to prepare the three-dimensional plots of the electron density. In all cases,

Aufbau formulations were found with the appropriate spin formulations of $5f^3$ uranium(III) (quartet), $5f^2$ uranium(IV) (triplet), and $5f^1$ uranium(V) (doublet).

Experimental fitting of magnetic data

Variable-temperature molar magnetic susceptibility between 1.8 K and 300 K and the energies of the 5f– 5f transitions were modelled using the program CONDON 3.0.[25,26] Fitting was performed in C_{3v} symmetry. Starting values of the crystal field parameters and spin-orbit coupling were varied until the fit could not be improved. The full composition of the states was obtained by inserting the best fit parameters in the program F-shell.[27] Since the program outputs the composition in the $|L,S,J,m_J\rangle$ basis, a change of projection using the Clebsch Gordan coefficients into the base $|L,S,m_l,m_s\rangle$ was performed.

Figures

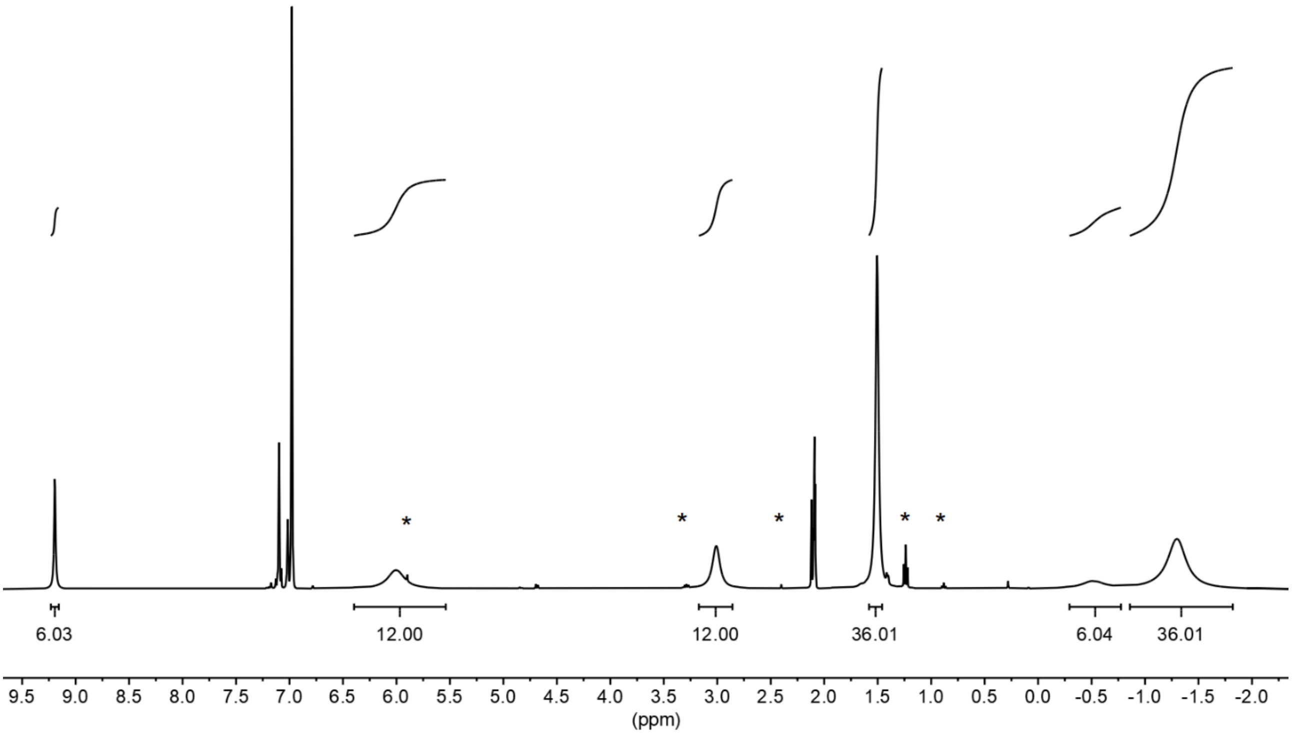

Figure S1. ^{1}H NMR spectrum of **1** in C_7D_8. The asterisks (*) denote trace solvent impurities: n-pentane (δ ~1.23 (m), 0.87 (t) ppm) and a residual impurity of NBO^{Dipp}H.

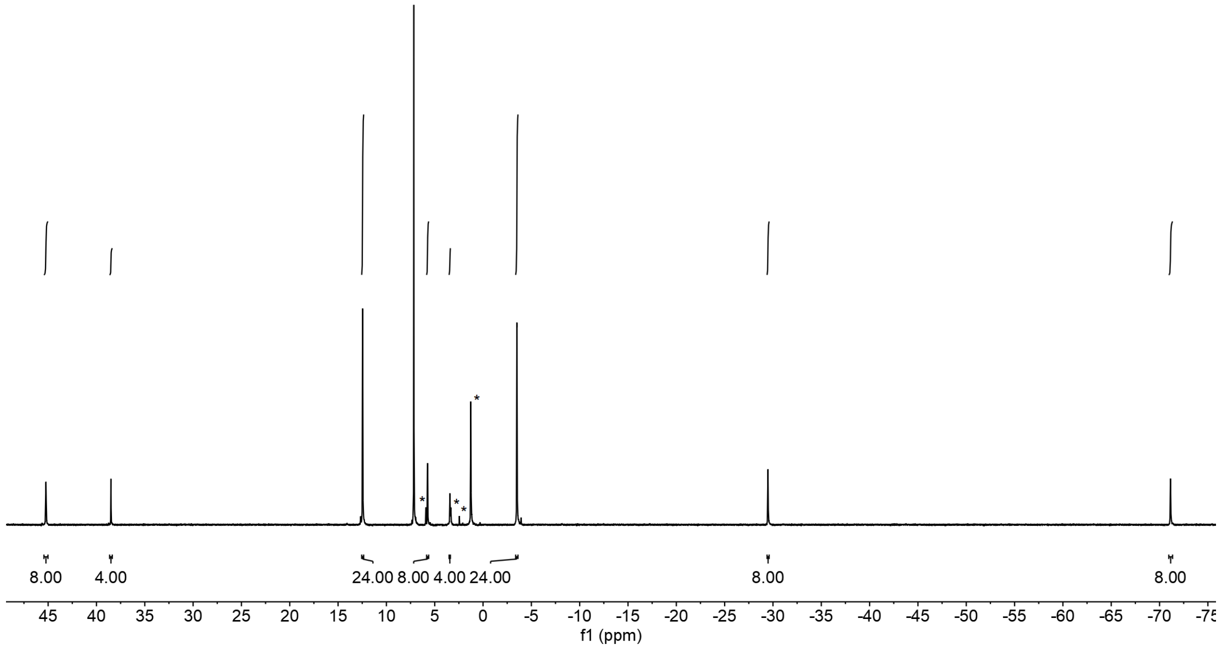

Figure S2. ^{1}H NMR spectrum of **2** in C_6D_6. The asterisks (*) denote a residual impurity of NBO^{Dipp}H.

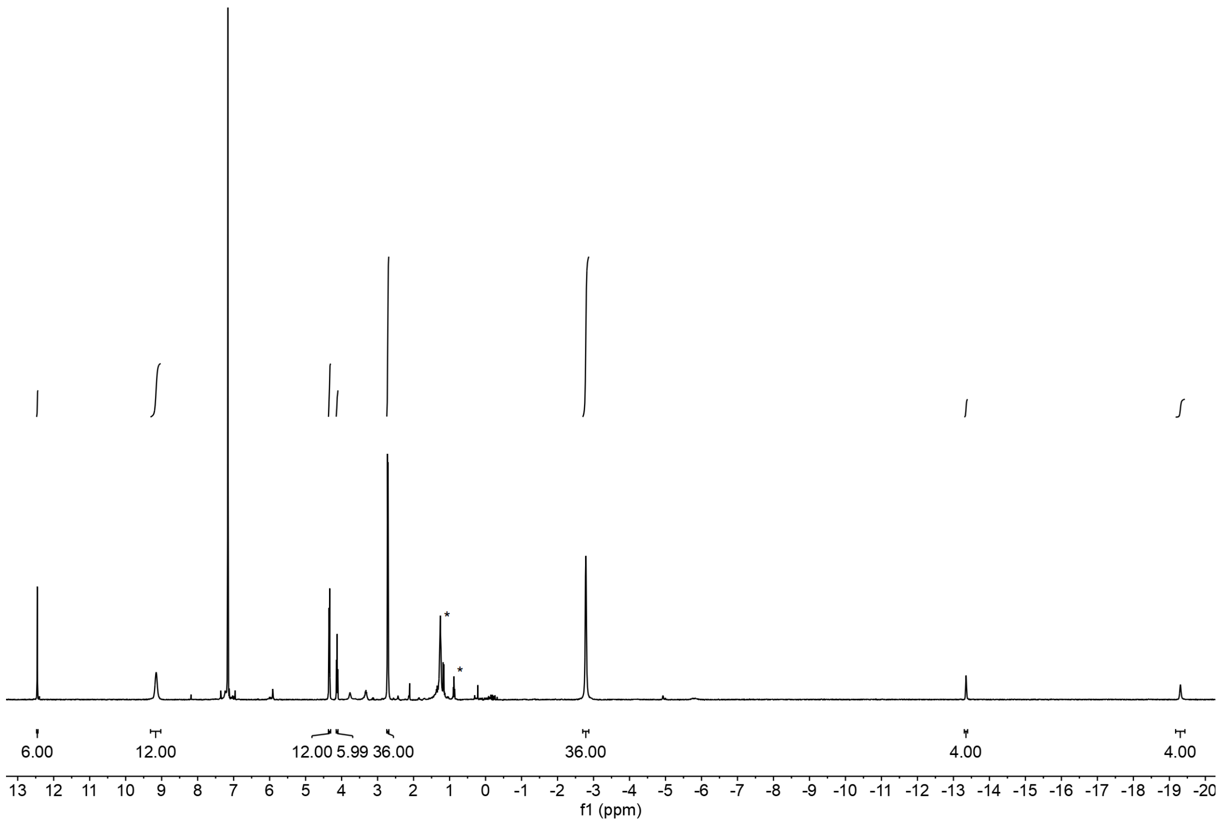

Figure S3. ^{1}H NMR spectrum of **3** in C_6D_6. The asterisks (*) denote trace solvent impurities: n-pentane (δ ~1.23 (m), 0.87 (t) ppm).

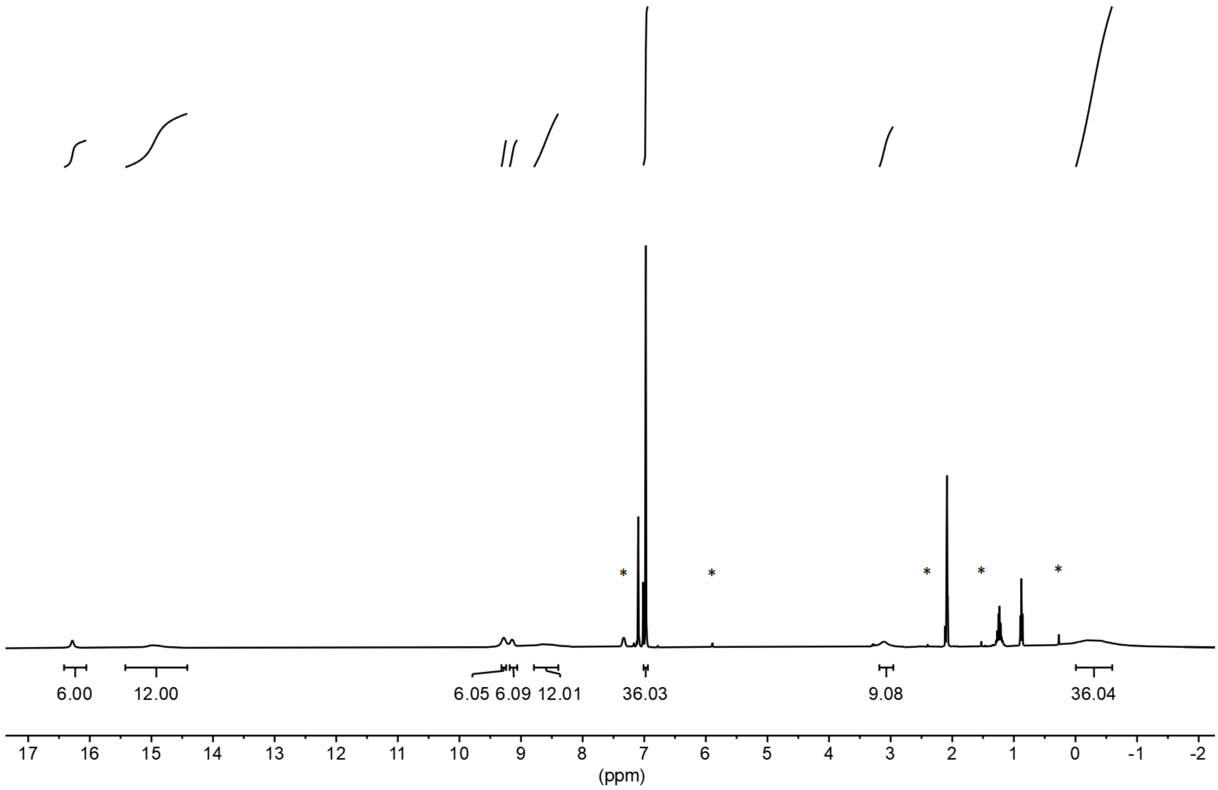

Figure S4. ^{1}H NMR spectrum of **4** in C_7D_8. The asterisks (*) denote a residual impurity of NBO^{Dipp}H and silicon grease.

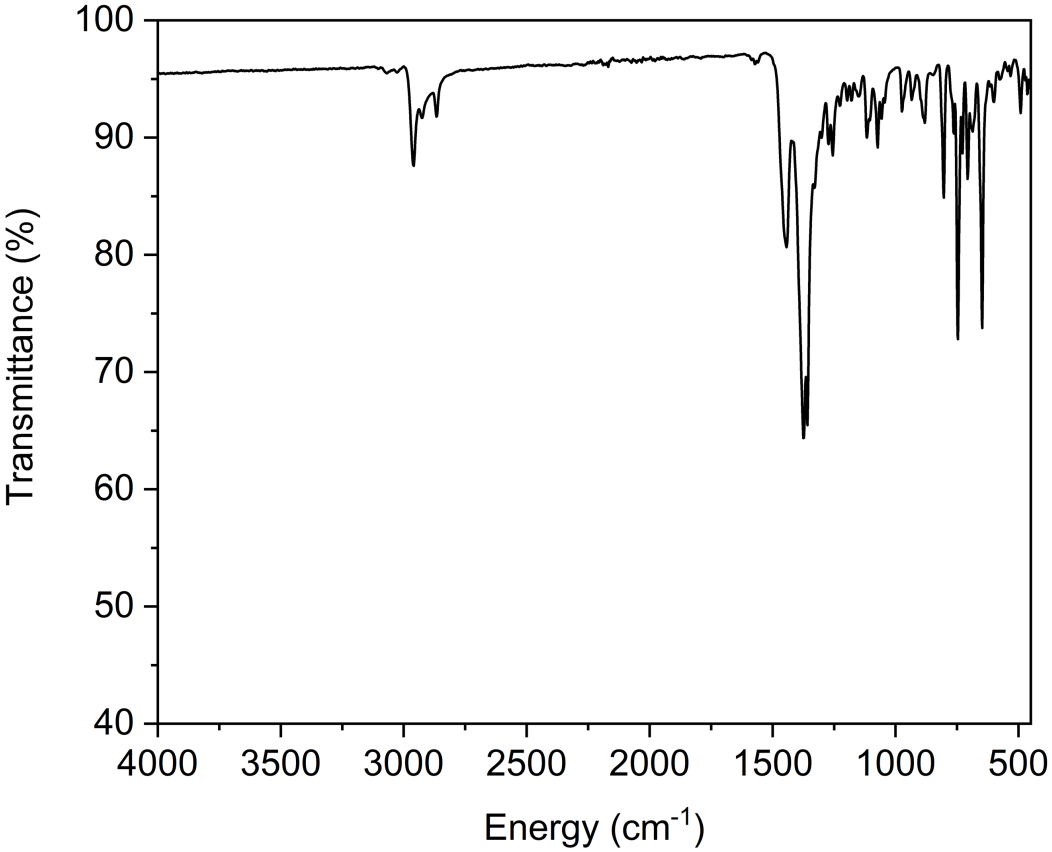

Figure S5. ATR-IR spectrum of **1**.

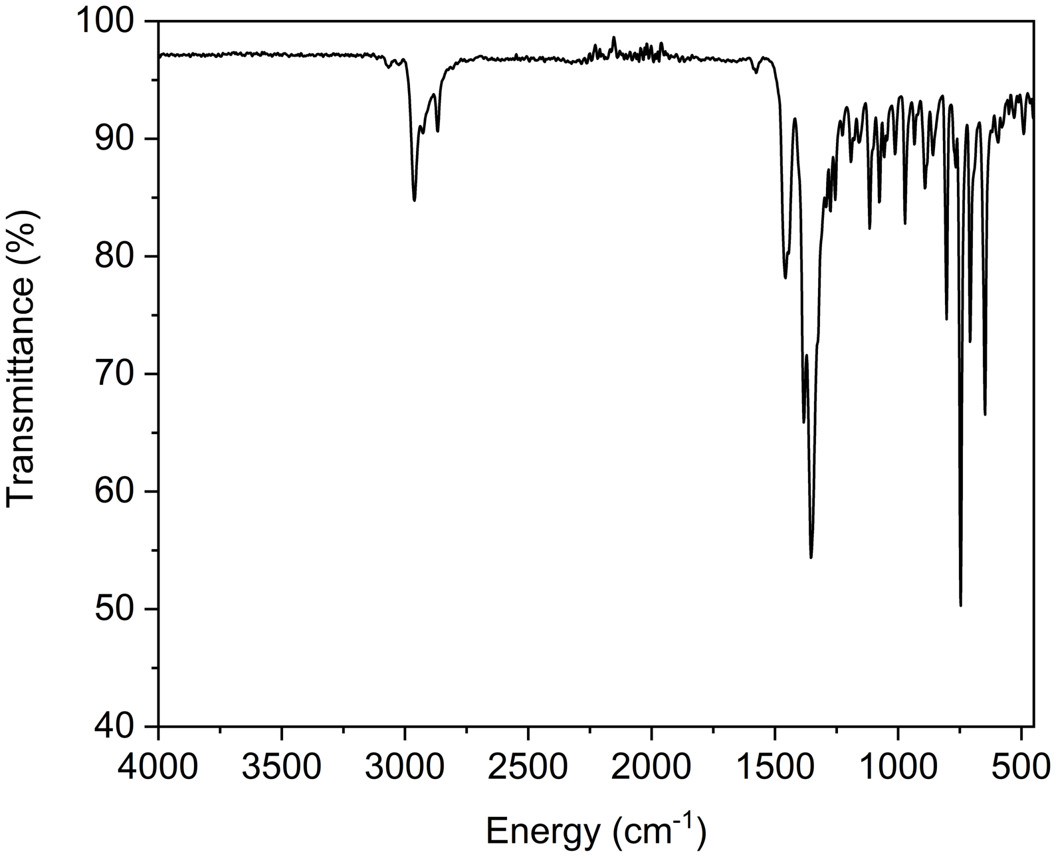

Figure S6. ATR-IR spectrum of **2**.

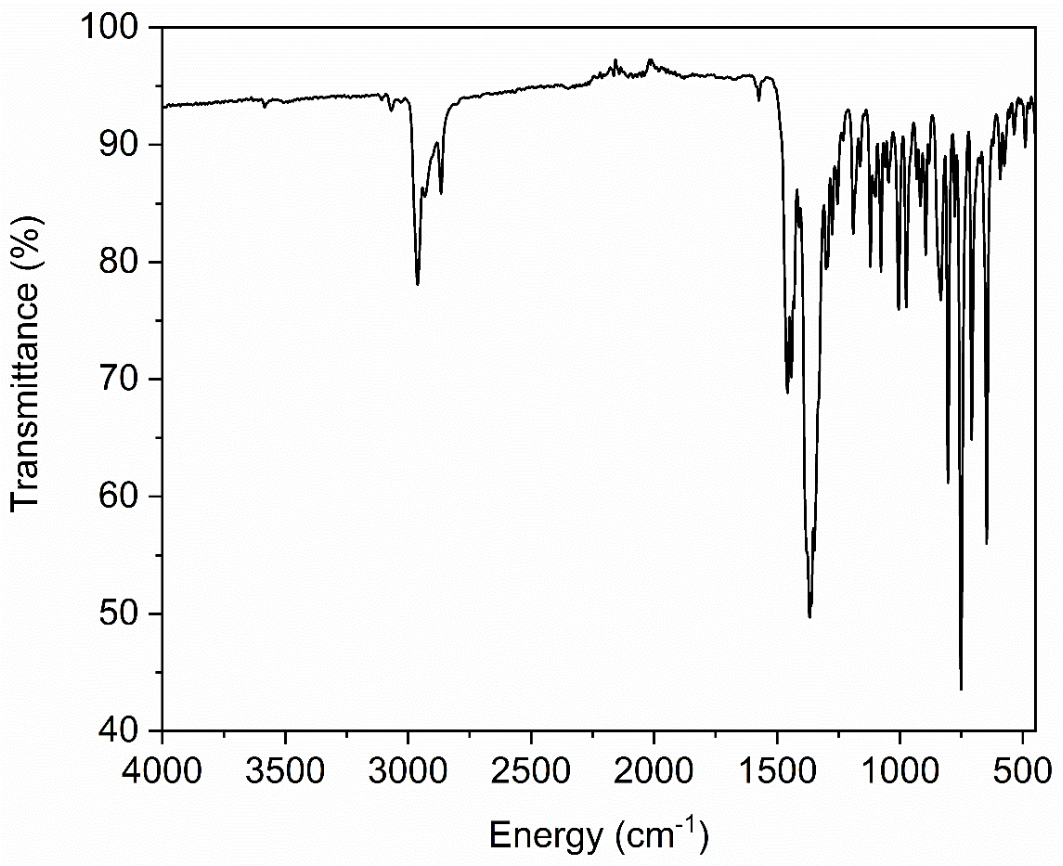

Figure 7. ATR-IR spectrum of **3**.

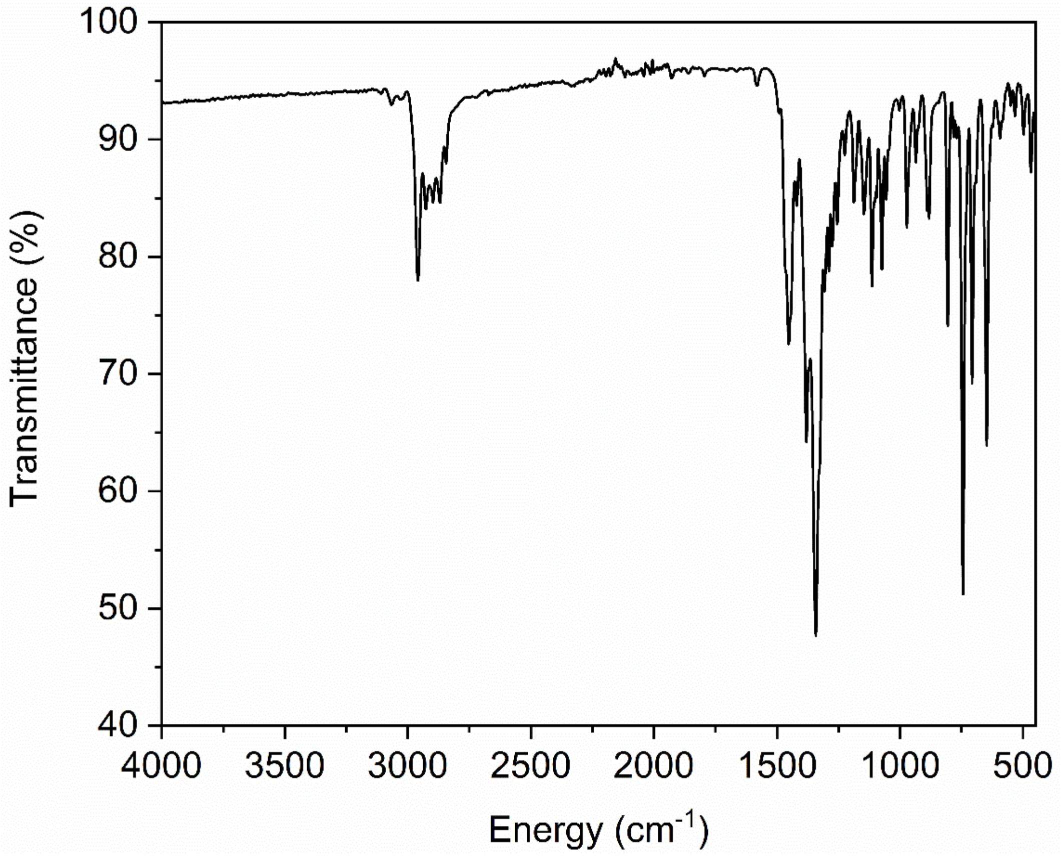

Figure S8. ATR-IR spectrum of **4**.

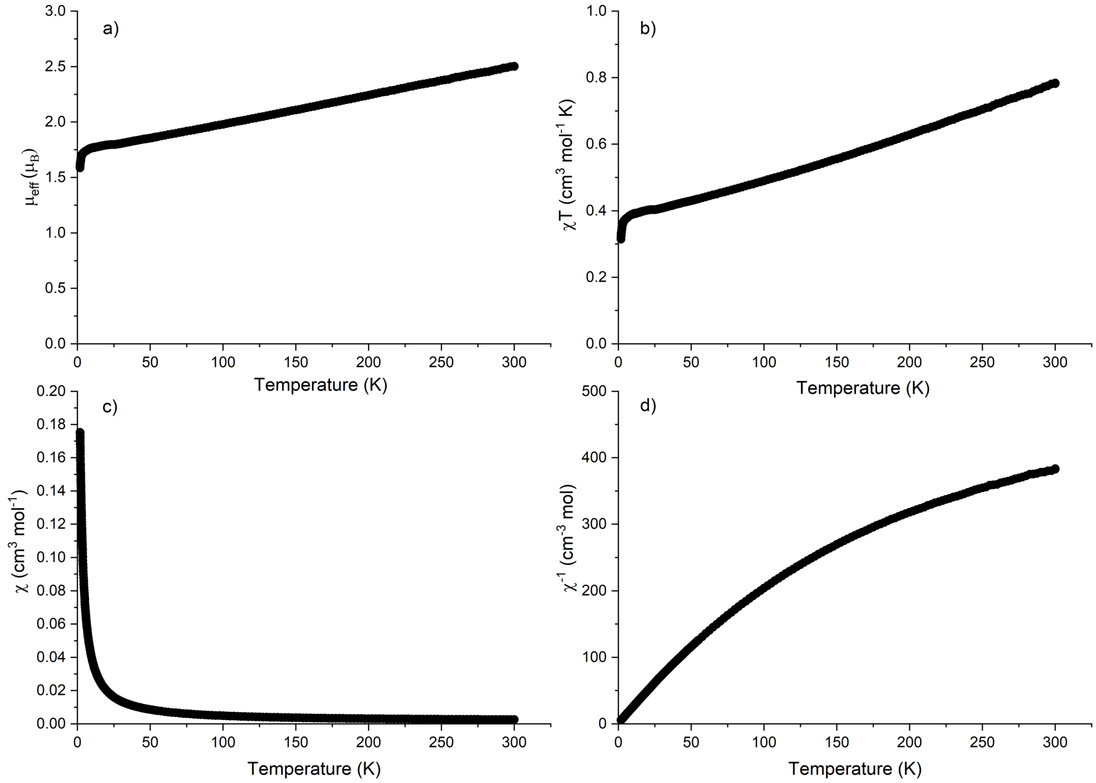

Figure S9. Variable-temperature SQUID magnetometry of **1** over the temperature range 1.8-300 K in an external 0.1 T field: a) μ_{eff} vs T; b) $\chi_M T$ vs T; c) χ_M vs T; d) χ_M^{-1} vs T. Line is a guide only.

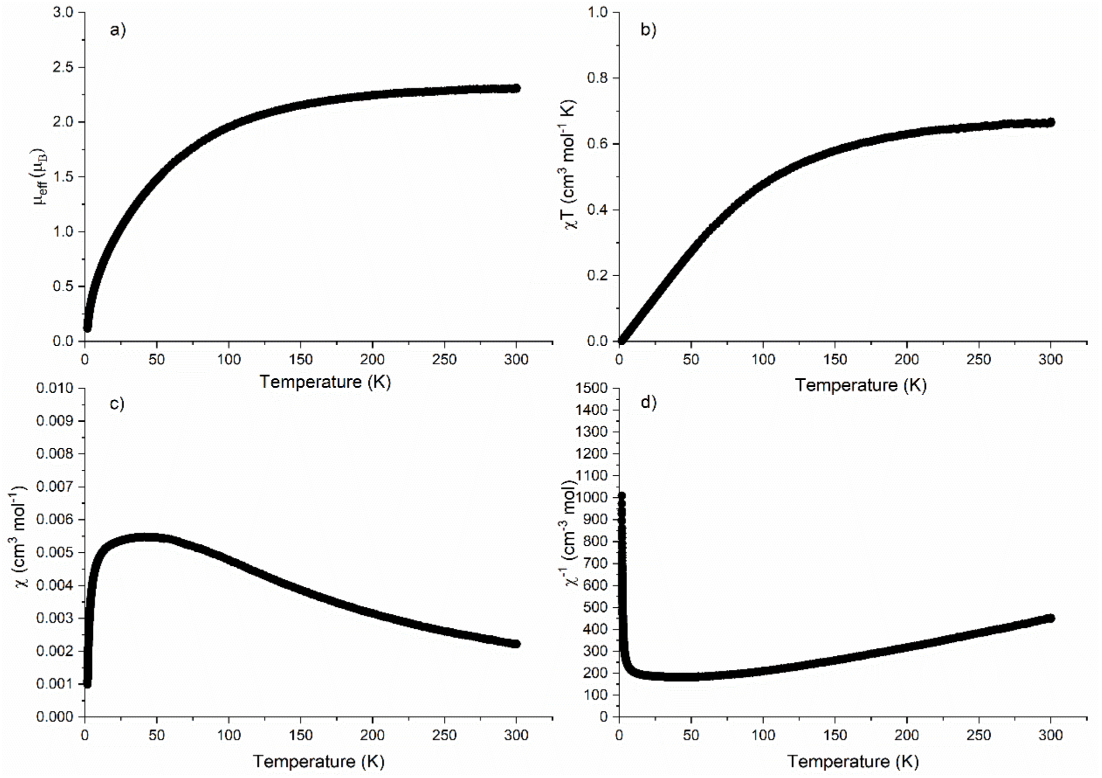

Figure S10. Variable-temperature SQUID magnetometry of **2** over the temperature range 1.8-300 K in an external 0.1 T field: a) μ_{eff} vs T; b) $\chi_M T$ vs T; c) χ_M vs T; d) χ_M^{-1} vs T. Line is a guide only.

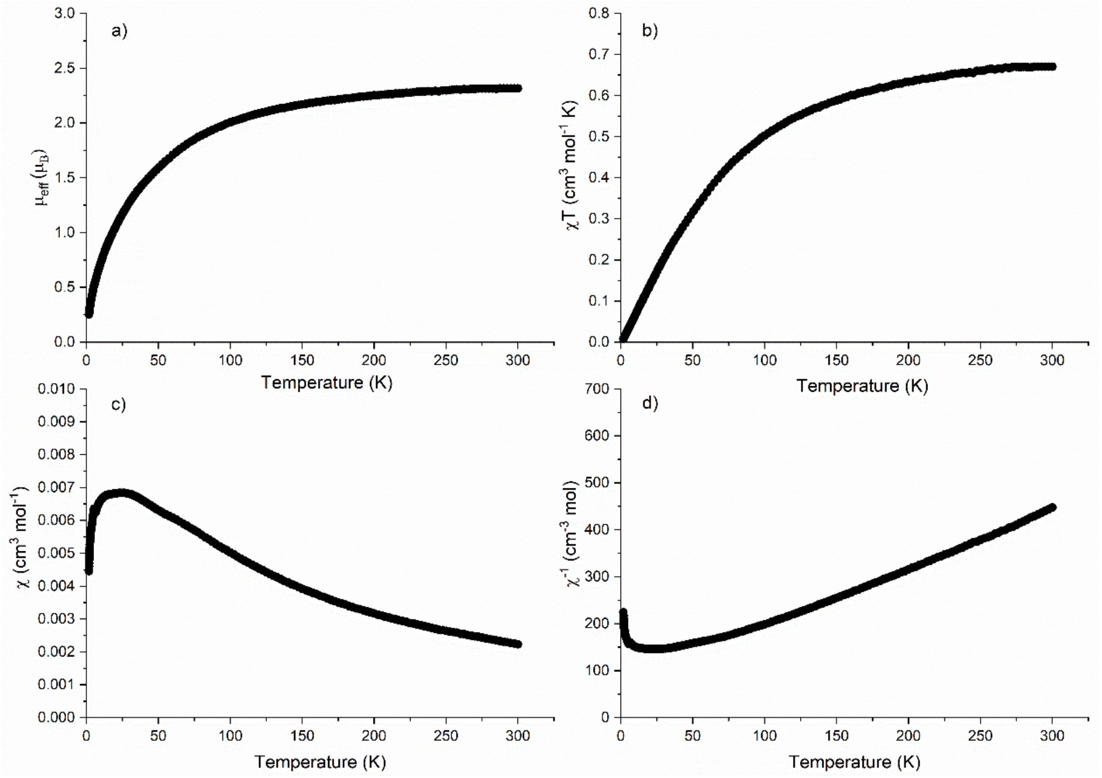

Figure S11. Variable-temperature SQUID magnetometry of **3** over the temperature range 1.8-300 K in an external 0.1 T field: a) μ_{eff} vs T; b) $\chi_M T$ vs T; c) χ_M vs T; d) χ_M^{-1} vs T. Line is a guide only.

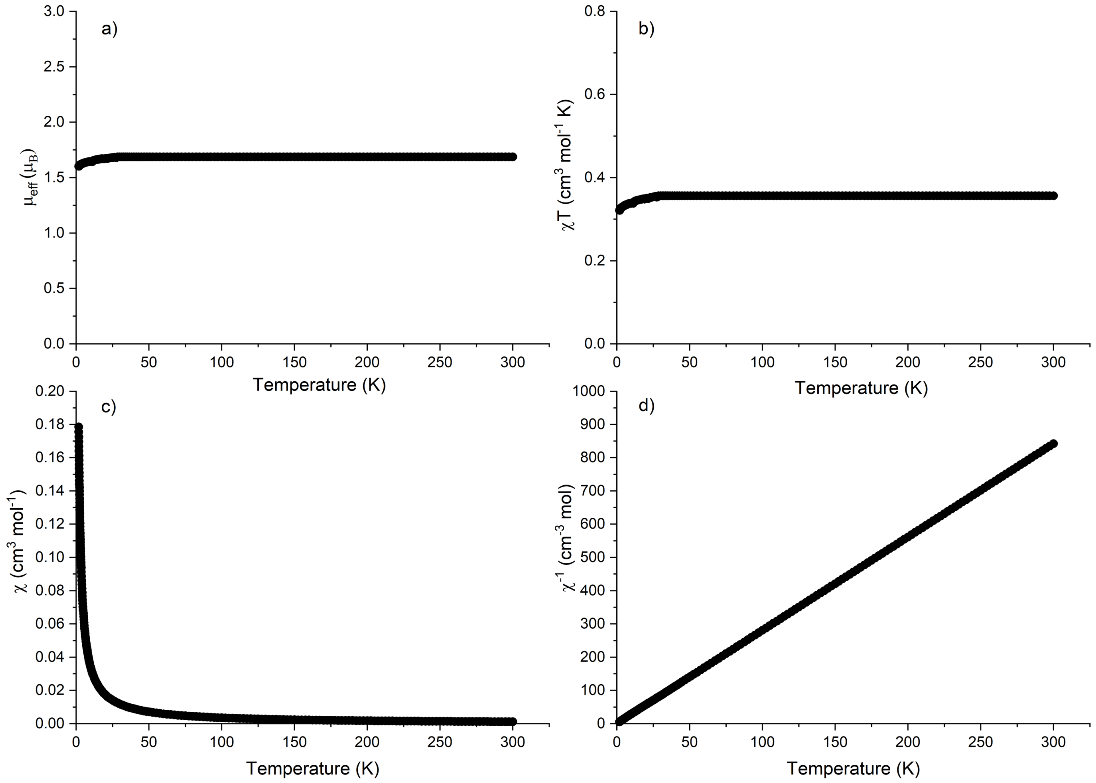

Figure S12. Variable-temperature SQUID magnetometry of **4** over the temperature range 1.8-300 K in an external 0.1 T field: a) μ_{eff} vs T; b) $\chi_M T$ vs T; c) χ_M vs T; d) χ_M^{-1} vs T. Line is a guide only.

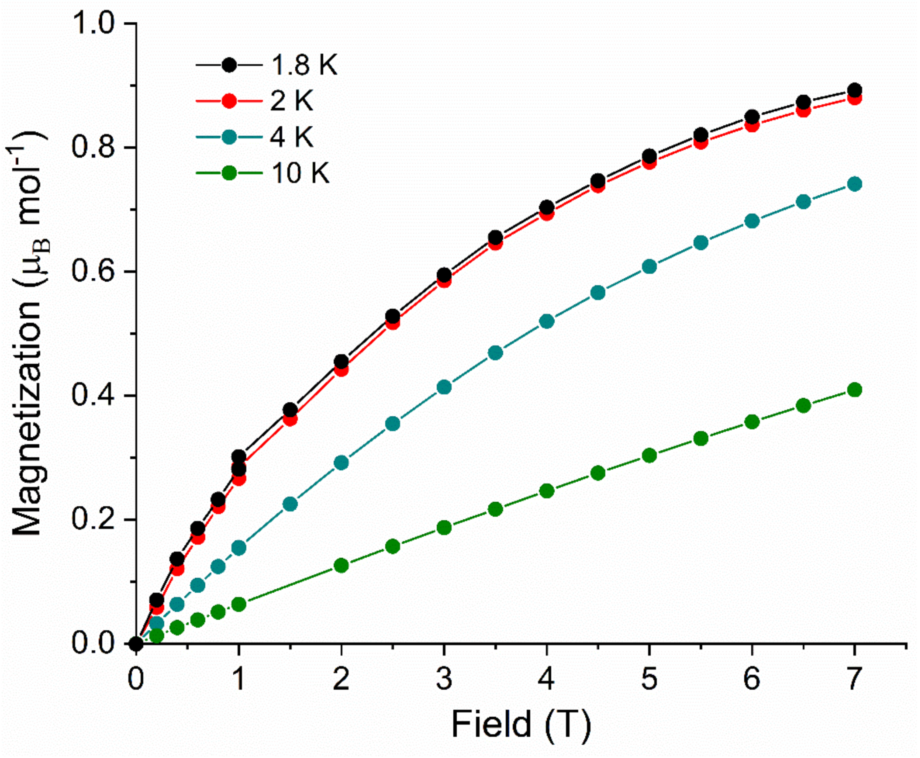

Figure S13. Isothermal Magnetization vs Field data for **1** at 1.8 (black), 2 (red), 4 (cyan), and 10 (green) K. Lines are a guide to the eye only.

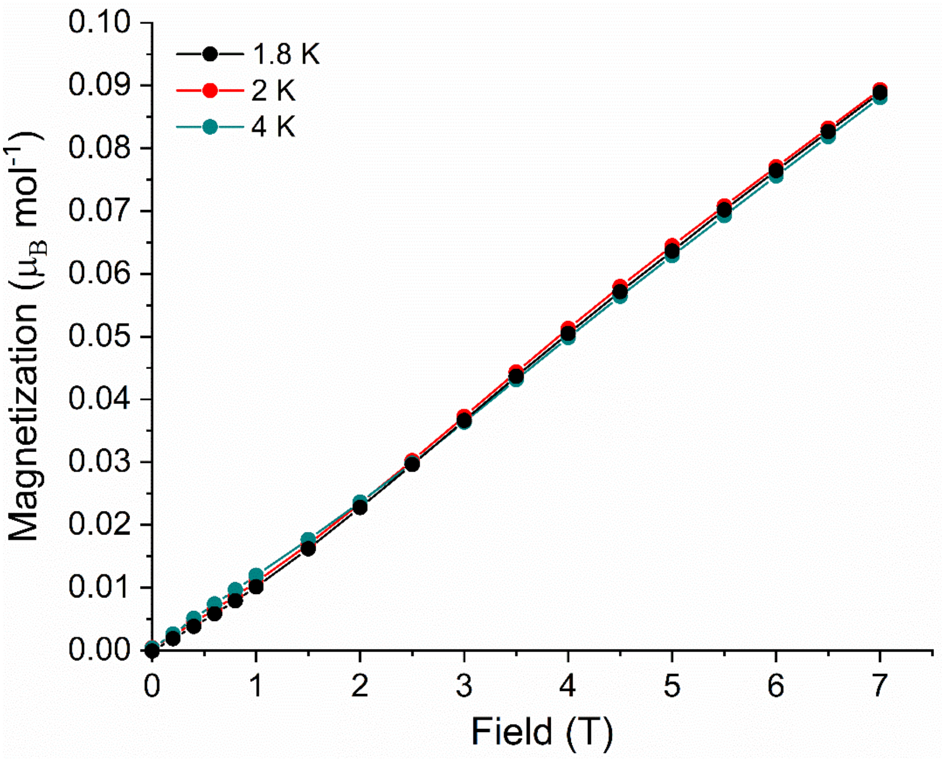

Figure S14. Isothermal Magnetization vs Field data for **2** at 1.8 (black), 2 (red), and3 4 (cyan) K. Lines are a guide to the eye only.

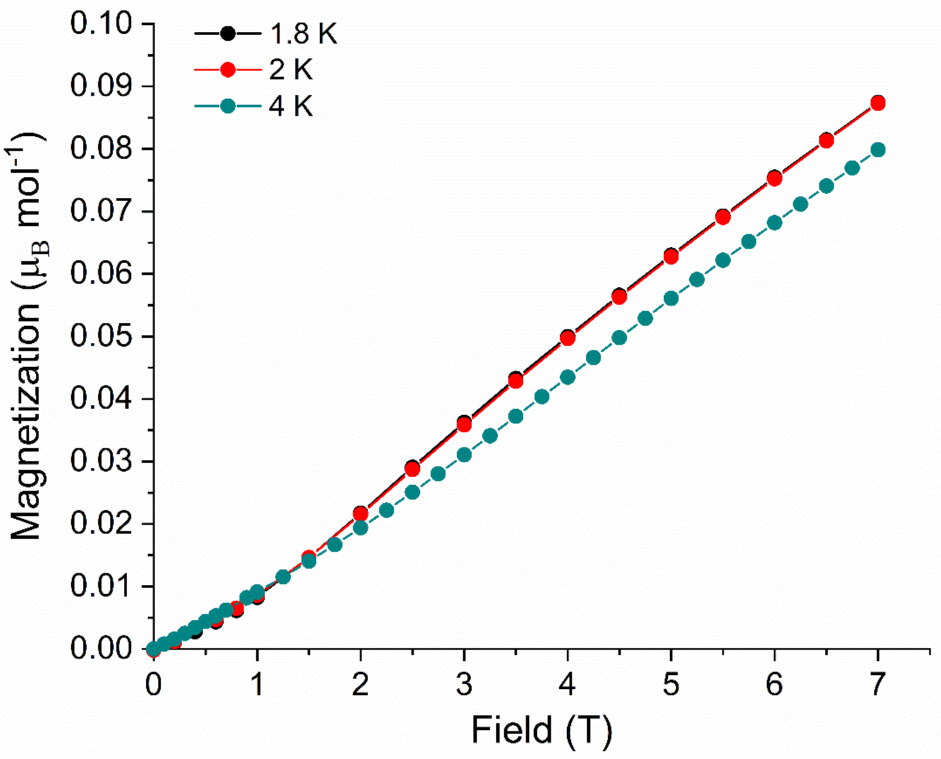

Figure S15. Isothermal Magnetization vs Field data for **3** at 1.8 (black), 2 (red), and 4 (cyan) K. Lines are a guide to the eye only.

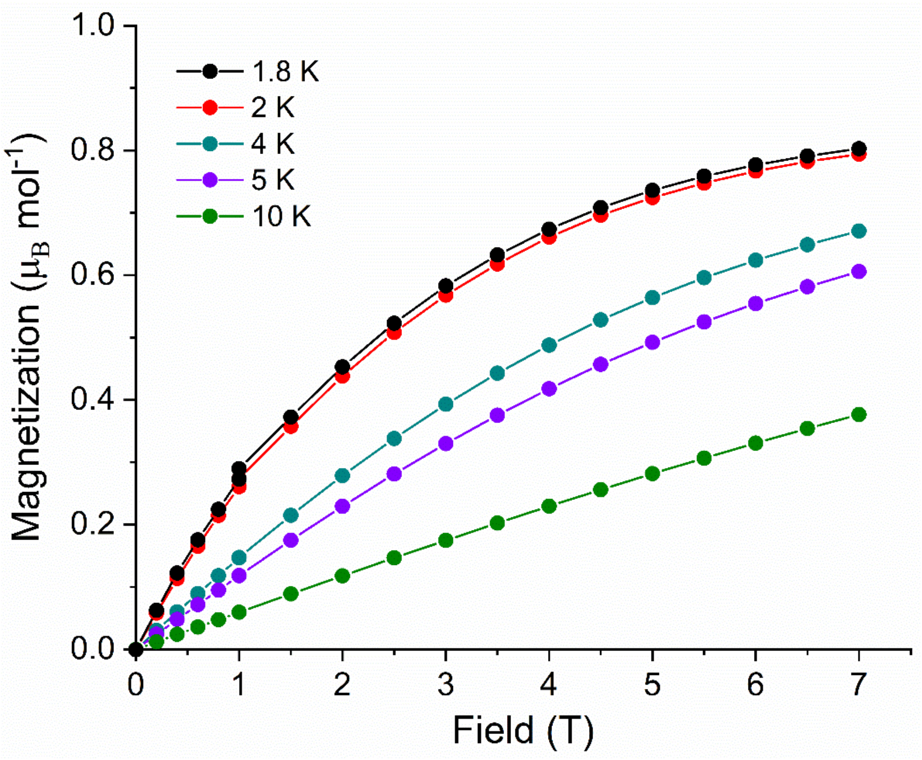

Figure S16. Isothermal Magnetization vs Field data for **4** at 1.8 (black), 2 (red), 4 (cyan), 5 (purple), and 10 (green) K. Lines are a guide to the eye only.

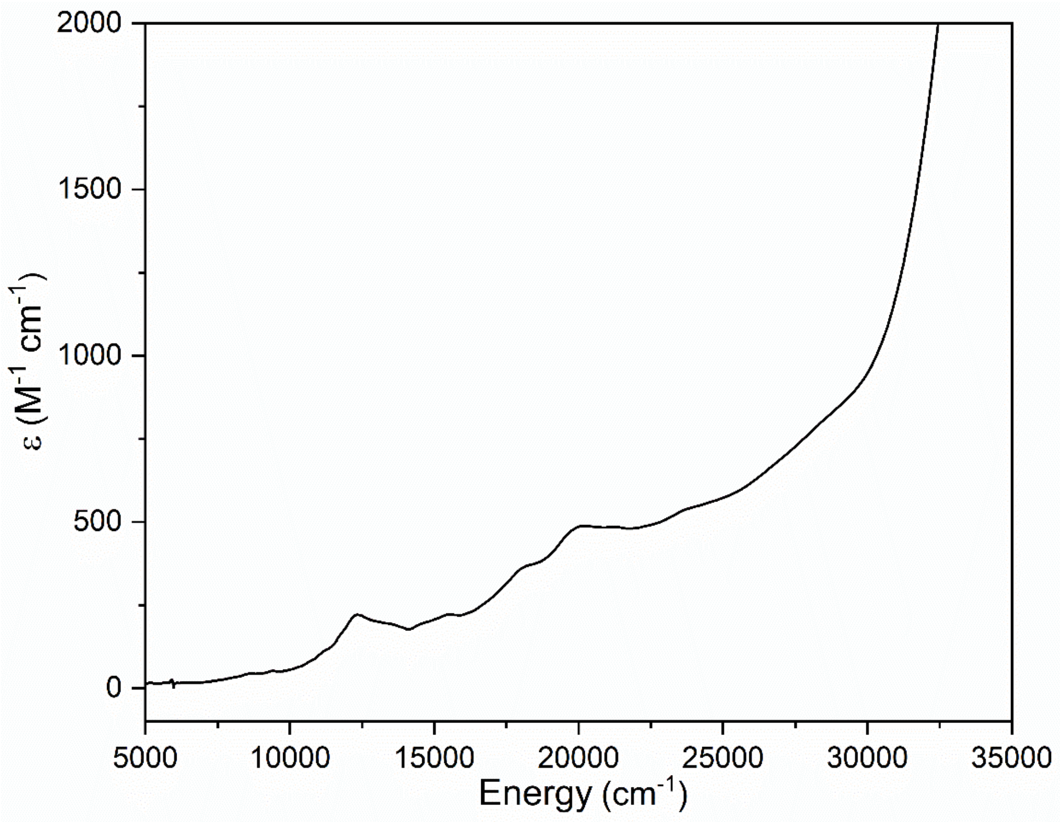

Figure S17. Solution UV/Vis/NIR spectrum of **1** (1 mM, toluene) over the range 5,000-35,000 cm^{-1}.

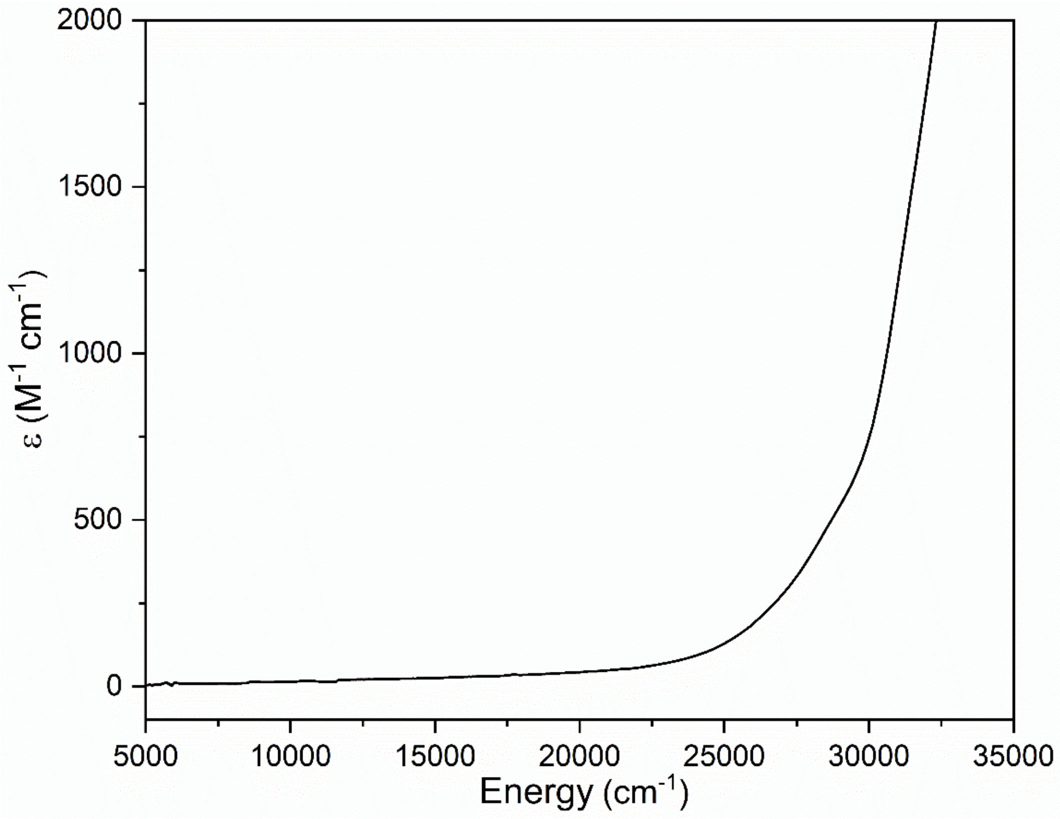

Figure S18. Solution UV/Vis/NIR spectrum of **2** (1 mM, THF) over the range 5,000-35,000 cm^{-1}.

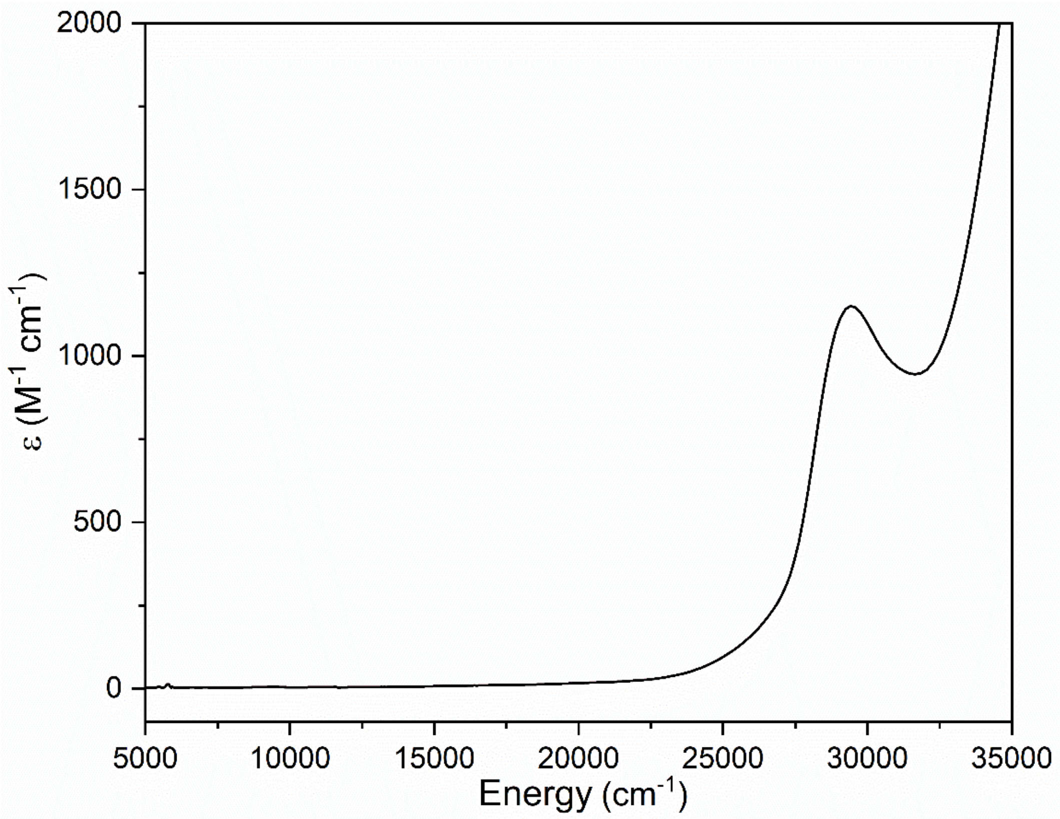

Figure S19. Solution UV/Vis/NIR spectrum of **3** (1 mM, THF) over the range 5,000-35,000 cm^{-1}.

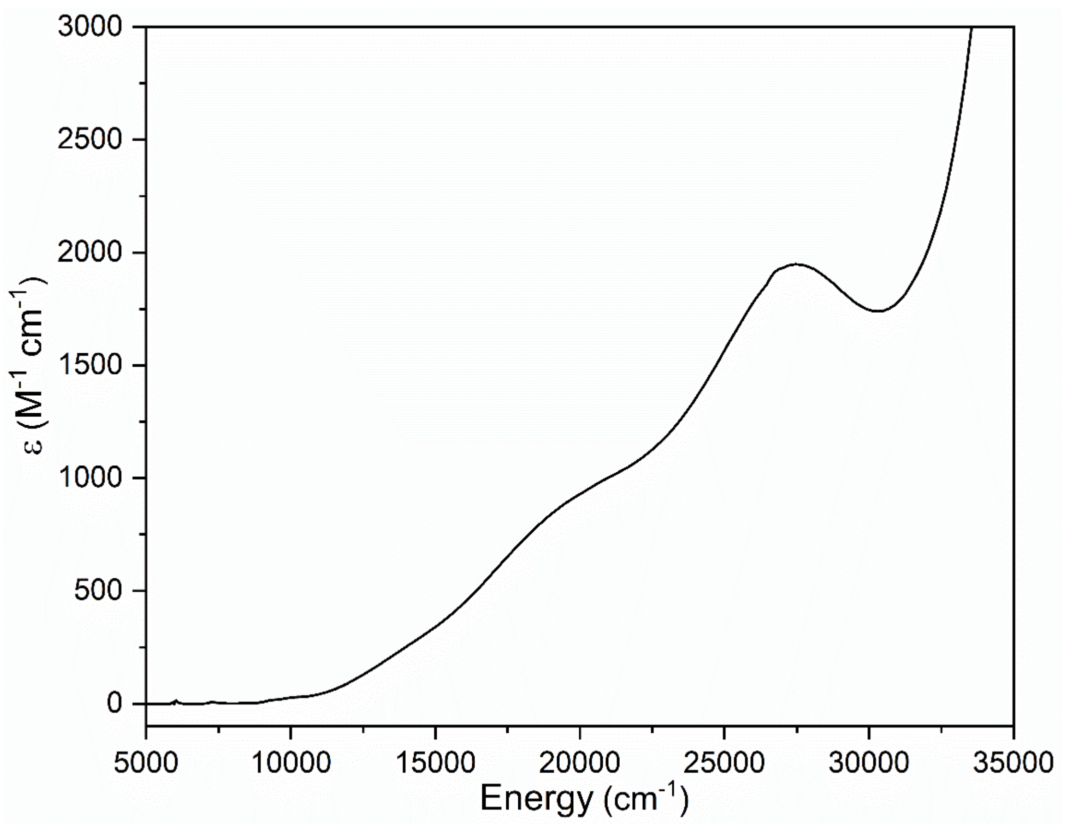

Figure S20. Solution UV/Vis/NIR spectrum of **4** (1 mM, toluene) over the range 5,000-35,000 cm^{-1}.

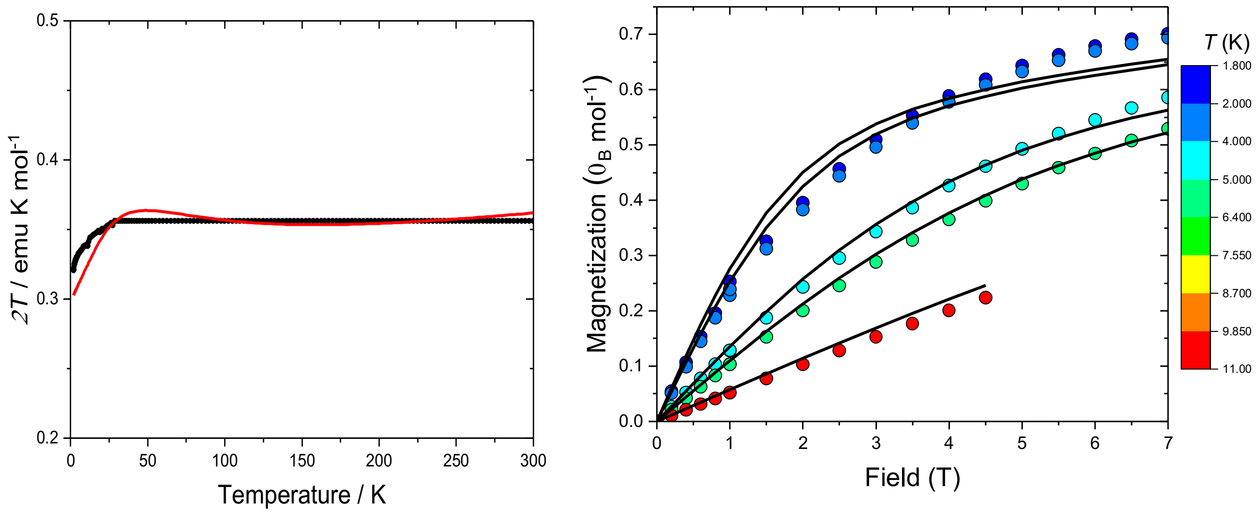

Figure S21. Plots of χT vs T (experimental data, black dots; fit, red line) in an external 0.1 T field and Magnetization vs Field (experimental data, dots, fit, black lines) for **4** with fits derived from CONDON 3.0.

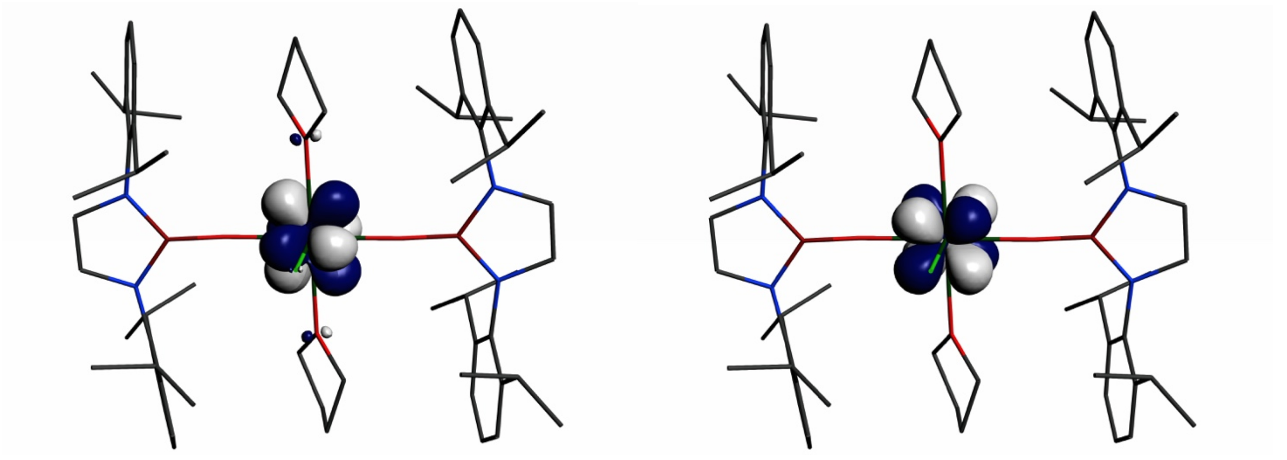

Figure S22. Selected molecular orbitals for **2**. Left to right: HOMO (323a, −2.996 eV) and HOMO−1 (322a, −3.140 eV). Hydrogen atoms are omitted for clarity.

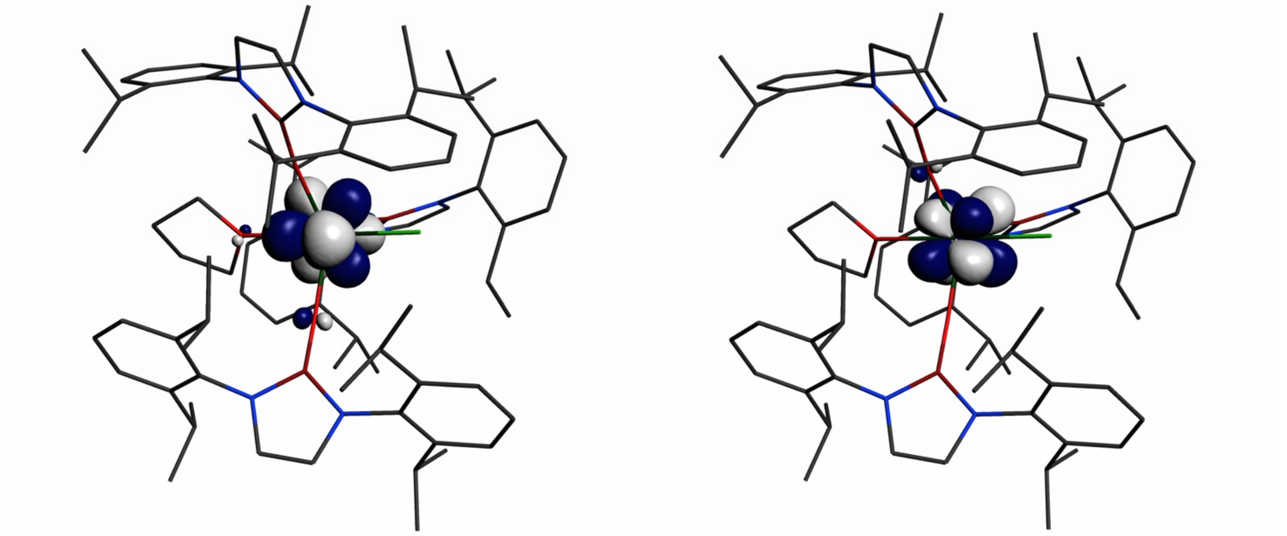

Figure S23. Selected molecular orbitals for **4**. Left to right: HOMO (404a, −2.962 eV) and HOMO−1 (403a, −3.035 eV). Hydrogen atoms are omitted for clarity.

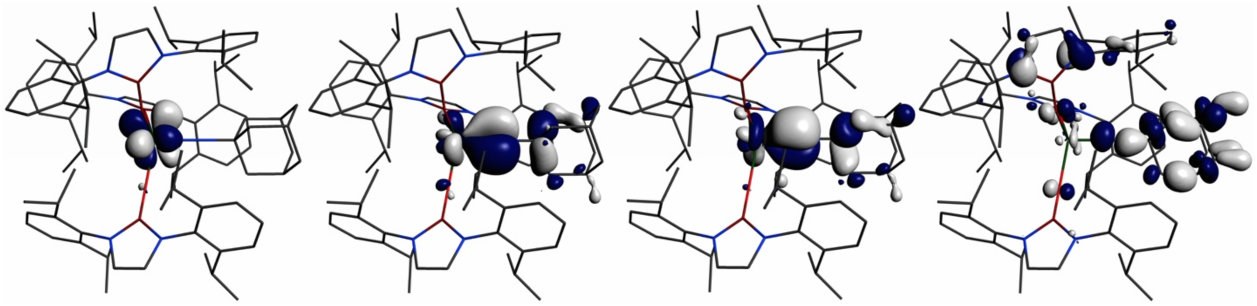

Figure 24. Selected molecular orbitals for **4**. Left to right: HOMO (416a, −3.273 eV), HOMO−4 (412a, −4.937 eV), HOMO−5 (411a, −5.009 eV), HOMO−21 (396a, −6.188 eV). Hydrogen atoms are omitted for clarity.

Tables

Table S1. Crystallographic data for 1-4.

	**1**	**2**	**3**	**4**
Formula	$C_{81.50}H_{112}B_3N_6O_3U$	$C_{60}H_{88}B_2Cl_2N_4O_4U$	$C_{82}H_{116}B_3ClN_6O_4U$	$C_{105.50}H_{143}B_3N_7O_3U$
Fw, g mol^{-1}	1494.22	1259.89	1555.71	1827.72
Cryst size, mm	0.40 × 0.17 × 0.13	0.25 × 0.16 × 0.13	0.40 × 0.27 × 0.26	0.15 × 0.10 × 0.10
Crystal system	triclinic	monoclinic	monoclinic	triclinic
Space group	*P*-1	*C*2/c	$P2_1$/n	*P*-1
Temperature (K)	100(2)	120(2)	150(2)	120(2)
a, (Å)	12.5329(2)	27.1009(3)	17.14373(4)	14.1180(3)
b, (Å)	12.6045(2)	12.72220(10)	20.65740(5)	14.1319(3)
c, (Å)	26.7347(3)	17.8934(2)	23.00562(5)	27.3971(3)
α, (°)	81.1620(10)	90	90	84.1980(12)
β, (°)	82.4740(10)	98.2800(10)	91.6161(2)	84.3881(12)
γ, (°)	67.9500(10)	90	90	61.8414(19)
V, ($Å^3$)	3855.52(10)	6105.03(11)	8144.08(3)	4786.67(16)
Z	2	4	4	2
ρ_{calc} g cm^{-3}	1.287	1.371	1.269	1.268
μ, mm^{-1}	6.301	8.644	6.290	5.174
Reflections	43543	29206	130611	19186
Unique reflections, R_{int}	15459, 0.0430	6535, 0.0450	17682, 0.0335	19186, 0.0566
Reflections $F^2 > 2\sigma(F^2)$	15400	5780	17442	17247
Transmission range	0.460-1.000	0.636-1.000	0.161-1.000	0.564-1.000
R, R_w^a ($F^2 > 2\sigma(F^2)$)	0.0393, 0.1002	0.0276, 0.0757	0.0283, 0.0770	0.0672, 0.1779
R, R_w^a (all data)	0.0394, 0.1003	0.0303, 0.0782	0.0285, 0.0772	0.0744, 0.1819
S^a	1.057	1.087	1.083	1.125
Parameters, Restraints	908, 99	339, 9	929, 79	1136, 282
Max., min., e $Å^{-3}$	4.023, –2.195	1.217, –1.323	1.072, –2.211	4.274, –2.745

^{a} Conventional $R = \Sigma||F_o| - |F_c||/\Sigma|F_o|$; $R_w = [\Sigma w(F_o^2 - F_c^2)^2/\Sigma w(F_o^2)^2]^{1/2}$;
$S = [\Sigma w(F_o^2 - F_c^2)^2/\text{no. data} - \text{no. params})]^{1/2}$ for all data.

Table S2. Coordinates and final energy for a single point energy calculation on geometry optimized

1.

1.C	-0.558119	-1.009241	-7.266183
2.C	-0.446411	1.341059	-6.345715
3.C	-0.412855	-0.156066	-5.990296
4.C	3.464809	0.785844	-5.168050
5.C	-2.818398	-0.277220	-5.262547
6.C	-1.472174	-0.523823	-4.953291
7.C	-3.702388	3.285565	-4.297532
8.C	0.842005	-2.419762	-4.227498
9.C	-3.836973	-0.588895	-4.367709
10.C	2.166440	-2.453094	-3.952550
11.C	-5.235725	5.053574	-3.338077
12.C	3.653382	1.046049	-3.659387
13.C	4.259198	2.441028	-3.442121
14.C	-1.165980	-1.126245	-3.705498
15.C	-6.245820	2.129547	-2.971549
16.C	-4.371020	3.818963	-3.018786
17.C	4.462898	-0.077139	-3.015812

18.C	5.850663	0.056325	-2.870064
19.C	-3.519816	-1.172799	-3.144520
20.C	0.147748	3.420817	-2.931348
21.C	3.856890	-1.303442	-2.633644
22.C	-6.994884	1.120740	-2.375431
23.C	6.636382	-0.986167	-2.379413
24.C	-2.194311	-1.470805	-2.799330
25.C	4.523279	-4.863143	-2.506509
26.C	-5.172509	2.735732	-2.298885
27.C	4.639479	-2.356238	-2.098684
28.C	6.028557	-2.175432	-1.992343
29.C	0.378978	5.735658	-1.936289
30.C	-1.969820	-3.772062	-1.846867
31.C	4.040253	-3.683236	-1.640621
32.C	-0.148313	4.303234	-1.710022
33.C	-6.687016	0.706376	-1.078829
34.C	-1.910967	-2.263111	-1.531484
35.C	-4.875709	2.306163	-0.982350
36.C	-5.640651	1.291863	-0.352613
37.C	0.411878	3.744730	-0.407370
38.C	4.352037	-3.954524	-0.158109
39.C	0.916948	-5.454560	-0.097880
40.C	1.778185	3.391236	-0.324706
41.C	-2.859862	-1.924114	-0.375711
42.C	-3.873597	4.314629	0.046728
43.C	-2.690686	4.759048	0.550437
44.C	5.071925	0.381023	0.768308
45.C	-0.391749	3.638528	0.775870
46.C	-5.369448	0.878799	1.093711
47.C	2.345604	2.976949	0.892606
48.C	-5.983582	-0.476123	1.470864
49.C	0.966755	-5.211016	1.419730
50.C	-1.524943	-5.445694	1.739868
51.C	1.400493	-6.500798	2.146061
52.C	4.642432	-0.428284	1.995349
53.C	-0.361095	-4.691815	1.964743
54.C	-5.853922	1.957605	2.085026
55.C	-2.756785	-5.046939	2.247914
56.C	0.183174	3.275508	2.033479
57.C	5.806093	-1.320893	2.476935
58.C	1.556304	2.937672	2.054530
59.C	-0.463925	-3.504335	2.728610
60.C	-2.841949	-3.880436	3.006095
61.C	4.132457	0.422015	3.151188
62.C	-1.710022	-3.097257	3.271948
63.C	4.794596	1.610013	3.496764
64.C	-0.556320	3.302445	3.372494
65.C	-1.655927	2.241703	3.535758
66.C	-1.087676	4.704236	3.724902
67.C	3.061834	-0.014199	3.966342
68.C	1.608218	-3.306227	4.013135
69.C	-3.167407	-1.167363	4.113743
70.C	-1.820903	-1.896352	4.207245
71.C	2.557752	-2.396693	4.338528
72.C	4.435442	2.335665	4.630733
73.C	2.722667	0.681402	5.155462
74.C	3.420850	1.860794	5.459499

75.C	-1.546915	-2.321477	5.664819
76.C	1.692533	0.144533	6.146750
77.C	0.668727	1.198308	6.597878
78.C	2.398036	-0.463993	7.377353
79.H	0.243150	-0.773875	-7.983112
80.H	-1.522085	-0.817320	-7.762102
81.H	-0.510323	-2.084031	-7.041859
82.H	0.350063	1.579459	-7.067322
83.H	-1.405866	1.626544	-6.802410
84.H	-3.069171	0.177215	-6.222838
85.H	4.438078	0.793219	-5.683556
86.H	2.832424	1.566473	-5.617549
87.H	0.572829	-0.370518	-5.558175
88.H	-0.300617	1.964578	-5.453495
89.H	2.990170	-0.188071	-5.349761
90.H	-4.449938	2.973374	-5.043239
91.H	0.287108	-3.007558	-4.949166
92.H	-3.083738	4.071771	-4.757381
93.H	-6.044514	4.801994	-4.041675
94.H	2.937574	-3.058135	-4.413125
95.H	-4.876501	-0.375068	-4.616499
96.H	5.199326	2.570404	-3.998928
97.H	-4.621708	5.840050	-3.803096
98.H	-6.486555	2.450222	-3.987056
99.H	3.565653	3.211020	-3.809963
100.H	-3.063051	2.418896	-4.082953
101.H	-0.304678	3.863959	-3.829486
102.H	4.300844	-4.708983	-3.571265
103.H	6.332657	0.987776	-3.166111
104.H	-7.819830	0.655060	-2.916375
105.H	2.658352	1.044904	-3.192230
106.H	-5.699437	5.472167	-2.433306
107.H	-0.075607	6.168551	-2.840111
108.H	1.227875	3.330908	-3.117016
109.H	4.467132	2.639570	-2.381209
110.H	7.716912	-0.866019	-2.294002
111.H	-0.260908	2.408054	-2.810455
112.H	-3.570163	4.145998	-2.342931
113.H	-1.227570	-4.054760	-2.606937
114.H	5.610604	-5.007178	-2.410160
115.H	4.032833	-5.796097	-2.190276
116.H	-4.321266	-1.415467	-2.447632
117.H	1.471734	5.736253	-2.073520
118.H	-2.968489	-4.039229	-2.227555
119.H	6.641656	-2.986948	-1.596257
120.H	2.949939	-3.611669	-1.747212
121.H	-1.239083	4.365573	-1.597811
122.H	0.145337	6.391905	-1.086100
123.H	-7.283042	-0.081696	-0.620374
124.H	-0.885459	-2.032089	-1.207335
125.H	-1.780341	-4.358607	-0.936738
126.H	2.402331	3.449360	-1.216038
127.H	0.159247	-6.206967	-0.362713
128.H	1.887016	-5.828979	-0.455361
129.H	0.681555	-4.533919	-0.646957
130.H	-3.878499	-2.288679	-0.575365
131.H	-4.793772	4.870094	-0.094778

132.H	5.426384	-0.294276	-0.021063
133.H	5.435449	-4.029585	0.018173
134.H	-2.904788	-0.842180	-0.202653
135.H	3.899831	-4.904001	0.161932
136.H	4.234450	0.957144	0.354407
137.H	3.958440	-3.157018	0.486058
138.H	-2.431815	5.755745	0.888482
139.H	-2.517006	-2.415900	0.544886
140.H	5.893469	1.077115	0.995759
141.H	-5.666837	-1.276856	0.789489
142.H	-1.459535	-6.365626	1.156395
143.H	3.400582	2.722467	0.953292
144.H	-4.277708	0.790032	1.207440
145.H	-7.083272	-0.435563	1.471167
146.H	0.686040	-7.317202	1.957820
147.H	6.151106	-1.966891	1.656621
148.H	2.388247	-6.829766	1.788753
149.H	1.727638	-4.443107	1.612805
150.H	3.822035	-1.092487	1.690597
151.H	-5.380318	2.928995	1.892462
152.H	-3.649238	-5.644443	2.058396
153.H	-6.945394	2.084635	2.011405
154.H	-5.671638	-0.753697	2.486676
155.H	6.656173	-0.704179	2.809091
156.H	5.617826	1.963941	2.875521
157.H	1.462642	-6.358735	3.233859
158.H	-5.614772	1.660068	3.117575
159.H	-2.503886	2.433820	2.866304
160.H	5.502225	-1.962644	3.315429
161.H	-1.943978	4.975067	3.092856
162.H	2.014049	2.640712	2.998919
163.H	-3.400081	-0.882724	3.079562
164.H	-3.808067	-3.580195	3.411315
165.H	-1.270927	1.232376	3.332034
166.H	-0.308263	5.471124	3.609571
167.H	-1.040244	-1.178616	3.918635
168.H	0.223550	3.066445	4.112955
169.H	-3.994246	-1.784290	4.496025
170.H	1.485402	-4.316476	4.384586
171.H	-2.031298	2.256815	4.570222
172.H	4.959918	3.258722	4.879629
173.H	-1.427227	4.718130	4.771897
174.H	-3.140802	-0.252794	4.722130
175.H	3.389919	-2.504501	5.024030
176.H	-0.555116	-2.782599	5.769527
177.H	1.141750	-0.664318	5.647934
178.H	0.115384	1.611529	5.744863
179.H	-2.301226	-3.050176	6.001443
180.H	3.169870	2.412169	6.366986
181.H	-1.593553	-1.448680	6.334079
182.H	1.145894	2.032713	7.133214
183.H	3.110777	-1.248232	7.088628
184.H	-0.059272	0.743183	7.285480
185.H	2.954172	0.307644	7.932344
186.H	1.659866	-0.910114	8.060804
187.B	1.208263	-0.894948	-2.510334
188.B	-2.459414	2.478160	0.091791

189.B	1.171343	-1.396459	2.748688
190.N	0.200842	-1.475982	-3.409168
191.N	2.469015	-1.532743	-2.927623
192.N	-3.803203	2.936280	-0.261668
193.N	-1.811376	3.671340	0.627694
194.N	0.722387	-2.762055	3.063493
195.N	2.343934	-1.208454	3.618322
196.O	1.012333	-0.006546	-1.481777
197.O	-1.776808	1.289169	-0.006376
198.O	0.653627	-0.505220	1.843423
199.U	0.366284	1.004871	0.313846

Energy: −1190.07111658 eV

Table S3. Coordinates and final energy for a single point energy calculation on geometry optimized 2.

1.C	-0.752371	-3.356672	-5.118734
2.C	-1.441759	-0.940093	-5.068688
3.C	-1.309305	-2.222253	-4.234385
4.C	-3.848550	-2.241069	-4.047357
5.C	2.898921	-4.054005	-3.630559
6.C	-5.041321	-2.639435	-3.445229
7.C	-2.603542	-2.642275	-3.539298
8.C	3.920135	-1.917930	-2.775455
9.C	-1.552386	6.822030	-2.467694
10.C	2.907310	-3.031856	-2.475501
11.C	-5.009701	-3.445819	-2.309541
12.C	-3.920438	1.168648	-2.439323
13.C	-2.590519	-3.468412	-2.387742
14.C	-0.366687	4.597079	-2.390660
15.C	-0.964164	-5.289231	-2.134967
16.C	-2.512697	1.492915	-1.951449
17.C	-3.792544	-3.875824	-1.757477
18.C	0.268785	-5.522568	-1.621278
19.C	-1.127996	5.653473	-1.564942
20.C	-3.595783	5.074535	-1.297800
21.C	-4.573182	0.512062	-1.210763
22.C	3.132211	-3.724292	-1.133036
23.C	-2.287050	5.023718	-0.792923
24.C	3.697350	3.826917	-0.768881
25.C	-4.654260	4.471745	-0.619542
26.C	4.419383	-3.800861	-0.578737
27.C	-4.971086	-5.684416	-0.377206
28.C	-3.778298	-4.720950	-0.484666
29.C	-3.432168	-0.306020	-0.621862
30.C	2.069906	-4.347385	-0.433911
31.C	4.971495	5.684147	0.377164
32.C	3.778494	4.720953	0.484732
33.C	-2.069810	4.347479	0.434330
34.C	-4.419326	3.801027	0.579238
35.C	4.654347	-4.471744	0.619939
36.C	-3.697436	-3.826821	0.768886
37.C	2.287095	-5.023675	0.793321
38.C	3.431779	0.306348	0.621353

39.C	-3.132139	3.724498	1.133539
40.C	3.595860	-5.074601	1.298144
41.C	4.572954	-0.511419	1.210358
42.C	1.128051	-5.653465	1.565305
43.C	-0.268497	5.522511	1.621658
44.C	3.792757	3.875720	1.757494
45.C	2.512682	-1.492891	1.950790
46.C	0.964522	5.289046	2.135100
47.C	5.009937	3.445824	2.309578
48.C	1.552438	-6.822401	2.467575
49.C	0.367018	-4.597190	2.391442
50.C	2.590745	3.468129	2.387652
51.C	3.920323	-1.168352	2.438788
52.C	-2.907253	3.032451	2.476192
53.C	-3.920151	1.918795	2.776853
54.C	5.041595	2.639309	3.445173
55.C	-2.898622	4.055090	3.630831
56.C	2.603807	2.641796	3.539076
57.C	3.848837	2.240644	4.047126
58.C	1.309615	2.221644	4.234137
59.C	0.752782	3.355749	5.118943
60.C	1.442030	0.939162	5.067964
61.H	-2.057028	-1.097914	-5.967976
62.H	-1.463886	-3.603758	-5.922420
63.H	0.192263	-3.039939	-5.585771
64.H	-0.446937	-0.616185	-5.403362
65.H	-3.884955	-1.613863	-4.937784
66.H	2.705070	-3.543655	-4.585938
67.H	-1.880824	-0.115434	-4.491639
68.H	-0.554989	-4.266802	-4.537473
69.H	-5.996992	-2.321893	-3.864678
70.H	3.874747	-4.559282	-3.705294
71.H	3.604088	-1.365839	-3.670773
72.H	2.123080	-4.818163	-3.490067
73.H	-2.163832	6.480816	-3.317186
74.H	-0.569662	-2.016964	-3.446632
75.H	-3.891186	0.458076	-3.277619
76.H	-0.660833	7.307611	-2.890054
77.H	4.926468	-2.318112	-2.971857
78.H	-1.010606	4.184758	-3.183304
79.H	0.511223	5.051444	-2.874024
80.H	-4.443852	2.078480	-2.762319
81.H	-1.609450	-5.965079	-2.684589
82.H	-1.732665	1.415427	-2.718851
83.H	1.915102	-2.560245	-2.437936
84.H	-3.791162	5.594995	-2.235301
85.H	-2.126892	7.581048	-1.918107
86.H	-5.947789	-3.749823	-1.844550
87.H	3.988439	-1.195270	-1.952046
88.H	0.857975	-6.431837	-1.657823
89.H	-0.015688	3.762380	-1.770328
90.H	3.646533	4.445547	-1.677450
91.H	-5.423119	-0.130506	-1.476897
92.H	-5.069813	-6.316072	-1.271572
93.H	-2.458515	2.472146	-1.460501
94.H	-5.665526	4.527060	-1.024985
95.H	5.254547	-3.337425	-1.104171

96.H	-3.332534	-1.285238	-1.106962
97.H	-0.421014	6.051405	-0.821857
98.H	4.589370	3.185175	-0.847186
99.H	4.841044	6.342935	-0.493798
100.H	2.810614	3.179729	-0.751883
101.H	-4.910726	1.279998	-0.500282
102.H	-2.864024	-5.330515	-0.505975
103.H	-5.921668	-5.148301	-0.233867
104.H	3.467225	0.431226	-0.467747
105.H	5.921882	5.147787	0.233446
106.H	-4.840675	-6.342982	0.493934
107.H	-3.467611	-0.430896	0.467236
108.H	2.864345	5.330708	0.506180
109.H	4.910844	-1.279191	0.499861
110.H	-4.589573	-3.185224	0.847058
111.H	-2.810809	-3.179483	0.751925
112.H	0.420889	-6.051064	0.822209
113.H	5.665621	-4.527134	1.025351
114.H	5.070653	6.315571	1.271643
115.H	-5.254486	3.337658	1.104735
116.H	3.331884	1.285546	1.106433
117.H	-3.646598	-4.445372	1.677510
118.H	-0.857594	6.431838	1.658336
119.H	5.422655	0.131401	1.476617
120.H	2.458725	-2.472094	1.459785
121.H	2.126795	-7.581280	1.917641
122.H	5.948015	3.749993	1.844677
123.H	0.015858	-3.762405	1.771307
124.H	3.791246	-5.595148	2.235596
125.H	-3.988428	1.195559	1.953940
126.H	1.609919	5.964790	2.684722
127.H	-1.915101	2.560716	2.438713
128.H	0.660892	-7.308033	2.889895
129.H	4.443948	-2.078116	2.761630
130.H	-0.510779	-5.051600	2.874966
131.H	1.732605	-1.415655	2.718177
132.H	-4.926485	2.319130	2.972948
133.H	2.164031	-6.481542	3.317111
134.H	1.011174	-4.184988	3.183956
135.H	3.890877	-0.457907	3.277172
136.H	-2.122738	4.819133	3.489947
137.H	-3.874407	4.560471	3.705456
138.H	0.569888	2.016690	3.446384
139.H	5.997281	2.321835	3.864629
140.H	-3.604140	1.367310	3.672561
141.H	0.555561	4.266169	4.538079
142.H	-2.704710	3.545136	4.586412
143.H	1.881001	0.114677	4.490601
144.H	3.885260	1.613281	4.937444
145.H	-0.191914	3.038921	5.585786
146.H	0.447198	0.615202	5.402559
147.H	1.464284	3.602375	5.922787
148.H	2.057356	1.096594	5.967279
149.B	-0.255808	-3.305677	-1.145012
150.B	0.255869	3.305610	1.145119
151.Cl	1.090671	0.661193	-2.320333
152.Cl	-1.091097	-0.661138	2.320034

153.N	-1.338600	-3.955828	-1.885701
154.N	0.757739	-4.353181	-1.011615
155.N	-0.757616	4.353206	1.011962
156.N	1.338822	3.955627	1.885704
157.O	-2.209144	0.470046	-0.923404
158.O	-0.205936	-2.021550	-0.672124
159.O	0.205809	2.021536	0.672104
160.O	2.208922	-0.469991	0.922853
161.U	-0.000162	0.000025	-0.000122

Energy: –947.02515744 eV

Table S4. Coordinates and final energy for a single point energy calculation on geometry optimized 3.

1.C	1.244396	1.280491	-6.078793
2.C	-2.181435	-1.340365	-5.174662
3.C	-2.528854	2.842389	-5.339845
4.C	-3.303496	5.162297	-4.715297
5.C	1.598026	-2.639427	-4.715260
6.C	1.249762	0.937145	-4.581882
7.C	0.470947	-3.368825	-4.509782
8.C	3.700832	1.378266	-4.140838
9.C	-2.775370	3.821312	-4.180659
10.C	2.621455	0.482139	-4.113088
11.C	0.757084	2.121401	-3.737232
12.C	4.734008	-3.592436	-3.546114
13.C	4.953286	1.009528	-3.658634
14.C	-2.503238	-1.358639	-3.667864
15.C	2.843659	-0.812628	-3.604279
16.C	-4.966856	2.743872	-3.529388
17.C	-3.778077	-0.557183	-3.412968
18.C	-0.010606	-6.810430	-3.135581
19.C	-2.566048	-2.777843	-3.119307
20.C	5.143822	-0.262602	-3.117920
21.C	4.096658	-1.191752	-3.071548
22.C	-3.705984	3.218336	-3.138714
23.C	-3.795101	-3.380909	-2.824777
24.C	-1.393323	-3.538419	-2.915965
25.C	-3.866523	-4.698395	-2.372096
26.C	-5.813860	2.129712	-2.613651
27.C	4.293221	-2.577322	-2.476091
28.C	-1.442894	-4.853546	-2.415430
29.C	-2.698811	-5.426071	-2.167301
30.C	3.461465	4.668688	-2.389738
31.C	-0.177416	-5.650419	-2.140167
32.C	2.303998	6.841541	-1.839924
33.C	2.276716	5.314061	-1.656025
34.C	-3.333783	3.102329	-1.783617
35.C	5.270833	-2.588559	-1.295742
36.C	-2.020404	5.119689	-1.313214

37.C	-5.409007	1.979054	-1.287551
38.C	-0.855301	5.489664	-0.726205
39.C	-0.146843	-6.168590	-0.694200
40.C	-4.179576	2.476266	-0.837662
41.C	2.805270	1.395547	-0.641050
42.C	2.234858	4.940240	-0.181181
43.C	4.249821	1.494442	-0.162325
44.C	1.058569	4.432469	0.415698
45.C	3.369077	5.117805	0.623837
46.C	2.302552	-3.605714	0.433114
47.C	-3.804228	2.395208	0.636857
48.C	-3.117980	-4.186034	1.040824
49.C	-3.886426	3.774478	1.317371
50.C	4.133636	1.378107	1.364526
51.C	-4.642633	1.391351	1.427603
52.C	3.004356	0.381180	1.534298
53.C	1.032350	4.064833	1.779170
54.C	3.348314	4.790932	1.978671
55.C	1.996855	-3.662378	1.933839
56.C	-2.368992	-3.573608	2.223578
57.C	-0.224484	3.531153	2.444938
58.C	2.191406	4.256086	2.543058
59.C	4.335225	-2.839700	2.467082
60.C	1.966260	-5.105436	2.461999
61.C	2.961271	-2.810283	2.742519
62.C	-4.558195	-2.397022	2.777731
63.C	-3.174594	-2.510402	2.962761
64.C	-0.846835	4.590761	3.368038
65.C	-1.892061	-4.700390	3.159492
66.C	5.232094	-2.043868	3.179245
67.C	0.041102	2.221558	3.195619
68.C	-5.309781	-1.456116	3.480961
69.C	2.507644	-1.970307	3.781345
70.C	-2.558758	-1.618154	3.868825
71.C	4.759177	-1.185829	4.171607
72.C	-4.681525	-0.578801	4.359109
73.C	3.394156	-1.124813	4.483362
74.C	-3.293757	-0.629165	4.553527
75.C	-3.085174	1.807728	5.220643
76.C	0.633207	-2.458804	5.322737
77.C	-0.718399	-2.337881	5.342082
78.C	3.364357	1.293594	5.258214
79.C	-2.611692	0.369372	5.477681
80.C	2.886093	-0.142248	5.529584
81.C	-2.807382	0.006025	6.959856
82.C	3.277885	-0.582965	6.950102
83.H	1.570158	0.422618	-6.683333
84.H	0.232069	1.564056	-6.400234
85.H	1.917190	2.124687	-6.295261
86.H	-1.786353	3.253469	-6.039387
87.H	-3.451325	2.648223	-5.905944
88.H	-2.967574	-1.869500	-5.735971

89.H	-2.599293	5.593260	-5.442750
90.H	-2.151767	-0.301687	-5.536647
91.H	2.274938	-2.650396	-5.560768
92.H	-1.216492	-1.809562	-5.403547
93.H	-4.272793	5.028483	-5.219062
94.H	0.018015	-4.118881	-5.146690
95.H	-2.157603	1.876922	-4.971675
96.H	3.550166	2.382272	-4.537629
97.H	3.999180	-3.655540	-4.358037
98.H	0.553243	0.101628	-4.430316
99.H	-5.274968	2.836476	-4.571850
100.H	-0.243805	2.432880	-4.058862
101.H	-3.445535	5.889923	-3.905211
102.H	5.705991	-3.306515	-3.977328
103.H	0.012393	-6.445500	-4.171526
104.H	1.426505	2.988624	-3.836027
105.H	-4.619749	-0.937242	-4.012938
106.H	5.780213	1.719467	-3.685453
107.H	-1.812150	4.011089	-3.687924
108.H	-3.620204	0.488191	-3.702106
109.H	4.833746	-4.594899	-3.102853
110.H	3.430521	4.930872	-3.457548
111.H	-1.683106	-0.845723	-3.140678
112.H	-4.712721	-2.809986	-2.954425
113.H	0.925276	-7.356575	-2.942675
114.H	2.270218	7.102142	-2.908163
115.H	-0.845438	-7.522759	-3.047349
116.H	-6.784006	1.749538	-2.933583
117.H	0.697056	1.860043	-2.674040
118.H	6.121109	-0.532158	-2.717811
119.H	-4.836231	-5.149711	-2.160738
120.H	0.673011	-4.967165	-2.274492
121.H	-4.061680	-0.567188	-2.354291
122.H	-2.758831	-6.448138	-1.790413
123.H	3.310234	-2.900588	-2.105750
124.H	3.439591	3.574992	-2.310495
125.H	1.351992	4.933620	-2.110867
126.H	4.425047	5.020940	-1.992933
127.H	6.294837	-2.339916	-1.611137
128.H	-2.820931	5.744787	-1.688447
129.H	3.224702	7.268437	-1.413212
130.H	1.451136	7.322733	-1.343903
131.H	2.690338	0.840216	-1.577614
132.H	5.307001	-3.594105	-0.853313
133.H	-0.484759	6.484120	-0.512742
134.H	4.969854	-1.882924	-0.509542
135.H	-6.068898	1.477255	-0.582342
136.H	2.311507	2.372375	-0.710079
137.H	0.810482	-6.667436	-0.482776
138.H	-0.951667	-6.895485	-0.508882
139.H	4.839817	0.664644	-0.575038
140.H	4.707635	2.439567	-0.482784

141.H	-0.272645	-5.345554	0.022359
142.H	4.281687	5.520757	0.182260
143.H	1.501288	-4.084615	-0.141915
144.H	2.388270	-2.568652	0.081371
145.H	3.239538	-4.125264	0.187726
146.H	-3.471766	-3.422448	0.338535
147.H	-2.447165	-4.852928	0.487092
148.H	-3.224378	4.515171	0.851144
149.H	-2.759761	2.051586	0.685001
150.H	-4.918245	4.155784	1.271985
151.H	-4.645549	0.400061	0.960889
152.H	-5.680883	1.738028	1.545332
153.H	-3.974661	-4.790488	1.376867
154.H	3.348249	-0.656854	1.453652
155.H	5.055216	1.014110	1.837539
156.H	-0.957059	3.319502	1.657101
157.H	3.861304	2.345867	1.804614
158.H	4.706745	-3.486685	1.673047
159.H	-1.477352	-3.070348	1.817521
160.H	1.242421	-5.706827	1.894790
161.H	-3.607607	3.683965	2.377429
162.H	0.992520	-3.247749	2.073391
163.H	-5.056479	-3.056263	2.069663
164.H	4.236665	4.941879	2.592723
165.H	-4.222354	1.270489	2.433189
166.H	2.416788	0.497472	2.448674
167.H	-1.110893	5.494561	2.802081
168.H	2.956848	-5.577366	2.369794
169.H	-1.314802	-5.439596	2.584184
170.H	0.571122	1.498040	2.562264
171.H	6.296935	-2.080398	2.948931
172.H	-6.387250	-1.395371	3.326181
173.H	2.182003	3.992278	3.601396
174.H	1.672476	-5.123774	3.520556
175.H	-2.758153	-5.217709	3.600931
176.H	-1.760670	4.201377	3.838100
177.H	-0.898543	1.759607	3.522121
178.H	-0.145258	4.876613	4.167051
179.H	-1.257116	-4.332155	3.974350
180.H	0.661128	2.379549	4.090201
181.H	-3.007342	2.072962	4.158135
182.H	3.081750	1.630132	4.252138
183.H	5.460010	-0.541568	4.704925
184.H	-5.272338	0.171996	4.885184
185.H	-1.534632	0.321854	5.261531
186.H	-4.133816	1.947707	5.520291
187.H	4.457236	1.380942	5.347192
188.H	1.789185	-0.144763	5.468279
189.H	-2.478005	2.519119	5.798442
190.H	1.290736	-2.891552	6.066047
191.H	-1.417942	-2.665877	6.100337
192.H	2.916554	1.983838	5.988314

193.H	4.372040	-0.585521	7.071142
194.H	-3.876223	0.015881	7.222741
195.H	2.914795	-1.596193	7.167203
196.H	-2.413193	-0.993092	7.183082
197.H	-2.288296	0.729190	7.606927
198.H	2.852250	0.100697	7.699597
199.B	0.684758	-1.943697	-2.699329
200.B	-0.920819	3.169869	-0.727285
201.B	-0.004146	-1.498958	3.308144
202.Cl	-2.618269	-0.926525	-0.119407
203.N	1.782882	-1.763823	-3.633084
204.N	-0.129017	-2.985123	-3.297958
205.N	-2.118931	3.717465	-1.342130
206.N	-0.136958	4.339894	-0.353249
207.N	1.123374	-1.958780	4.105569
208.N	-1.161296	-1.756923	4.142214
209.O	0.514572	-1.262649	-1.530500
210.O	-0.572263	1.869525	-0.528363
211.O	2.093530	0.640235	0.408579
212.O	0.057981	-0.949449	2.063412
213.U	-0.196660	-0.130156	0.108056

Energy: −1282.05374758 eV

Table S5. Coordinates and final energy for a single point energy calculation on geometry optimized 4.

1.C	1.390876	-0.318355	-5.209127
2.C	-3.056294	-1.000397	-5.218084
3.C	-1.927270	-0.231968	-4.937416
4.C	-3.158499	-2.296186	-4.719173
5.C	-2.303596	3.916611	-4.669262
6.C	4.298924	2.210814	-4.551501
7.C	-2.066005	-5.350310	-4.429032
8.C	-0.854947	-0.764123	-4.210010
9.C	0.438059	0.019848	-4.047443
10.C	4.749814	4.644659	-4.045027
11.C	-1.168101	6.135334	-4.259887
12.C	0.224171	1.530402	-3.949121
13.C	-2.126703	-2.858010	-3.953960
14.C	0.928239	-3.627207	-3.911205
15.C	-0.960556	-2.091551	-3.743532
16.C	-1.419929	4.722578	-3.705106
17.C	4.077099	3.353204	-3.551273
18.C	-2.276811	-4.254903	-3.369094
19.C	2.001767	-4.044252	-3.196849
20.C	-5.410371	0.419452	-2.934399
21.C	-3.638687	-4.441827	-2.681781
22.C	5.595807	-1.195463	-2.480848
23.C	-3.360117	5.077983	-2.111793
24.C	-2.007564	4.765942	-2.303581
25.C	-4.924544	1.719433	-2.266554

26.C	4.540071	2.970455	-2.153894
27.C	-5.299669	-0.743832	-1.929312
28.C	5.822737	2.446129	-1.949977
29.C	4.197846	-1.580153	-1.982595
30.C	-3.458011	1.542873	-1.833794
31.C	-3.839601	-0.909221	-1.466683
32.C	1.003681	5.450836	-1.505453
33.C	2.309768	5.092782	-1.446151
34.C	2.191639	-6.976515	-1.196032
35.C	3.517778	-0.393882	-1.286278
36.C	-5.802127	2.022906	-1.035840
37.C	4.196679	-2.787480	-1.061316
38.C	-3.923914	5.087299	-0.837966
39.C	3.090641	-3.662864	-1.043307
40.C	-1.216531	4.494836	-1.167065
41.C	-6.182312	-0.440066	-0.704492
42.C	3.687982	3.108982	-1.037683
43.C	6.245579	2.052542	-0.681136
44.C	-3.338936	0.393436	-0.812276
45.C	1.904272	-5.782473	-0.268433
46.C	5.270486	-3.058520	-0.202863
47.C	3.075740	-4.815316	-0.233305
48.C	-5.695726	0.859030	-0.031631
49.C	-3.139277	4.772027	0.269671
50.C	-1.774702	4.489774	0.127878
51.C	5.385010	2.179718	0.407623
52.C	4.100415	2.718225	0.252384
53.C	4.180952	-5.060129	0.593260
54.C	5.264445	-4.185323	0.618651
55.C	-4.226819	0.710474	0.411529
56.C	-1.695813	-3.891966	0.246377
57.C	1.510637	-6.274965	1.128463
58.C	-0.926839	4.172525	1.345655
59.C	3.167711	2.843306	1.441936
60.C	-4.244362	-3.128293	1.592104
61.C	-1.749512	-3.584227	1.742847
62.C	2.598189	1.466683	1.812950
63.C	-5.410836	-2.544672	2.078225
64.C	2.355615	-2.294218	2.142449
65.C	-1.283939	2.784017	1.886288
66.C	-2.998711	-2.829155	2.161701
67.C	-1.048373	5.239806	2.442510
68.C	-1.650667	-4.871068	2.582950
69.C	3.841493	3.514617	2.646168
70.C	-5.349013	-1.613402	3.115047
71.C	-2.957707	-1.909649	3.225520
72.C	-4.124097	-1.266503	3.697862
73.C	2.583891	-2.502426	3.649706
74.C	3.721686	-3.496258	3.903899
75.C	-3.468561	1.104041	4.163797
76.C	2.785463	-1.154763	4.320182
77.C	4.060374	-0.611626	4.528792
78.C	-4.022174	-0.194862	4.774771
79.C	1.665496	-0.383740	4.693399
80.C	4.213870	0.656602	5.089850
81.C	-1.451091	-2.135448	5.171728
82.C	-5.336353	0.071323	5.514809

83.C	0.726699	3.218382	5.250334
84.C	1.799306	0.911990	5.228667
85.C	3.093211	1.412684	5.429529
86.C	-0.220682	-1.714093	5.568246
87.C	0.567738	1.731164	5.583616
88.C	0.180946	1.542240	7.060654
89.H	0.934644	-0.023416	-6.167640
90.H	1.610591	-1.393602	-5.246769
91.H	-3.864734	-0.581203	-5.816286
92.H	2.339488	0.224245	-5.101044
93.H	-1.796360	3.798219	-5.637943
94.H	-1.865870	0.788243	-5.314096
95.H	3.834031	2.451486	-5.518248
96.H	-2.812182	-5.261195	-5.233579
97.H	4.365398	4.923467	-5.037574
98.H	-0.722959	6.080041	-5.264747
99.H	-4.062061	-2.875822	-4.910437
100.H	-3.260280	4.424910	-4.859893
101.H	-1.068044	-5.287241	-4.880076
102.H	0.641697	-3.880517	-4.924276
103.H	5.369131	2.030860	-4.730963
104.H	-0.122012	1.952372	-4.903259
105.H	5.839636	4.511601	-4.125142
106.H	-2.110601	6.699588	-4.332655
107.H	-2.524729	2.920524	-4.266130
108.H	3.858796	1.275591	-4.182655
109.H	-2.169752	-6.347384	-3.975062
110.H	1.171678	2.024097	-3.694878
111.H	-4.797978	0.206078	-3.822749
112.H	4.561898	5.481812	-3.360189
113.H	-0.482090	6.701392	-3.616819
114.H	0.921530	-0.309316	-3.116987
115.H	2.798544	-4.717685	-3.489255
116.H	-4.461605	-4.435931	-3.411613
117.H	2.996235	3.541823	-3.490812
118.H	-0.449324	4.214643	-3.627333
119.H	-6.457495	0.528916	-3.268054
120.H	-3.986624	5.300994	-2.975891
121.H	-0.505021	1.784588	-3.169746
122.H	5.519046	-0.383177	-3.216361
123.H	6.495849	2.330797	-2.800642
124.H	6.102687	-2.049235	-2.953061
125.H	-4.986826	2.556836	-2.981830
126.H	-5.630340	-1.680568	-2.409375
127.H	3.589584	-1.846958	-2.860006
128.H	-1.492661	-4.367931	-2.607778
129.H	-2.824570	1.300461	-2.701919
130.H	-3.668302	-5.410934	-2.162597
131.H	-3.181571	-1.151705	-2.311559
132.H	2.410316	-6.643409	-2.218442
133.H	-3.828336	-3.647380	-1.950153
134.H	6.229587	-0.823376	-1.663056
135.H	3.384331	0.453948	-1.970278
136.H	3.192646	5.715387	-1.522938
137.H	0.571843	6.436152	-1.630984
138.H	-6.852318	2.162707	-1.347695
139.H	-7.237356	-0.331366	-1.012165

140.H	1.321790	-7.649370	-1.236143
141.H	-3.067879	2.462268	-1.377224
142.H	-4.978745	5.327547	-0.710244
143.H	-3.760315	-1.721941	-0.728094
144.H	7.245171	1.639780	-0.543320
145.H	3.055764	-7.551898	-0.829328
146.H	1.051797	-5.233006	-0.692772
147.H	2.533554	-0.696182	-0.909956
148.H	-5.469149	2.958599	-0.560653
149.H	-6.128153	-1.272951	0.010980
150.H	6.130232	-2.388706	-0.190308
151.H	4.118565	-0.051680	-0.433154
152.H	-2.469864	-4.614045	-0.048422
153.H	-1.820917	-2.983957	-0.357427
154.H	-0.721932	-4.330372	-0.008676
155.H	-6.317527	1.069750	0.855202
156.H	4.190143	-5.944725	1.230172
157.H	6.111256	-4.387354	1.275023
158.H	-3.874662	1.646030	0.874367
159.H	0.584944	-6.864637	1.074693
160.H	-3.589963	4.759918	1.263097
161.H	-4.112902	-0.094877	1.151880
162.H	5.714517	1.859580	1.397081
163.H	-4.302452	-3.845811	0.773928
164.H	0.120793	4.154092	1.018165
165.H	2.283230	0.945070	0.894416
166.H	2.331866	3.483792	1.129887
167.H	2.285812	-6.921518	1.564891
168.H	1.345411	-5.435165	1.816098
169.H	-1.449510	2.073291	1.053291
170.H	-6.374279	-2.811232	1.645365
171.H	2.083188	-3.234146	1.647997
172.H	-0.790277	6.234228	2.053691
173.H	3.262778	-1.908817	1.654928
174.H	1.544085	-1.576336	1.976617
175.H	-2.523953	-5.515001	2.392454
176.H	3.355979	0.826582	2.284999
177.H	4.241383	4.500235	2.370000
178.H	-0.744875	-5.435073	2.328705
179.H	-2.244573	2.801361	2.417802
180.H	-0.878679	-2.961215	1.986289
181.H	-2.071482	5.285872	2.845148
182.H	4.666384	2.903119	3.038517
183.H	1.753338	1.547349	2.508830
184.H	-0.525272	2.398441	2.578226
185.H	-0.373395	5.009147	3.277736
186.H	-6.267128	-1.146329	3.468797
187.H	-4.178431	1.516337	3.430835
188.H	3.120884	3.645634	3.464419
189.H	3.463464	-4.474410	3.475658
190.H	-1.624105	-4.639122	3.656460
191.H	4.656309	-3.176517	3.420663
192.H	-2.522391	0.917421	3.642406
193.H	1.660847	-2.934082	4.065413
194.H	4.942463	-1.188234	4.250479
195.H	1.023045	3.364024	4.203122
196.H	-3.292252	1.858849	4.944860

197.H	-6.090508	0.522514	4.852762
198.H	3.910554	-3.624280	4.979576
199.H	5.212587	1.061621	5.254843
200.H	-3.285006	-0.539192	5.515948
201.H	-0.260998	1.338210	4.977122
202.H	-0.224775	3.743883	5.411591
203.H	-2.163877	-2.756339	5.701572
204.H	1.481628	3.703987	5.885555
205.H	-5.760320	-0.850534	5.936884
206.H	-5.163529	0.777015	6.339699
207.H	3.227486	2.409346	5.849760
208.H	0.308005	-1.919301	6.491844
209.H	-0.726830	2.117164	7.298550
210.H	-0.016451	0.485444	7.282831
211.H	0.991907	1.888997	7.719979
212.B	0.851196	-2.522407	-1.869510
213.B	1.059344	3.165456	-1.084504
214.B	-0.561084	-0.897831	3.425977
215.N	0.183221	-2.710918	-3.151055
216.N	2.000862	-3.419046	-1.938463
217.N	0.193731	4.318285	-1.315133
218.N	2.406633	3.712024	-1.209958
219.N	-1.965074	0.247494	-0.409594
220.N	-1.712774	-1.660253	3.877411
221.N	0.360288	-0.944992	4.546824
222.O	0.493550	-1.674991	-0.857124
223.O	0.701130	1.882713	-0.795126
224.O	-0.379293	-0.293514	2.219977
225.U	-0.119659	0.122773	0.129113

Energy: –1359.95484613 eV

Table S6. Computed bond lengths, bond orders, charges, and spin densities for 1-4.

Cmpd	Bond	Expt (av.) Dist.	Calc. (av.) Dist.	BI (av.) NM[a]	MDC$_q$ charges and MDC$_m$ spin densities: U	O_{NBO}	O_{THF}	Cl	N	Arene
**1**	U-O_{NBO}	2.191	2.171	1.46	**Charges**					
	U-C	2.970	2.838	0.31	2.52	–1.17	–	–	–	–0.94
	B-O	1.348	1.373	1.33	**Spin densities**					
	B-N	1.453	1.468	1.13	–2.51	0.06	–	–	–	0.73
**2**	U-O_{NBO}	2.127	2.140	1.52	**Charges**					
	U-O_{THF}	2.424	2.440	0.63	2.24	–1.07	–0.82	–0.45	–	–
	U-Cl	2.648	2.648	1.45	**Spin densities**					
	B-O	1.372	1.369	1.30	–2.24	0.07	0.01	0.02	–	–
	B-N	1.440	1.464	1.14						
**3**	U-O_{NBO}	2.130	2.127	1.49	**Charges**					
	U-O_{THF}	2.442	2.435	0.62	2.40	–1.18	–0.82	–0.37	–	–
	U-Cl	2.568	2.559	1.55	**Spin densities**					
	B-O	1.361	1.362	1.31	–2.25	0.05	0.02	0.05	–	–
	B-N	1.447	1.453	1.14						
**4**	U-O_{NBO}	2.140	2.152	1.38	**Charges**					
	UN	1.911	1.916	2.80	3.01	–1.25	–	–	–1.17	–
	B-O	1.360	1.364	1.32	**Spin densities**					
	B-N	1.450	1.457	1.14	–1.31	0.04	–	–	0.14	–

[a] Nalewajski-Mrozek bond order.

Table S7. NLMO data for 1-4.

Cmpd	Bond	U[a]	X[b]	U s/p/d/f	X s/p	Bond	U	X[a]	U s/p/d/f	X s/p
**1**	NBO σ	4	95	0/2/36/62	50/50	NBO π	5	92	0/0/24/76	0/100
						NBO π	5	89	0/0/21/79	0/100
	NBO σ	3	96	0/0/29/71	49/51	NBO π	5	91	0/2/44/54	03//97
						NBO π	4	88	0/0/25/75	0/100
	NBO σ	4	95	0/2/34/64	51/49	NBO π	5	92	0/0/27/73	0/100
						NBO π	4	89	0/0/22/78	0/100
**2**	NBO σ	8	92	6/0/26/68	49/51	NBO π	6	91	0/0/34/66	0/100
						NBO π	6	88	0/0/29/71	0/100
	NBO σ	8	92	6/0/26/68	49/51	NBO π	6	91	0/0/34/66	0/100
						NBO π	6	88	0/0/29/71	0/100
	THF σ	5	94	14/0/31/55	47/53	THF π	2	95	7/0/26/67	0/100
	THF σ	5	94	14/0/31/55	47/53	THF π	2	96	0/0/25/75	0/100
	Cl σ	10	90	19/0/34/47	38/62	Cl π	6	94	0/0/46/54	0/100
						Cl π	7	93	0/0/50/50	0/100
	Cl σ	10	90	19/0/34/47	38/62	Cl π	6	94	0/0/46/54	0/100
						Cl π	7	93	0/0/50/50	0/100
**3**	NBO σ	5	95	0/2/36/62	49/51	NBO π	5	92	0/0/23/77	0/100
						NBO π	5	89	0/0/16/84	0/100
	NBO σ	5	95	0/2/34/64	49/51	NBO π	5	92	0/0/18/82	0/100
						NBO π	5	89	0/0/17/83	0/100
	NBO σ	5	95	0/1/32/67	49/51	NBO π	5	92	0/0/21/79	0/100
						NBO π	5	89	0.0/12/88	0/100
	THF σ	3	96	0/0/16/84	26/74	THF π	3	96	0/0/15/85	19/81
	Cl σ	7	93	1/0/29/70	18/82	Cl π	6	94	0/0/24/76	0/100
						Cl π	7	93	1/0/29/70	0/100
**4**	NBO σ	5	95	0/2/31/67	49/51	NBO π	6	91	0/0/21/79	0/100
						NBO π	6	89	0/0/16/84	0/100
	NBO σ	5	95	0/2/31/67	49/51	NBO π	6	91	0/0/17/83	0/100
						NBO π	6	88	0/0/15/85	0/100
	NBO σ	5	95	0/2/32/64	49/51	NBO π	6	91	0/0/16/84	0/100
						NBO π	6	89	0/0/13/87	0/100
	Imido σ	11	88	1/3/38/59	65/35	Imido π	22	77	0/0/13/87	0/100
						Imido π	22	77	0/0/13/87	0/100

[a] NLMO orbitals do not always add up to 100% because of other minor orbital coefficients. [b] 1-electronX = ligand donor atom, O_{NBO}, O_{THF}, Cl, or N_{imido}.

Table S8. QTAIM data for 1-4.

Cmpd	Bond	ρ^a	$\nabla^2\rho^a$	H^b	ε
**1**	U-O_{NBO} (av.)	0.10	0.37	–0.04	0.07
	U-C (av.)	0.03	0.09	–0.01	0.49
	B-O (av.)	0.20	0.62	–0.15	0.04
	B-N (av.)	0.18	0.21	–0.15	0.17
**2**	U-O_{NBO} (av.)	0.11	0.38	–0.04	0.05
	U-O_{THF} (av.)	0.06	0.21	–0.01	0.29
	U-Cl (av.)	0.06	0.15	–0.02	0.14
	B-O (av.)	0.20	0.63	–0.15	0.04
	B-N (av.)	0.19	0.23	–0.15	0.17
**3**	U-O_{NBO} (av.)	0.11	0.42	–0.04	0.08
	U-O_{THF}	0.06	0.21	–0.01	0.18
	U-Cl	0.08	0.16	–0.03	0.02
	B-O (av.)	0.21	0.66	–0.15	0.04
	B-N (av.)	0.19	0.24	–0.16	0.17
**4**	U-O_{NBO} (av.)	0.11	0.41	–0.04	0.09
	U-N	0.19	0.51	–0.14	0.05
	B-O (av.)	0.21	0.66	–0.15	0.04
	B-N (av.)	0.19	0.24	–0.16	0.17

[a] Units are e per $Bohr^3$ (a.u. = 6.748 e per $Å^3$). [b] Units are Hartrees per $Bohr^3$.

References

1. Avens, L. R.; Bott, S. G.; Clark, D. L.; Sattelberger, A. P.; Watkin, J. G.; Zwick, B. D. A convenient entry into trivalent actinide chemistry: synthesis and characterization of $AnI_3(THF)_4$ and $An[N(SiMe_3)_2]_3$ (An = U, Np, Pu). *Inorg. Chem.* **1994**, *33*, 2248-2256.

2. Stewart, J. L.; Andersen, R. A. Trivalent uranium chemistry: molecular structure of $[(Me_3Si)_2N]_3U$. *Polyhedron* **1998**, *17*, 953-958.

3. Patel, D.; Wooles, A. J.; Hashem, E.; Omorodion, H.; Baker, R. J.; Liddle, S. T. Comments on reactions of oxide derivatives of uranium with hexachloropropene to give UCl_4. *New J. Chem.* **2015**, *39*, 7559-7562.

4. Segawa, Y.; Suzuki, Y.; Yamashita, M.; Nozaki, K. Chemistry of Boryllithium: Synthesis, Structure, and Reactivity. *J. Am. Chem. Soc.* **2008**, *130*, 16069-16079.

5. Loh, Y. K.; Ying, L.; Fuentes, M. A.; Do, D. C. H.; Aldridge, S. An N-Heterocyclic Boryloxy Ligand Isoelectronic with N-Heterocyclic Imines: Access to an Acyclic Dioxysilylene and its Heavier Congeners. *Angew. Chem. Int. Ed.* **2019**, *58*, 4847-4851.

6. Sheldrick, G. M. SHELXT - Integrated space-group and crystal-structure determination. *Acta Cryst. Sect. A* **2015**, *71*, 3-8.
7. CrysAlisPRO version 39.46, Oxford Diffraction /Agilent Technologies UK Ltd, Yarnton, England.
8. Sheldrick, G. M. Crystal structure refinement with SHELXL. *Acta Cryst. Sect. C* **2015**, *71*, 3-8.
9. Dolomanov, O. V.; Bourhis, L. J.; Gildea, R. J.; Howard, J. A. K.; Puschmann, H. OLEX2: a complete structure solution, refinement and analysis program. *J. Appl. Cryst*. **2009**, *42*, 339-341.
10. Farugia, L. J. WinGX and ORTEP for Windows: an update. *J. Appl. Cryst*. **2012**, *45*, 849-854.
11. Persistence of Vision (TM) Raytracer, Persistence of Vision Pty. Ltd., Williamstown, Victoria, Australia.
12. Fonseca Guerra, C.; Snijders, J. G.; Te Velde, G.; Baerends, E. J. Towards an order-N DFT Method. *Theor. Chem. Acc.* **1998**, *99*, 391-403.
13. Te Velde, G.; Bickelhaupt, F. M.; Baerends, E. J.; Fonseca Guerra, C.; Van Gisbergen, S. J. A.; Snijders, J. G.; Ziegler, T. Chemistry with ADF. *J. Comput. Chem.* **2001**, *22*, 931-967.
14. Van Lenthe, E.; Baerends, E. J.; Snijders, J. G. Relativistic regular two-component Hamiltonians. *J. Chem. Phys.* **1993**, *99*, 4597-4610.
15. Van Lenthe, E.; Baerends, E. J.; Snijders, J. G. Relativistic total energy using regular approximations. *J. Chem. Phys.* **1994**, *101*, 9783-9792.
16. Van Lenthe, E.; Ehlers, A. E.; Baerends, E. J. Geometry optimization in the Zero Order Regular Approximation for relativistic effects. *J. Chem. Phys.* **1999**, *110*, 8943-8953.
17. Vosko, S. H.; Wilk, L.; Nusair, M. Accurate spin-dependent electron liquid correlation energies for local spin density calculations: a critical analysis. *Can. J. Phys.* **1980**, *58*, 1200-1211.
18. Becke, A. D. Density-functional exchange-energy approximation with correct asymptotic behaviour. *Phys. Rev. A.* **1988**, *38*, 3098-3100.

19. Perdew, J. P. Density-functional approximation for the correlation energy of the inhomogeneous electron gas. *Phys. Rev. B.* **1986**, *33*, 8822-8824.

20. Grimme, S.; Ehrlich, S.; Goerigk, L. Effect of the damping function in dispersion corrected density functional theory. *J. Comp. Chem.* **2011**, *32*, 1457-1465.

21. Glendening, E. D.; Badenhoop, J. K.; Reed, A. E.; Carpenter, J. E.; Bohmann, J. A.; Morales, C. M.; Landis, C. R.; Weinhold, F. (Theoretical Chemistry Institute, University of Wisconsin, Madison, WI, 2013); http://nbo6.chem.wisc.edu/.

22. Bader, R. F. W. *Atoms in Molecules: A Quantum Theory*, Oxford University Press, New York, 1990.

23. Bader, R. F. W. A bond path: a universal indicator of bonded interactions. *J. Phys. Chem. A* **1998**, *102*, 7314-7323.

24. Motta, L. C.; Autschbach, J. Actinide inverse trans influence versus cooperative pushing from below and multi-center bonding. *Nat. Commun.* **2023**, *14*, 4307.

25. Van Leusen, J.; Speldrich, M.; Schilder, H.; Koegerler, P. Comprehensive insight into molecular magnetism via CONDON: Full vs. effective models. *Coord. Chem. Rev*. **2015**, *289*, 137-148.

26. Speldrich, M.; Van Leusen, J.; Koegerler, P. CONDON 3.0: An Updated Software Package for Magnetochemical Analysis-All the Way to Polynuclear Actinide Complexes. *J. Comput. Chem*. **2018**, *39*, 2133-2145.

27. Reid, M. F-shell program, University of Canterbury, New Zealand, 1984.
